# Examining socioeconomic differences in sepsis risk and mediation by modifiable factors: a Mendelian randomization study

**DOI:** 10.1186/s12879-025-11130-y

**Published:** 2025-05-23

**Authors:** Vilde Hatlevoll Stensrud, Tormod Rogne, Helene Marie Flatby, Randi Marie Mohus, Lise Tuset Gustad, Tom Ivar Lund Nilsen

**Affiliations:** 1 Department of Public Health and Nursing, Norwegian University of Science and Technology, Trondheim, Norway; 2 Mid-Norway Centre for Sepsis Research, Department of Circulation and Medical Imaging, Norwegian University of Science and Technology, Trondheim, Norway; 3 Department of Community Medicine and Global Health, University of Oslo, Oslo, Norway; 4 Department of Chronic Disease Epidemiology, Yale School of Public Health, New Haven, CT USA; 5 Department of Clinical and Molecular Medicine, Norwegian University of Science and Technology, Trondheim, Norway; 6 Clinic of Anaesthesia and Intensive Care, St. Olavs Hospital, Trondheim University Hospital, Trondheim, Norway; 7 Faculty of Nursing and Health Sciences, Nord University, Levanger, Norway; 8 Department of Internal Medicine, Levanger Hospital, Nord-Trøndelag Hospital Trust, Levanger, Norway; 9 Clinic of Emergency Medicine and Prehospital Care, St. Olavs Hospital, Trondheim University Hospital, Trondheim, Norway

**Keywords:** Education, Socioeconomic status, Sepsis, Mendelian randomization, Mediation analysis, Health inequalities, Epidemiology, Modifiable factors

## Abstract

**Background:**

Educational attainment is inversely related to sepsis risk, but the causal nature is still unclear. We therefore conducted the first Mendelian randomization (MR) study of genetically predicted educational attainment on sepsis that also uses a within-family genetic instrument for education. To further explore possible mechanistic pathways that can inform strategies to reduce sepsis risk, we examined the mediating effects of factors that are modifiable or can be prevented.

**Methods:**

The association between genetically predicted educational attainment and sepsis was estimated using summary-level data from recent genome-wide association studies. Possible bias due to population stratification, dynastic effects, and assortative mating in the genetic instrument for education was evaluated using summary-level data from a within-sibship genome-wide association study. We used inverse variance weighted MR analysis to estimate the effect of one standard deviation increase in years of education on sepsis risk. The robustness of the findings was assessed in sensitivity analyses, applying weighted median, weighted mode, and MR Egger regression. Finally, we applied multivariable MR analyses to estimate the mediating effects of smoking initiation, alcohol consumption, body mass index, high-density lipoprotein (HDL)-cholesterol, systolic blood pressure and type 2 diabetes.

**Results:**

For each standard deviation increase in genetically predicted educational attainment (3.4 years), the odds ratio (OR) for sepsis was 0.72 (95% confidence interval (CI) 0.66 to 0.78). The results of the analysis using the within-sibship genetic instrument and other sensitivity analyses were in line with this finding: within-sibship OR 0.88 (95% CI 0.64 to 1.18), weighted median OR 0.70 (95% CI 0.62 to 0.80), weighted mode OR 0.70 (95% CI 0.43 to 1.13), and MR Egger OR 0.65 (95% CI 0.50 to 0.85). The mediation analysis showed that 56% of the effect of educational attainment on sepsis risk can be explained by modifiable or preventable factors.

**Conclusions:**

Higher educational attainment is strongly associated with a reduced risk of sepsis, pointing to important socioeconomic differences in this disease. The results also suggest that interventions targeting modifiable or preventable factors could contribute to reducing the socioeconomic differences in sepsis risk.

**Supplementary Information:**

The online version contains supplementary material available at 10.1186/s12879-025-11130-y.

## Background

Sepsis affects 48.9 million people annually and accounts for one in five deaths worldwide [1]. To reduce sepsis incidence and mortality, the World Health Organization (WHO) has called for increased knowledge of factors contributing to lowering the burden of sepsis [2]. Current evidence points to considerable socioeconomic differences in sepsis risk [3] that could be explained by health-related behaviour, chronic diseases, access to health care, and exposure to pathogens [4, 5]. Inverse associations have been reported for risk of bacteraemia [6], intensive care unit admission for sepsis [7], as well as incidence and mortality of sepsis [8, 9]. However, the knowledge of factors that can be targeted to mitigate socioeconomic differences in sepsis risk is limited. Smoking, alcohol consumption, cardiovascular disease risk factors, and existing chronic diseases have been reported as potentially mediating factors [10, 11].

The knowledge about socioeconomic differences in sepsis, and also about possible mediators of this association, stems largely from conventional observational studies that are prone to bias by confounding and reverse causation [12]. There is therefore a need for studies with different methodological assumptions that can aid in the triangulation of evidence needed for well-founded policy and health care decisions. Evidence from Mendelian randomization (MR) studies can contribute to this by using genetic variants as instrumental variables (IVs) to explore the association between educational attainment and the risk of sepsis [13]. A recent two-sample MR study [11] reported an inverse association between genetically predicted educational attainment and sepsis risk. This evidence can be further triangulated by applying MR analysis using within-family genetic instruments to reduce bias from dynastic effects, where parental phenotypes directly affect offspring phenotypes, assortative mating, where partner selection is related to phenotypic characteristics, and population stratification, when both genotype and phenotype is correlated with ancestry [13, 14]. All three sources of bias may be particularly relevant in studies of educational attainment. In this type of analysis, external factors are held constant within sibling pairs, and genetic differences thus represent the random and independent assignment of parental alleles [14].

The aim of this two-sample MR study is to examine the association between genetically predicted educational attainment and the risk of sepsis using genetic instruments derived from both population-based and within-family data, and explore possible mediation by genetically determined risk factors for sepsis that are either modifiable or preventable, such as smoking initiation, alcohol consumption, body mass index (BMI), blood lipids, blood pressure, and diabetes.

## Methods

### Data sources

This two-sample MR study is based on publicly available summary-level data from the largest and most recent genome-wide association studies (GWAS) on sepsis, educational attainment, smoking initiation, alcohol consumption, BMI, high-density lipoprotein (HDL)-cholesterol, systolic blood pressure and type 2 diabetes (Table 1). All GWAS data are subjects of European ancestry. The reporting from this study is in accordance with the STROBE-MR guidelines [15].

**Table 1:** Overview of genome-wide association studies included in the analyses

Trait (reference)	Phenotype definition	Cohorts	No. of participants^a^	Ancestry	Covariate adjustments
**Exposures**					
Education [16]	Self-reported years of education (number of years of schooling) (SD = 3.4 years)	71 cohorts, including 23andMe and UK Biobank	3,037,499	European	Sex, a third-degree polynomial in birth year and interactions with sex, the top 40 PCs, and batch dummies
Education (within-sibship) [17]	Self-reported years of education (number of years of schooling) (SD = 1 year)	19 cohorts, including UK Biobank and HUNT	128,777	European	Age, sex and principal components.
**Mediators**					
Alcohol consumption [18]	Self-reported drinks per week (number of drinks per week)	60 cohorts, including UK Biobank and GERA	666,978	European	Age, age squared, sex, and genetic principal components
Body mass index [19]	Measured height and weight at study participation (kg/m^2^)	UK Biobank	681,275	European	Age, sex, recruitment centre, genotyping batches and 10 PCs.
Smoking initiation [18]	Self-reported smoking behavior (ever regular smoker vs. never regular smoker)	60 cohorts, including UK Biobank and deCODE	805,431	European	Age, age squared, sex, and genetic principal components
Type 2 diabetes [22]	HbA1c or fasting glucose, recorded or self-reported diagnosis, or anti-diabetic medication (cases vs. controls)	32 cohorts, including UK Biobank and deCODE	80,154/853,816	European	Age, sex, genomic control, and PCs
HDL cholesterol [20]	Measured blood lipid levels (after > 8 h of fasting) (mg/dL)	60 cohorts, including deCODE and Go-DARTS	188,577	European	Age, age squared, and sex.
Systolic blood pressure [21]	Measured systolic blood pressure (mmHg)	78 cohorts, including UK Biobank and OMICS-EPIC	757,601	European	Age, age squared, sex, BMI, medication use, and a binary indicator variable that accounts for different genotyping chips and different study ascertainment.
**Outcome**					
Sepsis [23]	Explicit sepsis diagnosis codes (cases vs. controls)	UK Biobank	10,154/454,764	European	Age, sex, and genotyping chip

^a^Including cases/controls

Abbreviations: HDL, high-density lipoprotein; SD, standard deviation; HbA1c, hemoglobin A1c; UK, United Kingdom; HUNT, The Trøndelag Health Study; GERA, Genetic Epidemiology Research on Aging; Go-DARTS, Genetics Audit and Research Tayside Scotland

Genetically predicted educational attainment was obtained from a meta-analysed GWAS and defined as attained years of education and mapped according to an International Standard Classification of Education 1997 category. The mean and standard deviation (SD) of education were 15.4 and 3.4 years, respectively. The results from the meta-analysis found genetic correlations between the GWAS of education (new 23andMe, updated UK Biobank results, and EA3) close to 1, yet statistically different from it, suggesting some heterogeneity [16]. Since complete summary-level data was unavailable, we used a restricted sample that includes clumped single nucleotide polymorphisms (SNPs) with a p-value less than 1 × 10^− 5^. For the MR analysis using a within-family derived genetic instrument, summary-level data of educational attainment was retrieved from the IEU OpenGWAS from a within-sibship meta-analysed GWAS (GWAS identifier: ieu-b-4835) [17]. This meta-analysed data used standardized normal units of education from each contributing cohort; thus, the number of years constituting a standard deviation was not reported. Since intervening on educational attainment at the individual level represents significant challenges, we chose to focus on six modifiable and/or preventable factors as possible mediators: smoking initiation (ever vs. never regular smokers) [18], alcohol consumption (number of drinks per week) [18], BMI (kg/m^2^) [19], HDL-cholesterol (mg/dL) [20], systolic blood pressure (mmHg) [21], and type 2 diabetes (cases vs. controls) [22]. Smoking initiation and type 2 diabetes were binary variables, whereas the other mediators were continuous variables. Summary-level data on genetic susceptibility to sepsis was obtained from the IEU OpenGWAS from the UK Biobank (GWAS identifier: ieu-b-69) [23, 24]. The data included 10,154 cases of sepsis and 454,764 controls of European ancestry. The definition of sepsis was based on a set of explicit International Classification of Disease (ICD) 9 and 10 codes previously described by Rudd et al. [1]. (See Supplementary Table S1, Supplementary material 1).

Only SNPs that were not in linkage disequilibrium (LD) with the other SNPs (*r*^*2*^ < 0.01) and at a genome-wide significant level (*p* < 5.0 × 10^− 8^) were included in our analyses. This *r*^*2*^ value was chosen to ensure a balance between maintaining SNP independence and retaining a sufficient number of SNPs for the genetic instrument [25–27]. In the additional analysis of within-sibship genetically predicted educational attainment, as well as in a bidirectional MR, suggestive genome-wide significant SNPs (*p* < 5.0 × 10^− 6^) were included in the analysis as there were no or few genome-wide significant SNPs available. The reduced p-value threshold might contribute to including SNPs that are not strongly related to the exposure of interest.

This study was performed with the use of publicly available summary-level data with relevant ethical approval. The use of publicly available summary-level data does not require regional ethical committee (REC) approval.

### Statistical analyses

Our main analysis was performed by univariable MR analysis using the inverse variance weighted (IVW) method to estimate the effect of genetically predicted educational attainment on the risk of sepsis. In the bidirectional MR assessing possible reverse causation or shared genetic liabilities, sepsis was used as the exposure and educational attainment as the outcome [13].

The IVW method assumes that all genetic instruments are valid [28]. For an IV to be valid, three key assumptions must be met: it must be strongly associated with the exposure (relevance), cannot share a common cause with the outcome (independence), and only affects the outcome through the exposure (exclusion restriction) [13]. The first condition was tested via a first-stage F-statistic [12, 29]. The strength of each SNP was assessed by the F-statistic, which was calculated as F = R^2^(N– 2)/(1– R^2^), where N is the sample size of the GWAS on educational attainment and R^2^ is the proportion of variance in educational attainment explained by the genetic variants [29]. The proportion of variance explained by the genetic instruments was calculated via the following formula: R^2^ = 2 x MAF(1– MAF)beta^2^, where MAF is the minor allele frequency and beta is the effect estimate of the genetic variant in the exposure, given in standard deviation units [30]. To limit the risk of weak instrument bias, no SNPs associated with educational attainment and sepsis with a F-statistic < 10 were included. The exposure GWAS was adjusted for sex, a third-degree polynomial in birth year and interactions with sex, the top 40 PCs, and batch dummies, while the outcome GWAS was adjusted for age, sex and genotyping chip. We conducted several sensitivity analyses to evaluate the second and third conditions, including the weighted median, weighted mode, and MR Egger regression. These analyses assess possible evidence of horizontal pleiotropy, i.e., when one genetic variant influences several traits [31]. In the weighted median analysis, each IV is given a weight on the basis of its precision, and an MR estimate is produced on the basis of its median value. The analysis gives a consistent overall estimate when at least half of the weights are valid instruments [32, 33]. The weighted mode is the most frequent value of a weighted empirical distribution of the estimates from the IVW analysis. It provides a reliable estimate of the true causal effect when the largest weights of the IVs are estimated and allows some invalid IVs [34]. The weighted median and the weighted mode are robust to outlying genetic variants [33]. MR Egger regression allows IVs to be invalid or have pleiotropic effects as long as the InSIDE assumption holds [35]. The InSIDE assumption states that any pleiotropic effect of an IV is independent of the strength of the IVs effect on the exposure. MR Egger is also sensitive to outlying genetic variants [33]. The MR Egger intercept test assesses whether balanced or unbalanced pleiotropy is present. An intercept close to zero indicates balanced pleiotropy [36]. In a supplementary analysis, we also used *r*^*2*^ < 0.001 to ensure that our analyses had adequate SNP independence.

Population stratification, dynastic effects, and assortative mating may open paths from the genetic instrument to the outcome and thereby violates the independence assumption of a valid IV. These biasing paths can be closed by controlling for sibling, mother or father genotype [14]. We therefore conducted MR analysis using within-sibship GWAS data on educational attainment [13, 14]. This analysis used the IVW method to estimate the effect of educational attainment within-sibship on the risk of sepsis.

**Fig. 1:**
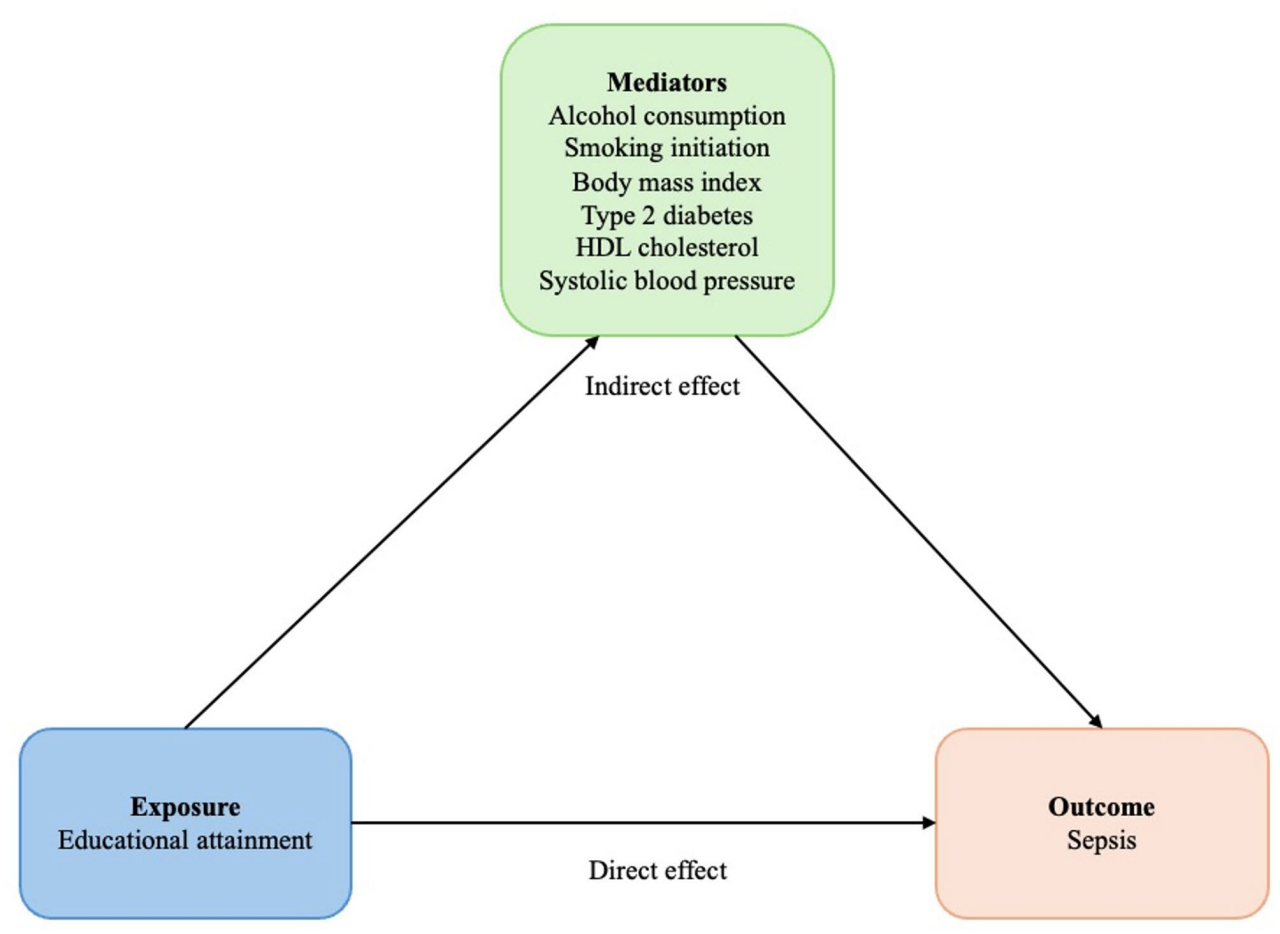
Mediation analysis. Abbreviations: HDL, high-density lipoprotein

The mediation analyses include four analytical steps: (1) the association between educational attainment and the risk of sepsis (i.e., total effect), (2) the association between educational attainment and each of the mediators, (3) the association between each mediator and the risk of sepsis, and (4) the association between educational attainment and the risk of sepsis after accounting for the mediators (i.e., direct effect). Univariable MR analysis was used for the first three steps, whereas the fourth step was based on multivariable MR. This analysis estimates the extent to which each mediator, separately and combined, mediates the association between educational attainment and sepsis risk (Fig. 1) [13]. The summary-level data from each GWAS were harmonized prior to the analysis to ensure that the effect estimate of each IV was oriented to the same allele in all files. The conditional F statistic was calculated for all traits in all multivariable MR analyses according to Sanderson et al. [37]. The IVW method estimated the total effect of educational attainment on the risk of sepsis using only SNPs that were associated with both educational attainment and all the mediators (and thus included fewer IVs than used in the main analysis). By controlling for the mediators, the analysis can differentiate between direct and indirect effects. The direct effect of educational attainment on the risk of sepsis was obtained by including the mediators in the statistical model, both individually and all combined. The indirect effect was then estimated by the difference method by subtracting the direct effect from the total effect [38]. We chose this method due to ease of interpretation [39] and since it has been reported to have largely similar efficiency as other available methods [40]. The proportion mediated was calculated by subtracting the beta coefficient from the univariable MR using the beta coefficient from the multivariable MR and then dividing it by the beta coefficient from the univariable MR. The standard errors were estimated using bootstrapping [41] (see example code in Supplementary material 1).

All statistical analyses were conducted using the *TwoSampleMR* package (version 0.6.7), *MendelianRandomization* package (version 0.10.0), and *MVMR* package (version 0.4) in R (version 4.3.3) [28, 37, 42].

## Results

A total of 3,037,499 individuals were included in the GWAS on educational attainment, whereas the sepsis GWAS included 10,154 cases. After the exposure and outcome datasets were harmonized, 1,716 SNPs were included in the main analysis (Supplementary List S1, Supplementary material 1). The explained variance of the IVs for educational attainment was 7.5%. The F-statistic was above 10 for all the IVs (range 30–572).

**Fig. 2:**
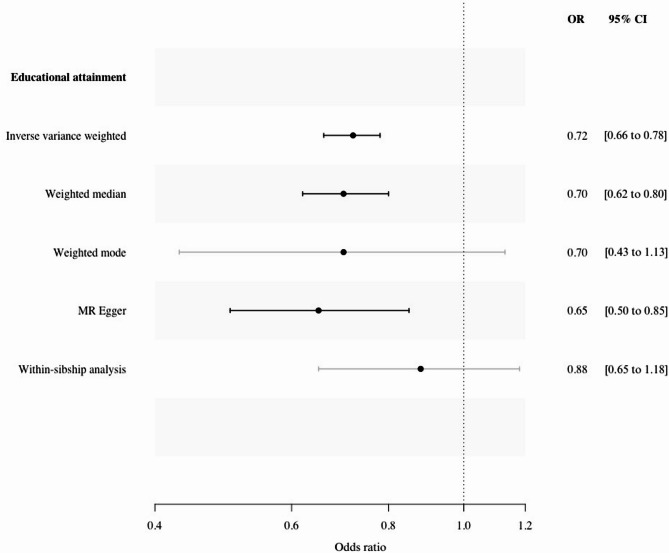
Odds ratio (OR) of sepsis according to a standard deviation (3.4 years) increase in genetically predicted educational attainment and educational attainment within-sibship. The analysis using within-sibship data was carried out using the inverse variance weighted method. Abbreviations: OR, odds ratio; CI, confidence interval; MR, Mendelian randomization

In the univariable IVW analysis, the odds ratio (OR) for sepsis was 0.72 (95% confidence interval (CI), 0.66 to 0.78) for each SD (3.4 years) increase in genetically predicted educational attainment (Fig. 2). There was no evidence of reverse causation or shared genetic liabilities between genetically predicted risk of sepsis and educational attainment (Supplementary Table S2, Supplementary material 1). The results of the weighted median, weighted mode and MR Egger approaches aligned with the findings of the IVW analysis. The MR Egger intercept did not detect unbalanced pleiotropy (Supplementary Table S3, Supplementary material 1). The analyses using *r*^*2*^ threshold of < 0.001 yielded results consistent with the main analyses, indicating no evidence of insufficient SNP independence (Supplementary Table S4, Supplementary material 1). The analysis using the within-sibship genetic instrument for educational attainment included eleven SNPs, and the results of the IVW analysis suggest that educational attainment is inversely, but imprecisely, related to the risk of sepsis (Fig. 2).

Genetically predicted educational attainment was statistically significantly associated with all mediators in the IVW analyses (Supplementary Figure S1, Supplementary material 1). Genetically predicted educational attainment was inversely related to smoking initiation, BMI, type 2 diabetes and systolic blood pressure and positively related to alcohol consumption and HDL-cholesterol. The sensitivity analyses supported the results from the IVW analyses (Supplementary Table S5, Supplementary material 1). Genetically predicted alcohol consumption, smoking initiation, BMI, and type 2 diabetes were positively associated with risk of sepsis, whereas genetically predicted HDL-cholesterol was inversely associated with sepsis risk (Supplementary Figure S2, Supplementary material 1). There was no clear association between genetically predicted systolic blood pressure and the risk of sepsis. The sensitivity analyses supported the findings from the IVW analyses (Supplementary Table S6, Supplementary material 1).

**Fig. 3:**
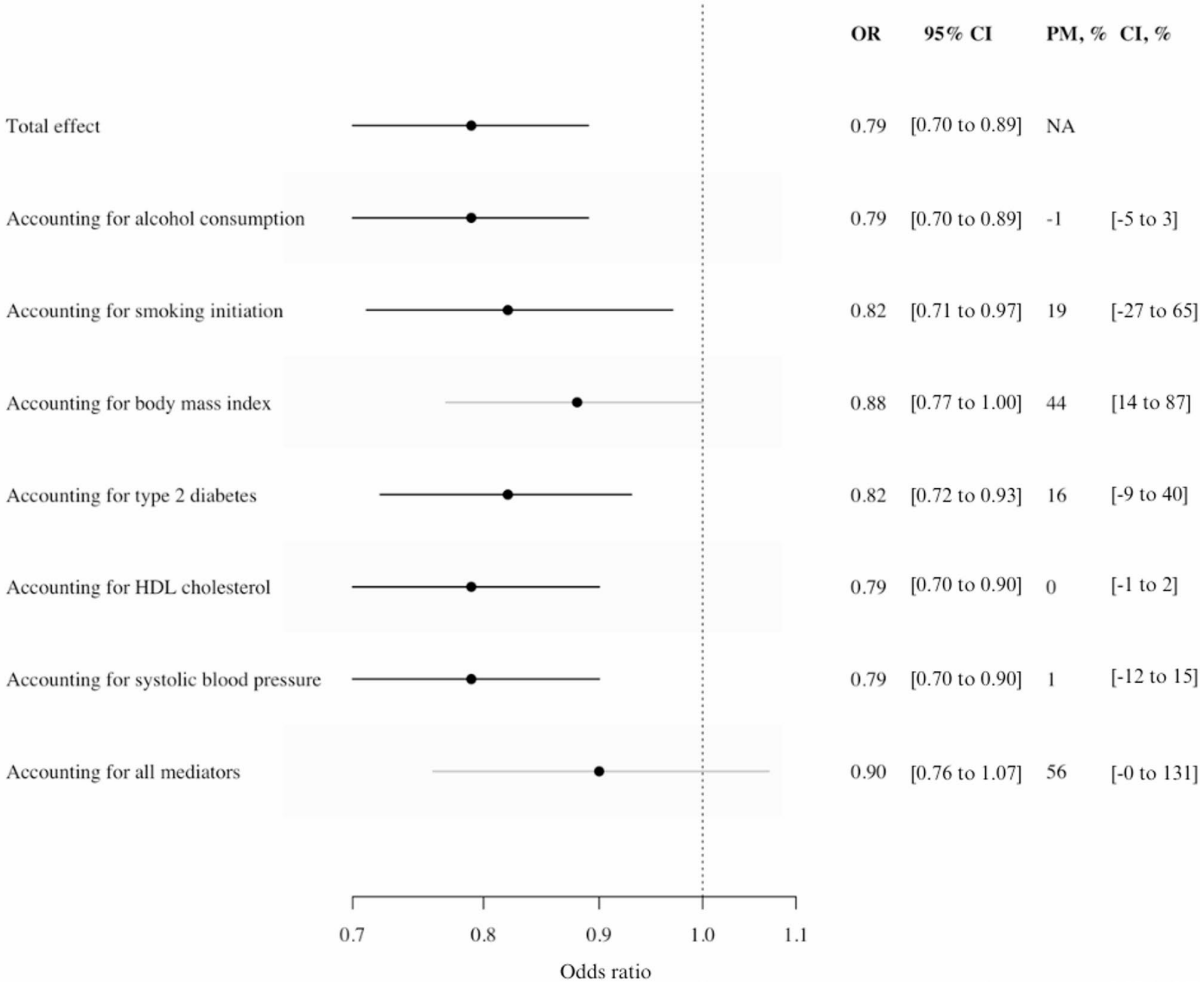
Odds ratio (OR) of sepsis according to a standard deviation (3.4 years) increase in genetically predicted educational attainment, after accounting for modifiable and/or preventable mediating factors. Abbreviations: OR, odds ratio; CI, confidence interval; PM, proportion mediated; HDL, high-density lipoprotein

431 SNPs were included in the multivariable MR analyses (Supplementary List S2, Supplementary material 1). The conditional F-statistic ranged from 1.46 to 61.76 for the multivariable analyses (Supplementary Table S7, Supplementary material 1). Combined, all the modifiable and/or preventable mediators accounted for 56% (95% CI, -0 to 131%) of the association between genetically predicted educational attainment and the risk of sepsis (Fig. 3). Separately, BMI accounted for 44% (95% CI, 14 to 87%), smoking initiation accounted for 19% (95% CI, -27 to 65%), type 2 diabetes accounted for 16% (95% CI, -9 to 40%), alcohol consumption accounted for − 1% (95% CI, -5 to 3), HDL-cholesterol accounted for 0% (95% CI, -1 to 2), and systolic blood pressure accounted for 1% (95% CI, -12 to 15) of the association between genetically predicted educational attainment and the risk of sepsis (Fig. 3).

## Discussion

In this two-sample MR study, genetically predicted educational attainment was inversely related to sepsis. A genetically estimated 3.4-year increase in educational attainment was associated with a 28% reduction in sepsis risk, and this was consistent across various sensitivity analyses. Although the results using the within-family genetic instrument for education had lower power they are in line with main results. Overall, genetically predicted smoking initiation, alcohol consumption, BMI, HDL-cholesterol, systolic blood pressure, and type 2 diabetes mediated approximately half of the effect of educational attainment on sepsis risk.

To our knowledge, only one MR study has investigated the effect of educational attainment on the risk of sepsis and assessed potential mediating factors [11]. They reported weaker effect estimates than did the present study and found substantial mediation by BMI and smoking but not by apolipoprotein AI and omega-3. Our study confirms that BMI is a strong mediating factor between education and sepsis risk, but also identifies other modifiable and preventable factors that can be targets for sepsis prevention. This is the first MR study to report a possible mediating effect of type 2 diabetes on the association between educational attainment and sepsis risk, indicating that reducing socioeconomic differences in diabetes risk and disease management might contribute to sepsis prevention. Furthermore, the previous MR study was based on summary-level GWAS data that included only a third of the population for educational attainment compared with the present study. This could have led to a less precise exposure classification. As expected when the included mediators are correlated [43], the combined mediating effect of all mediators was lower than the sum of the mediating effect of each mediator separately. Finally, studies of educational attainment as a risk factor are particularly prone to bias by population stratification, dynastic effects and assortative mating [17]. The within-sibship results from the present study further strengthen the evidence that educational attainment is inversely related to sepsis risk [13, 14, 17], although this estimate had limited statistical power and could be prone to weak instrument bias.

### Clinical and public health implications

Education is suggested to influence sepsis risk through several pathways [5]. These include exposure to different exogenic factors, such as pathogens, toxins and stress, that can alter hormonal and immune responses [5]. Additionally, lifestyle and health-related behaviour can lead to a disadvantageous health profile that in turn may cause physiological changes and ultimately chronic diseases [4, 5], all of which may predispose individuals to sepsis. Reducing socioeconomic differences in health is mainly a political responsibility [44, 45], but interventions targeting modifiable and/or preventable mediating factors of the adverse effects of low educational attainment on sepsis risk can also be beneficial. Thus, knowledge of such factors that may contribute to reducing socioeconomic inequalities in sepsis risk is highly important for health care providers and for individual persons.

### Strengths and limitations

The main strength of this two-sample MR study is the possibility of reducing bias due to residual confounding and reverse causation, which often hamper the validity of conventional observational studies. Other important strengths include the use of the largest and most recent publicly available GWAS summary-level data on exposures, mediators, and outcomes, which increase the power of our analyses. The F-statistic was above 10 for all IVs in the main analyses, indicating that weak instrument bias is unlikely to influence the results and that the IVs did satisfy the relevance assumption. Moreover, the results of the different sensitivity analyses were in line with the estimates from the main analysis. This is also supported by the results that were based on a within-sibship GWAS of educational attainment, thus accounting for potential bias due to population stratification, dynastic effects, and assortative mating in the genetic instrument. We included only the genetic data of populations with the same European ancestry, which also reduces bias from population stratification. Education as an indicator of socioeconomic status has the advantage that it is relatively easy to measure and is not affected by diseases that occur in late adult life [46]. In contrast, it might be affected by diseases with early-life onset.

Several limitations are important to consider. First, since populations of European ancestry were used, the results cannot be generalized to other populations and ethnicities. As mentioned earlier, sepsis occurs worldwide, and similar analyses should be conducted in different populations. Second, we used suggestive genome-wide significant SNPs from the within-family GWAS since no genome-wide significant SNPs were discovered. This might result in SNPs not strongly related to education, and thus the results could be prone to weak instrument bias and low power. Nevertheless, these results contribute to further triangulation of evidence for the effect of educational attainment and risk of sepsis. The lower p-value threshold was also used in the bidirectional MR analysis and the results related to shared genetic liability and reverse causation could therefore be influenced by weak instrument bias. Third, some overlap of the participants in the datasets of our exposure, mediators, and outcome may have introduced bias [47]. Nevertheless, it has been argued that two-sample MR methods can be used in one-sample MR studies conducted in large biobanks [48]. Our study used outcomes from the UK Biobank and exposure from a meta-analysis of several biobanks, including the UK Biobank, and the maximum potential overlap between the datasets is 0.3% (no. of cases in sepsis GWAS / no. of participants in education GWAS x 100). Unfortunately, complete summary-level data on educational attainment was not available, and MRlap analysis could not be conducted [49]. Fourth, the conditional F-statistic were less than 10 for several traits in the multivariable MR analysis, indicating that the included factors might be correlated and that there is a potential for weak instrument bias and pleotropic effects of the IVs [37]. Given the restricted summary-level data on educational attainment, genome-wide significant SNPs in the mediator GWAS that were not present in the educational attainment GWAS were excluded from the analysis. Consequently, this led to lower conditional F-statistics for the mediators. Fifth, it is essential to mention that MR studies measure the odds of an outcome from a lifetime perspective and should be interpreted that way. Additionally, we cannot conclude that interventions aimed at educational attainment would have reduced the risk of sepsis.

## Conclusions

The results of this MR study provide robust evidence for an inverse association between educational attainment and the risk of sepsis. We found that BMI, smoking initiation, and type 2 diabetes are strong mediators of the association between educational attainment and sepsis risk, and these factors explained half of the effect of education on sepsis risk. Interventions targeting modifiable mediating factors could contribute to reducing socioeconomic disparities in sepsis and lowering the burden of this disease.

## Electronic supplementary material

Below is the link to the electronic supplementary material.

Supplementary Material 1

## Data Availability

The summary-level data used in this study are publicly available and may be downloaded from the Consortiums’ websites. UK Biobank, https://gwas.mrcieu.ac.uk/datasets/ieu-b-69/. SSGAC, https://www.thessgac.org. Within Family Consortium, https://gwas.mrcieu.ac.uk/datasets/ieu-b-4835/. GSCAN, https://conservancy.umn.edu/items/91f6a003-6af2-4809-9785-53dc579dc788. GIANT Consortium, https://portals.broadinstitute.org/collaboration/giant/index.php/GIANT_consortium_data_files#2018_GIANT_and_UK_BioBank_Meta-analysis. Global Lipids Gentics Consortium, https://csg.sph.umich.edu/willer/public/lipids2013/. DIAMANTE, https://diagram-consortium.org/downloads.html. For systolic blood pressure, summary-level data can be assessed through request to the authors, Paul Elliott (p.elliott@imperial.ac.uk) or Mark Caulfield (m.j.caulfield@qmul.ac.uk) (please see the original article, https://www.nature.com/articles/s41588-018-0205-x).

## Abbreviations

WHO: World Health Organization
MR: Mendelian randomization
IVs: Instrumental variables
BMI: Body mass index
GWAS: Genome-wide association studies
HDL: High-density lipoprotein
SD: Standard deviation
SNP: Single nucleotide polymorphism
ICD: International Classification of Disease
LD: Linkage disequilibrium
REC: Regional ethical committee
IVW: Inverse variance weighted
OR: Odds ratio
CI: Confidence interval
