# Supplementary material for: Examining socioeconomic differences in sepsis risk and mediation by modifiable factors: a Mendelian randomization study
Source: BMC Infect Dis. 2025 May 23;25:739. doi: 10.1186/s12879-025-11130-y (PMC12103053; doi:10.1186/s12879-025-11130-y)
Supplement: Supplementary file 1 — Supplementary Material 1 [file 12879_2025_11130_MOESM1_ESM.pdf]

Supplementary material 1

Examining socioeconomic differences in sepsis risk and mediation by modifiable factors: A Mendelian randomization study

Vilde Hatlevoll Stensrud[1,2],
Tormod Rogne[3,4],
Helene Marie Flatby[2,5],
Randi Marie Mohus[2,6],
Lise Tuset Gustad[7,8],
Tom Ivar Lund Nilsen[1,9],

1) Department of Public Health and Nursing, Norwegian University of Science and Technology, Trondheim, Norway
2) Mid-Norway Centre for Sepsis Research, Department of Circulation and Medical Imaging, Norwegian University of Science and Technology, Trondheim, Norway
3) Department of Community Medicine and Global Health, University of Oslo, Oslo, Norway
4) Department of Chronic Disease Epidemiology, Yale School of Public Health, New Haven, CT, USA.
5) Department of Clinical and Molecular Medicine, Norwegian University of Science and Technology, Trondheim, Norway
6) Clinic of Anaesthesia and Intensive Care, St. Olavs Hospital, Trondheim University Hospital, Trondheim, Norway
7) Faculty of Nursing and Health Sciences, Nord University, Levanger, Norway
8) Department of Internal Medicine. Levanger Hospital, Nord-Trøndelag Hospital Trust, Levanger, Norway
9) Clinic of Emergency Medicine and Prehospital Care, St. Olavs Hospital, Trondheim University Hospital, Trondheim, Norway

Corresponding author: Vilde Hatlevoll Stensrud, vilde.h.stensrud@ntnu.no

Example code for calculation of standard errors of the proportion mediated

```
bm1 <- allExposures_wOutcome$Beta_Alc
bmse1 <- allExposures_wOutcome$SE_Alc
bm2 <- allExposures_wOutcome$Beta_SBP
bmse2 <- allExposures_wOutcome$SE_SBP
bm3 <- allExposures_wOutcome$Beta_BMI
bmse3 <- allExposures_wOutcome$SE_BMI
bm4 <- allExposures_wOutcome$Beta_Smk
bmse4 <- allExposures_wOutcome$SE_Smk
bm5 <- allExposures_wOutcome$Beta_HDL
bmse5 <- allExposures_wOutcome$SE_HDL
bm6 <- allExposures_wOutcome$Beta_T2DM
bmse6 <- allExposures_wOutcome$SE_T2DM

set.seed(31415)
straps = 1e5; total=NULL; direct=NULL
for (j in 1:straps) {
bx.boot   = rnorm(length(bx), bx, bxse)
bm.boot1  = rnorm(length(bm1), bm1, bmse1)
bm.boot2  = rnorm(length(bm2), bm2, bmse2)
bm.boot3  = rnorm(length(bm3), bm3, bmse3)
bm.boot4  = rnorm(length(bm4), bm4, bmse4)
bm.boot5  = rnorm(length(bm5), bm5, bmse5)
bm.boot6  = rnorm(length(bm6), bm6, bmse6)
by.boot   = rnorm(length(by), by, byse)
total[j]  = lm(by.boot~bx.boot-1, weights=byse^-2)$coef[1]
direct[j] = lm(by.boot~bx.boot+bm.boot1+bm.boot2+bm.boot3+bm.boot4+bm.boot5+bm.boot6-1, weights=byse^-2)$coef[1]
}
mediated = (total-direct)/total
```

Supplementary Table S1: Explicit ICD-9 and -10 codes of sepsis [1]

ICD-9	ICD-10
038-038.9. 090-097.9. 286.6. 635-639.9. 646.5-646.64. 658.4-658.93. 659.2-659.33. 670-670.9. 672-672.04. 674.1-674.34. 675-675.94. 771. 771.4-771.89. 800-801.99. 803-804.99. 905.0. 995.9-995.94	A02.1-A02.9. A20.7-A20.9. A21.7-A21.9. A22.7-A22.9. A24.1-A24.9. A26.7-A26.9. A28.2-A28.9. A32.7-A32.9. A39.0. A39.4-A41.9. A42.7-A42.9. A50-A50.9. A54.86. B00.7-B00.9. B37.7-B37.9. N98.0. O03.0. O03.38. O03.5. O03.88. O04.5. O04.88. O07.38. O08.0. O08.83. O23-O23.93. O41.1-O41.93. O75.3. O85-O86.89. O88.3-O88.32. O91-O91.23. O98. O98.2-O98.93. P00.2. P22-P23.9. P29.12. P29.81. P35-P37. P37.1-P39.9. R65.2-R65.21. R68.13

ICD, International Classification of Diseases

Supplementary Table S2: Odds ratio (OR) of educational attainment according to a log(odds) unit increase in genetically predicted risk of sepsis

	**Number of SNPs**	**OR (95% CI)**	**P-value**
**Inverse variance weighted**	30	1.00 (0.98 to 1.01)	0.38

SNP, single nucleotide polymorphism; OR, odds ratio; CI, confidence interval

Supplementary Table S3: MR egger intercept. Main analysis of the association between genetically predicted educational attainment and risk of sepsis

Exposure	Number of SNPs	MR Egger intercept	P-value
**Educational attainment**	1716	-0.001	0.46

SNPs, single nucleotide polymorphisms; MR, Mendelian randomization

Supplementary Table S4: Odds ratio (OR) of sepsis according to a standard deviation (3.4 years) increase in genetically predicted educational attainment using r^2 <0.001

	Number of SNPs	OR (95% CI)	P-value
**Inverse variance weighted**	552	0.72 (0.63 to 0.82)	4.81E-07
**Weighted median**	552	0.66 (0.55 to 0.81)	3.32E-05
**Weighted mode**	552	0.58 (0.35 to 0.95)	0.03
**MR Egger**	552	0.79 (0.53 to 1.16)	0.23

SNP, single nucleotide polymorphism; OR, odds ratio; CI, confidence interval

Supplementary Table S5: Beta coefficients of modifiable mediating factors according to a standard deviation (SD) (3.4 years) increase in genetically predicted educational attainment. The mediators are in log(odds) units for smoking and diabetes type 2 and in SD units for alcohol consumption, body mass index, HDL cholesterol and systolic blood pressure

**Trait**/ statistical test	**Number of SNPs**	**Beta coefficient (95% CI)**	**P-value**
**Alcohol consumption**			
Inverse variance weighted	1724	0.03 (0.02 to 0.05)	9.97E-05
Weighted median	1724	0.02 (0.00 to 0.04)	1.33E-02
Weighted mode	1724	0.00 (-0.09 to 0.08)	9.47E-01
MR Egger	1724	0.06 (0.01 to 0.12)	1.92E-01
**Smoking initiation**			
Inverse variance weighted	1722	-0.31 (-0.33 to -0.29)	5.43E-253
Weighted median	1722	-0.27 (-0.29 to -0.25)	9.22E-189
Weighted mode	1722	-0.27 (-0.37 to -0.18)	4.09E-08
MR Egger	1722	-0.29 (-0.35 to -0.24)	4.93E-24
**Body mass index**			
Inverse variance weighted	993	-0.27 (-0.31 to -0.24)	1.07E-53
Weighted median	993	-0.24 (-0.26 to -0.21)	7.00E-72
Weighted mode	993	-0.24 (-0.37 to -0.11)	3.19E-04
MR Egger	993	-0.30 (-0.43 to -0.18)	2.69E-06
**Type 2 diabetes**			
Inverse variance weighted	1729	-0.59 (-0.65 to -0.53)	3.15E-79
Weighted median	1729	-0.59 (-0.65 to -0.52)	8.08E-67
Weighted mode	1729	-0.51 (-0.83 to -0.19)	1.66E-03
MR Egger	1729	-0.71 (-0.90 to -0.51)	1.36E-12
**HDL cholesterol**			
Inverse variance weighted	899	0.15 (0.10 to 0.20)	3.03E-08
Weighted median	899	0.15 (0.09 to 0.21)	1.04E-06
Weighted mode	899	0.19 (-0.04 to 0.41)	1.00E-01
MR Egger	899	0.18 (-0.01 to 0.37)	5.96E-02
**Systolic blood pressure**			
Inverse variance weighted	1644	-1.89 (-2.27 to -1.51)	2.21E-22
Weighted median	1644	-1.63 (-1.96 to -1.30)	3.84E-22
Weighted mode	1644	-0.67 (-2.25 to 0.91)	4.06E-01
MR Egger	1644	-0.92 (-2.14 to 0.30)	1.38E-01

SNPs, single nucleotide polymorphisms; OR, odds ratio; CI, confidence interval; MR, Mendelian randomization; HDL, high-density lipoprotein

Supplementary Table S6: Odds ratio (OR) of sepsis according to a log(odds) unit increase in genetically predicted smoking and type 2 diabetes, and a standard deviation increase in genetically predicted alcohol consumption, body mass index, HDL cholesterol and systolic blood pressure

**Trait**/ statistical test	**Number of SNPs**	**OR (95% CI)**	**P-value**
**Alcohol consumption**			
Inverse variance weighted	81	1.18 (0.89 to 1.56)	0.26
Weighted median	81	1.57 (1.06 to 2.32)	0.03
Weighted mode	81	1.69 (0.83 to 3.46)	0.15
MR Egger	81	1.84 (0.75 to 4.51)	0.18
**Smoking initiation**			
Inverse variance weighted	272	1.60 (1.36 to 1.88)	0.00
Weighted median	272	1.53 (1.19 to 1.95)	0.00
Weighted mode	272	1.07 (0.48 to 2.37)	0.87
MR Egger	272	1.06 (0.53 to 2.10)	0.88
**Body mass index**			
Inverse variance weighted	939	1.29 (1.19 to 1.39)	0.00
Weighted median	939	1.24 (1.10 to 1.39)	0.00
Weighted mode	939	1.14 (0.85 to 1.52)	0.39
MR Egger	939	1.39 (1.10 to 1.75)	0.01
**Type 2 diabetes**			
Inverse variance weighted	190	1.04 (0.99 to 1.08)	0.09
Weighted median	190	1.01 (0.95 to 1.07)	0.81
Weighted mode	190	1.01 (0.93 to 1.11)	0.77
MR Egger	190	0.97 (0.88 to 1.07)	0.60
**HDL cholesterol**			
Inverse variance weighted	116	0.94 (0.87 to 1.01)	0.10
Weighted median	116	0.93 (0.83 to 1.04)	0.21
Weighted mode	116	0.94 (0.83 to 1.07)	0.34
MR Egger	116	0.94 (0.82 to 1.07)	0.36
**Systolic blood pressure**			
Inverse variance weighted	671	1.00 (1.00 to 1.01)	0.47
Weighted median	671	1.00 (1.00 to 1.01)	0.22
Weighted mode	671	1.00 (0.99 to 1.02)	0.61
MR Egger	671	1.00 (0.99 to 1.02)	0.46

SNPs, single nucleotide polymorphisms; OR, odds ratio; CI, confidence interval; MR, Mendelian randomization; HDL, high-density lipoprotein

Supplementary Table S7: Conditional F statistics for the multivariable MR analyses

**Analysis**/ trait	**Conditional F statistic**
**Accounting for alcohol consumtion**	
Educational attainment	54.05
Alcohol consumption	2.34
**Accounting for smoking initiation**	
Educational attainment	5.54
Smoking initiation	2.96
**Accounting for body mass index**	
Educational attainment	18.02
Body mass index	6.11
**Accounting for type 2 diabetes**	
Educational attainment	13.42
Type 2 diabetes	2.64
**Accounting for HDL cholesterol**	
Educational attainment	61.76
HDL cholesterol	1.63
**Accounting for systolic blood pressure**	
Educational attainment	34.47
Systolic blood pressure	4.56
**Accounting for all mediators**	
Educational attainment	3.41
Alcohol consumption	1.97
Smoking initiation	3.76
Body mass index	2.00
Type 2 diabetes	1.46
HDL cholesterol	4.04
Systolic blood pressure	1.72

HDL, high-density lipoprotein

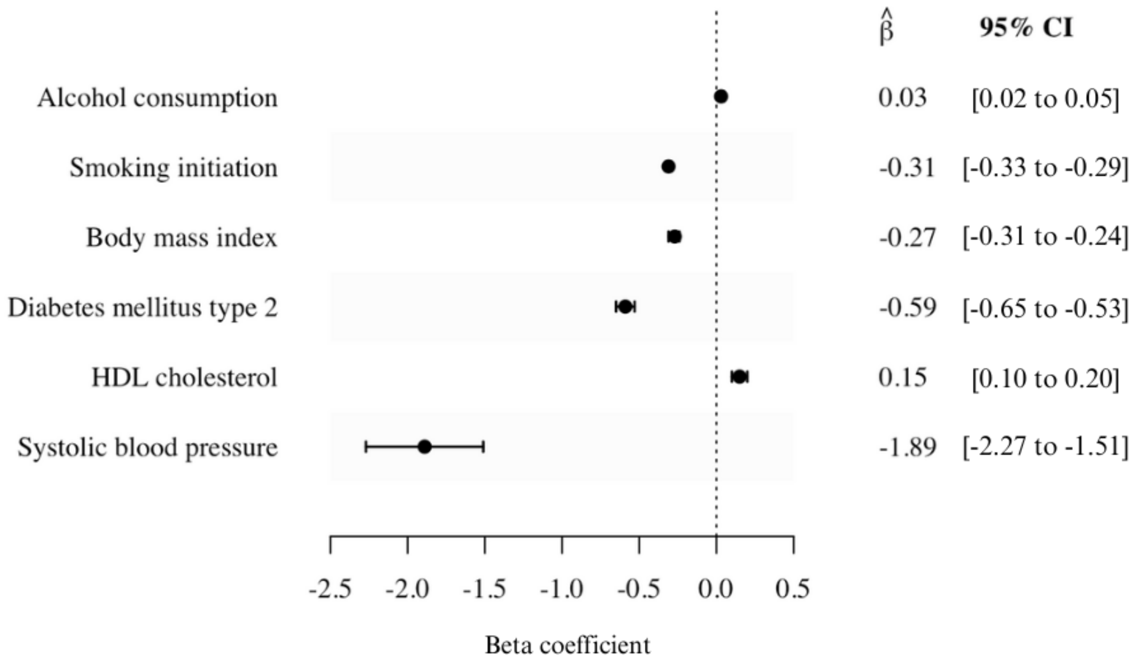

Supplementary Figure S1: Beta coefficients of modifiable mediating factors according to a standard deviation (SD) (3.4 years) increase in genetically predicted educational attainment. The mediators are in log(odds) units for smoking and diabetes type 2 and in SD units for alcohol consumption, body mass index, HDL cholesterol and systolic blood pressure

OR, odds ratio; CI, confidence interval; HDL, high-density lipoprotein

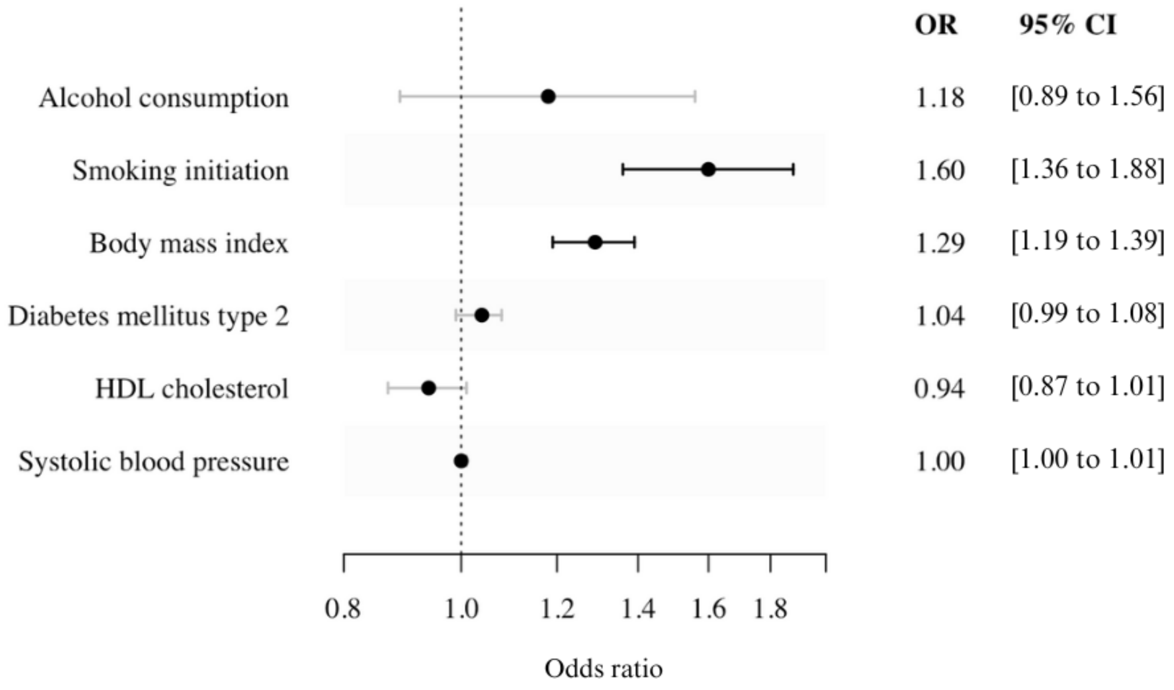

Supplementary Figure S2: Odds ratio (OR) of sepsis according to a log(odds) unit increase in genetically predicted smoking and type 2 diabetes, and a standard deviation increase in genetically predicted alcohol consumption, body mass index, HDL cholesterol and systolic blood pressure

OR, odds ratio; CI, confidence interval; HDL, high-density lipoprotein

Supplementary List S1: Single nucleotide polymorphisms (SNPs) included in the main analysis

SNP	EA Edu	OA Edu	EA Sepsis	OA Sepsis	Beta Edu	Beta Sepsis	EAF Edu	EAF Sepsis	SE Sepsis	P-value Sepsis	Proxy SNP Sepsis	Proxy EA Sepsis	Proxy OA Sepsis	SE Edu	P-value Edu
rs10002235	T	G	T	G	0.00692638	0.0169018	0.4229	0.422774	0.0145489	0.24				0.00106167	6.853E-11
rs10006551	T	C	T	C	0.00797542	-0.001206	0.5439	0.538291	0.0142283	0.83				0.00105086	3.221E-14
rs10014934	T	C	T	C	0.00912604	-0.0131913	0.6251	0.631742	0.0148068	0.3				0.00108089	3.089E-17
rs10016110	A	G	A	G	0.00947231	0.00834623	0.3817	0.397579	0.014546	0.21				0.00107709	1.437E-18
rs10026104	A	G	A	G	-0.0061292	-0.0050629	0.5889	0.597619	0.014509	0.98				0.00106375	8.325E-09
rs1004787	A	G	A	G	-0.0087593	-0.0195534	0.5525	0.532939	0.0142869	0.12				0.00105262	8.7E-17
rs10050950	A	G	A	G	-0.007299	-0.0336849	0.3352	0.326635	0.0151615	0.0189998				0.0011089	4.636E-11
rs10055846	A	G	A	G	0.00908396	-0.0174829	0.8437	0.846772	0.0198494	0.32				0.00144173	2.964E-10
rs10063055	T	C	T	C	-0.0124918	0.0203739	0.2371	0.253334	0.0163065	0.0899995				0.00123047	3.257E-24
rs1006749	A	G	A	G	0.00629554	-0.0129812	0.5199	0.517135	0.0141841	0.38				0.00105023	2.044E-09
rs10074178	A	G	A	G	0.0071457	-0.001311	0.5169	0.50466	0.014226	0.83				0.00104724	8.89E-12
rs10076155	T	C	T	C	-0.0084854	-0.0095419	0.24	0.244573	0.0164977	0.58				0.00122534	4.354E-12
rs1007731	A	C	A	C	-0.0112959	-0.0106124	0.1187	0.119457	0.0221907	0.709999				0.00162501	3.616E-12
rs1008516	T	C	T	C	0.0080346	-0.0231508	0.2219	0.226199	0.0171226	0.25				0.0012603	1.83E-10
rs10099657	A	G	A	G	-0.008129	-0.0044321	0.654	0.650994	0.0149115	0.75				0.00110044	1.501E-13
rs10123201	T	G	T	G	0.00661486	-0.0017117	0.7395	0.727366	0.0159935	0.62				0.00119233	2.89E-08
rs10124571	T	C	T	C	0.00946096	0.00121928	0.4014	0.411863	0.0144335	0.92				0.00106761	7.852E-19
rs10131962	A	G	A	G	0.00698983	-0.0132285	0.5998	0.591275	0.0144366	0.2				0.00106809	5.99E-11
rs10139457	A	G	A	G	-0.0068115	0.0002561	0.4373	0.435094	0.0143066	0.97				0.00105483	1.063E-10
rs10139828	T	C	T	C	0.00988637	-0.0005838	0.8617	0.870633	0.0211746	0.97				0.00151573	6.929E-11
rs10142459	T	C	T	C	0.00621045	-0.0129743	0.546	0.555753	0.0143446	0.52				0.00105126	3.473E-09
rs10145520	T	G	T	G	-0.0125829	-0.0033169	0.8074	0.802138	0.0178401	0.94				0.0013269	2.467E-21
rs10145770	A	G	A	G	-0.0095995	0.0274443	0.2145	0.211002	0.0175175	0.13				0.00127475	5.063E-14
rs10151339	T	G	T	G	0.00950342	-0.0091153	0.3172	0.305856	0.0154387	0.4				0.00112434	2.859E-17
rs10153150	T	G	T	G	0.0104831	0.0445773	0.1136	0.121769	0.02174	0.025				0.00164917	2.061E-10
rs10155336	C	G	C	G	-0.0156989	-0.0065489	0.0416	0.037661	0.0373572	0.73				0.00262637	2.273E-09
rs10157166	T	C	T	C	-0.0076071	-0.0215482	0.4028	0.414372	0.0144032	0.18				0.00106686	1.001E-12
rs10167909	T	C	T	C	0.00896839	0.029137	0.2147	0.219258	0.0172413	0.051				0.00127468	1.984E-12
rs10173182	A	G	A	G	-0.0061155	-0.021118	0.3439	0.350441	0.0148942	0.17				0.00110247	2.911E-08
rs10177230	T	C	T	C	0.00638609	0.00084881	0.4924	0.500696	0.0142812	0.91				0.00104705	1.067E-09
rs10180845	T	C	T	C	-0.0095958	0.0111018	0.6343	0.634336	0.014841	0.49				0.00108643	1.023E-18
rs10183564	T	C	T	C	-0.0087402	0.0137595	0.1973	0.1965	0.0179122	0.36				0.00131484	2.982E-11
rs10186870	T	G	T	G	-0.0060226	-0.0003144	0.6013	0.601101	0.0147344	0.95				0.00107165	1.906E-08
rs10189857	A	G	A	G	0.0137772	0.00090831	0.5701	0.567981	0.014343	0.8				0.00105695	7.74E-39
rs10190799	A	T	A	T	-0.0072135	-0.0179441	0.6271	0.609003	0.0146088	0.13				0.00108429	2.876E-11
rs10193498	A	T	A	T	0.0112688	-0.0118597	0.7471	0.759653	0.0167442	0.44				0.00120378	7.89E-21
rs10206657	A	G	A	G	0.00759242	0.0348196	0.2528	0.259474	0.0173407	0.0239999				0.00120693	3.163E-10
rs10208819	T	C	T	C	-0.0093322	0.00742746	0.1082	0.112439	0.0224419	0.709999				0.00168448	3.018E-08
rs10214086	A	T	A	T	0.00945301	-0.006884	0.7487	0.758744	0.0166048	0.77				0.00120648	4.695E-15
rs10240905	T	C	T	C	-0.0107869	0.0119826	0.3661	0.370243	0.0147985	0.31				0.00108618	3.046E-23
rs10241183	T	C	T	C	-0.0101267	0.0289449	0.3563	0.357626	0.0148304	0.0759994				0.00109291	1.939E-20
rs10256396	A	C	A	C	0.00654276	0.0152145	0.3382	0.325267	0.0151451	0.38				0.00110635	3.344E-09
rs10259526	T	G	T	G	-0.0088896	0.00025529	0.6117	0.612064	0.0145641	0.92				0.00107489	1.342E-16
rs10283803	T	C	T	C	-0.0057542	-0.0095237	0.5177	0.527393	0.0142043	0.760001				0.0010473	3.914E-08
rs1039617	T	C	T	C	-0.0078473	-0.0346518	0.4177	0.422639	0.0144403	0.017				0.00106107	1.406E-13
rs10401329	A	G	A	G	0.0109411	-0.0154043	0.1205	0.111784	0.0226967	0.32				0.0016143	1.219E-11
rs10402747	T	C	T	C	0.00685839	0.00822103	0.5144	0.518117	0.0142498	0.36				0.00105012	6.536E-11
rs10415488	T	C	T	C	-0.0060192	-0.0122507	0.6119	0.60683	0.0147128	0.35				0.00107864	2.399E-08
rs10419571	T	C	T	C	-0.0085055	-0.0118656	0.6825	0.685529	0.015347	0.58				0.00112796	4.681E-14
rs1043595	A	G	A	G	0.0166252	-0.0041897	0.2735	0.282279	0.0158201	0.83				0.00117629	2.357E-45
rs10440955	T	C	T	C	0.00608339	0.0169796	0.4717	0.486178	0.014232	0.12				0.00104819	6.482E-09
rs10445864	A	G	A	G	-0.0080735	-0.0152135	0.6052	0.613272	0.0145946	0.47				0.00107079	4.699E-14

rs10481106	A	G	A	G	-0.0123656	-0.0151983	0.1566	0.152612	0.0198803	0.36				0.00143998	8.902E-18
rs10496091	A	G	A	G	-0.0130629	0.0159941	0.2803	0.28065	0.0158071	0.19				0.001165	3.532E-29
rs10496632	C	G	C	G	0.0111374	7.5796E-05	0.2838	0.273984	0.0159105	0.98				0.00116062	8.3E-22
rs10499146	A	G	A	G	0.0108069	-0.0169865	0.1332	0.132238	0.0209645	0.75				0.00154007	2.257E-12
rs10511704	A	T	A	T	-0.0073595	0.023902	0.312	0.312337	0.0154085	0.0669993				0.00112953	7.235E-11
rs10514891	A	G	A	G	0.0136571	0.00631086	0.8832	0.877832	0.0216402	0.98				0.00162936	5.2E-17
rs10521132	A	G	A	G	-0.0083613	-0.0074442	0.25	0.255501	0.0163088	0.64	rs12927553	A	T	0.00120972	4.79E-12
rs1053951	A	G	A	G	-0.0066007	-0.0231843	0.5723	0.572828	0.014339	0.0680002				0.00105762	4.341E-10
rs1054378	T	C	T	C	0.00631871	-0.0061982	0.4233	0.459713	0.0142484	0.33	rs3809811	T	C	0.00106054	2.555E-09
rs1056010	T	C	T	C	-0.0099782	0.00933809	0.6058	0.589986	0.0144188	0.85				0.00107133	1.229E-20
rs1058790	A	G	A	G	-0.0139612	0.00072335	0.8205	0.830101	0.0189962	0.74				0.00136396	1.373E-24
rs10732635	T	G	T	G	0.00697261	-0.0168643	0.5597	0.554717	0.0142603	0.19				0.00105434	3.766E-11
rs10734924	T	G	T	G	-0.0117612	0.0333733	0.2622	0.262758	0.0161465	0.0259998				0.00118967	4.798E-23
rs10741228	T	C	T	C	-0.0064378	0.0119027	0.4316	0.430205	0.0144451	0.28				0.00106187	1.339E-09
rs10743083	A	G	A	G	0.00815668	0.010547	0.1775	0.175829	0.018628	0.56				0.00137817	3.255E-09
rs10745307	A	G	A	G	-0.0106863	0.0257594	0.5474	0.586874	0.0144313	0.0949992	rs666842	T	C	0.00105134	2.849E-24
rs10752160	T	C	T	C	-0.0075634	0.018931	0.2965	0.298063	0.0155444	0.26				0.00114569	4.073E-11
rs10752262	T	C	T	C	0.00931827	-0.0117936	0.4177	0.4286	0.0143595	0.4				0.00106464	2.081E-18
rs10762069	C	G	C	G	0.0104999	-0.0075245	0.6818	0.676833	0.0151536	0.649999				0.00112425	9.695E-21
rs10762188	A	G	A	G	-0.0059479	-0.013705	0.3643	0.37181	0.0147475	0.27				0.00108777	4.552E-08
rs10762199	T	C	T	C	0.00894654	0.0148573	0.7649	0.768959	0.0168468	0.4				0.00123457	4.263E-13
rs10763979	A	G	A	G	0.00920287	0.0174729	0.6215	0.623722	0.0146839	0.35				0.00107899	1.475E-17
rs10764493	A	G	A	G	0.0131507	-0.0183542	0.8892	0.886095	0.0224312	0.38				0.00166703	3.058E-15
rs10765789	A	G	A	G	0.0107781	0.0233523	0.3827	0.37719	0.0146191	0.16				0.00107655	1.349E-23
rs10771003	T	G	T	G	0.00785219	0.00715809	0.7773	0.777776	0.017143	0.7				0.001258	4.316E-10
rs10772644	C	G	C	G	0.013968	-0.02732	0.8916	0.892555	0.0230197	0.22				0.00168311	1.052E-16
rs10786066	T	C	T	C	0.00741047	-0.0320344	0.7166	0.721977	0.015865	0.0580003				0.00116367	1.911E-10
rs10791303	T	C	T	C	0.00875828	-0.0135457	0.6202	0.622575	0.0147493	0.38				0.00107843	4.603E-16
rs10805248	A	G	A	G	-0.0116772	0.0210971	0.825	0.823708	0.0186693	0.33				0.0013771	2.261E-17
rs10815961	T	C	T	C	0.00634964	-0.0079841	0.497	0.50576	0.0142644	0.649999				0.00104653	1.299E-09
rs10818407	T	C	T	C	0.00791205	0.0244756	0.2278	0.232378	0.0168459	0.0949992				0.00124775	2.281E-10
rs10819272	T	C	T	C	0.00697021	-0.0049002	0.5954	0.575977	0.0143475	0.43				0.00106623	6.267E-11
rs10819974	T	C	T	C	-0.0081466	0.00332592	0.2356	0.224436	0.0170406	0.91				0.00123317	3.952E-11
rs10821992	A	T	A	T	-0.0079509	-0.0265309	0.3412	0.342459	0.0149602	0.0530005				0.00110379	5.862E-13
rs10822745	T	C	T	C	0.0101244	0.0154421	0.5769	0.56481	0.0143391	0.47				0.00105925	1.201E-21
rs10830858	T	C	T	C	0.00819375	-0.0181844	0.5062	0.506344	0.0142017	0.17				0.00104659	4.926E-15
rs10842296	T	C	T	C	0.00736597	-0.0416029	0.2448	0.237851	0.0166832	0.00819993				0.00121761	1.454E-09
rs10853981	A	G	A	G	-0.0086839	0.0081265	0.3286	0.330087	0.0151022	0.62				0.00111461	6.642E-15
rs10854143	T	C	T	C	0.00658387	0.0392647	0.4853	0.487886	0.0141794	0.00179999				0.00104868	3.417E-10
rs10858051	A	G	A	G	0.00778811	-0.0079613	0.7446	0.749464	0.0163597	0.59				0.00119989	8.55E-11
rs10859638	A	G	A	G	-0.0074703	-0.013951	0.2427	0.231512	0.0168056	0.23				0.00122062	9.343E-10
rs10860215	T	C	T	C	-0.0091077	-0.026814	0.134	0.131468	0.0209986	0.3				0.00153603	3.036E-09
rs10860219	A	G	A	G	0.00859036	0.00932872	0.8352	0.840768	0.019442	0.57				0.00141079	1.135E-09
rs10862376	A	T	A	T	0.0134376	-0.0097257	0.1473	0.14429	0.0202217	0.68				0.00147643	8.893E-20
rs10865834	A	G	A	G	-0.0104895	-0.0033425	0.2525	0.228643	0.0169143	0.52	rs9846736	A	G	0.00120691	3.584E-18
rs10867226	T	C	T	C	0.00673134	0.0180589	0.2594	0.261352	0.0161679	0.25				0.00119397	1.722E-08
rs10874759	A	G	A	G	0.0128936	0.00125381	0.655	0.649723	0.0148517	0.89				0.00110073	1.082E-31
rs10880961	C	G	C	G	0.00830363	-0.0239489	0.398	0.393473	0.0145543	0.0769999				0.00106899	8.01E-15
rs10885931	A	G	A	G	0.00701558	-0.0131327	0.6583	0.671006	0.0151166	0.42	rs10886001	T	C	0.0011034	2.044E-10
rs10886010	A	G	A	G	-0.0086991	-0.0009226	0.42	0.409283	0.0145455	0.74				0.0010603	2.316E-16
rs10887465	A	C	A	C	0.0127558	-0.0127191	0.2658	0.272256	0.0159504	0.57				0.00118463	4.909E-27
rs10890995	A	G	A	G	-0.0092015	0.0116473	0.2999	0.294873	0.0155967	0.53				0.00114194	7.769E-16
rs10896636	C	G	C	G	0.0093576	-0.0164237	0.6588	0.65554	0.0149953	0.15				0.00110365	2.273E-17
rs10897561	T	C	T	C	0.00784455	-0.0259893	0.6491	0.64488	0.0149562	0.0359998				0.0010967	8.487E-13
rs10903706	T	C	T	C	0.00605229	0.02724	0.6665	0.663282	0.0150126	0.0959997				0.00110985	4.95E-08
rs10913420	T	C	T	C	0.00933451	0.0178721	0.1726	0.177251	0.0187123	0.38				0.00138646	1.664E-11

rs10916679	A	C	A	C	0.00714966	-0.022429	0.2682	0.25694	0.016218	0.1				0.0011811	1.418E-09
rs10920336	A	G	A	G	0.00622417	-0.0116823	0.5208	0.520655	0.0142271	0.39				0.00105179	3.263E-09
rs10929474	A	T	A	T	0.0114465	-0.0038848	0.2135	0.205111	0.0176029	0.77				0.00128469	5.116E-19
rs10942058	T	C	T	C	0.00660854	-0.0133296	0.5029	0.493043	0.0142276	0.41	rs10942064	C	T	0.00104666	2.724E-10
rs10946469	A	C	A	C	-0.0061825	0.0239411	0.6378	0.63739	0.014741	0.0690001				0.00109092	1.451E-08
rs10948447	A	G	A	G	-0.0063643	0.010638	0.5277	0.522708	0.0142616	0.39				0.00104841	1.275E-09
rs10970007	T	C	T	C	-0.0081255	0.0120531	0.6773	0.673555	0.0153511	0.42	rs4574901	T	C	0.00111924	3.869E-13
rs10983486	A	C	A	C	0.00703793	0.00387305	0.5758	0.580548	0.0143953	0.86	rs10983487	C	T	0.00105888	2.993E-11
rs10984444	A	C	A	C	-0.0116381	0.0104029	0.5076	0.525179	0.0142156	0.27				0.00104677	1.02E-28
rs10984573	A	T	A	T	-0.00747	-0.0116083	0.7147	0.727294	0.015943	0.62				0.0011596	1.183E-10
rs10992836	T	C	T	C	-0.0116387	-0.0171732	0.3407	0.335608	0.0150813	0.18				0.00110418	5.62E-26
rs10994470	A	G	A	G	0.0199637	0.0578909	0.039	0.044141	0.0354407	0.0649995				0.00271966	2.124E-13
rs10995839	A	C	A	C	-0.0084191	-0.0010498	0.8424	0.843461	0.0197832	0.96				0.00144178	5.238E-09
rs11001967	A	G	A	G	-0.0062053	0.0291741	0.4364	0.436651	0.0144229	0.0379997				0.00105551	4.129E-09
rs11011859	A	G	A	G	0.0112915	-0.016756	0.8828	0.885452	0.0222818	0.43				0.00162674	3.879E-12
rs11011932	T	C	T	C	0.00858474	0.0224518	0.2329	0.226014	0.016985	0.26				0.00124136	4.661E-12
rs11019128	T	C	T	C	-0.0087051	0.014103	0.3824	0.393576	0.0145104	0.32				0.00107702	6.346E-16
rs11020370	T	C	T	C	-0.0079869	0.0161322	0.8226	0.834643	0.0191335	0.35				0.00137014	5.567E-09
rs11024359	A	G	A	G	-0.0067177	-0.0065321	0.6014	0.594199	0.0144663	0.7				0.00115594	6.193E-09
rs11028323	A	C	A	C	-0.0098407	-0.0111786	0.5149	0.51062	0.0141842	0.37				0.00104835	6.2E-21
rs11056726	A	T	A	T	-0.0069045	-0.0115269	0.2337	0.228578	0.0169182	0.41				0.00123658	2.356E-08
rs1107871	A	G	A	G	-0.0085544	0.011115	0.552	0.549491	0.0143002	0.66				0.00105221	4.286E-16
rs11081529	T	C	T	C	0.0122793	-0.005015	0.7213	0.707074	0.0156189	0.6				0.00116737	7.097E-26
rs1109156	T	C	T	C	0.00822664	-0.0047051	0.208	0.20646	0.0176178	0.8				0.0012901	1.813E-10
rs11111443	A	G	A	G	0.00722739	-0.0191069	0.6334	0.636698	0.0147445	0.19				0.00108587	2.813E-11
rs11122148	A	C	A	C	0.0101634	0.0249121	0.1561	0.157067	0.0195152	0.15				0.00144167	1.791E-12
rs11131601	T	G	T	G	0.00925245	-0.0258802	0.4097	0.409267	0.0144516	0.0430002				0.001064	3.449E-18
rs11138947	T	C	T	C	0.00957707	0.00899475	0.7208	0.722897	0.015876	0.47				0.00116655	2.22E-16
rs11143270	T	G	T	G	-0.0079139	0.0254541	0.1578	0.16118	0.0192846	0.2				0.00143552	3.526E-08
rs1114504	T	C	T	C	-0.0088889	-0.0442606	0.825	0.832125	0.0190268	0.0189998				0.00137838	1.129E-10
rs111493473	A	G	A	G	0.0151725	-0.0366563	0.8025	0.803698	0.0178586	0.0449997				0.00131434	7.895E-31
rs11154134	A	G	A	G	-0.0072025	-0.0191524	0.6906	0.685433	0.0152666	0.25				0.00113198	1.982E-10
rs11157931	A	C	A	C	-0.0115194	-0.0076357	0.3923	0.395606	0.0145296	0.69				0.00107166	5.976E-27
rs11158200	A	G	A	G	0.00860686	-0.0071143	0.5367	0.541842	0.0142788	0.74				0.00105036	2.528E-16
rs11159067	A	G	A	G	0.00947897	-0.0140805	0.6722	0.67682	0.0151682	0.46				0.0011147	1.836E-17
rs11163064	C	G	C	G	-0.0078361	0.0164535	0.7894	0.787319	0.0173405	0.450001				0.00128332	1.019E-09
rs111660908	A	G	A	G	0.0084094	0.0458846	0.1446	0.151383	0.0198548	0.00519996				0.00148793	1.587E-08
rs11171681	A	C	A	C	0.0164434	0.0636251	0.9578	0.961317	0.0368118	0.0359998				0.0026043	2.715E-10
rs11172371	T	C	T	C	-0.0106973	-0.03438	0.3227	0.340938	0.0150767	0.02				0.00111955	1.233E-21
rs1117310	A	G	A	G	0.00741494	0.00476064	0.4616	0.460672	0.0142367	0.709999				0.00104961	1.612E-12
rs11179786	T	C	T	C	-0.0074145	-0.0155689	0.5842	0.588773	0.0144221	0.34				0.00106167	2.868E-12
rs11180882	T	C	T	C	-0.0075788	0.0171845	0.7638	0.753962	0.0164456	0.67				0.00123241	7.762E-10
rs111821073	T	C	T	C	0.0153285	0.0109064	0.1544	0.159213	0.0194138	0.709999	rs12375999	T	C	0.0014519	4.701E-26
rs11191193	A	G	A	G	0.0162974	-0.0012051	0.6552	0.648955	0.0149102	0.98				0.00110103	1.422E-49
rs1119258	T	G	T	G	-0.0072312	0.0261887	0.3784	0.387937	0.0145775	0.0490004				0.0010789	2.049E-11
rs11196397	A	C	A	C	0.00897314	-0.0275255	0.2731	0.276765	0.0159348	0.0870001				0.00117488	2.218E-14
rs11213482	A	G	A	G	-0.0079338	0.0118138	0.8405	0.828159	0.0187735	0.68				0.00143488	3.217E-08
rs11218422	T	C	T	C	0.00879043	-0.002926	0.5793	0.585843	0.0144264	0.87				0.00105992	1.1E-16
rs112209391	T	C	T	C	0.0139093	0.0095622	0.1044	0.107528	0.0228852	0.56				0.00171122	4.343E-16
rs112255786	C	G	C	G	-0.0169754	0.0257958	0.9385	0.936323	0.0290915	0.33				0.00217819	6.514E-15
rs11232546	T	C	T	C	0.00632529	-0.0094803	0.6582	0.669797	0.0150942	0.709999				0.00110328	9.863E-09
rs11243852	T	C	T	C	0.0122412	0.0292196	0.2381	0.236618	0.0167076	0.0700003				0.00122868	2.212E-23
rs11245450	A	G	A	G	0.00685839	-0.0349674	0.4236	0.421938	0.0145076	0.0179999				0.00106185	1.057E-10
rs11252031	A	G	A	G	-0.0063575	-0.0036332	0.3066	0.312366	0.0153153	0.81				0.00113484	2.122E-08
rs112579674	C	G	C	G	0.00925701	0.00485601	0.8177	0.819734	0.0184715	0.94				0.00135544	8.539E-12
rs1125854	T	C	T	C	-0.0077335	0.0237341	0.3726	0.371285	0.0146952	0.1				0.00108223	8.957E-13

rs11264531	T	C	T	C	0.00688273	-0.0001212	0.361	0.35622	0.0149589	0.74				0.00109237	2.958E-10
rs112650421	T	G	T	G	-0.0205678	0.0327795	0.0236	0.023635	0.0468909	0.53				0.00345797	2.721E-09
rs112706692	T	C	T	C	0.0214383	-0.0638232	0.0238	0.023283	0.0480539	0.22				0.00356528	1.824E-09
rs1128687	T	C	T	C	-0.0081293	-0.0009383	0.5708	0.560904	0.0142997	0.85				0.00106019	1.752E-14
rs1128956	T	G	T	G	-0.01024	-0.0066326	0.8262	0.821942	0.0185395	0.87				0.00138635	1.511E-13
rs113254113	T	C	T	C	-0.0131433	-0.0235425	0.1126	0.114821	0.0222482	0.32				0.00166888	3.387E-15
rs113746525	T	G	T	G	0.0299906	-0.0765027	0.0233	0.024435	0.0469366	0.13				0.00347453	6.07E-18
rs113801542	A	G	A	G	-0.0375136	0.017353	0.9872	0.98491	0.0591466	0.780001				0.00468989	1.259E-15
rs113831544	A	T	A	T	-0.0143825	-0.0118163	0.0522	0.052221	0.0319558	0.48				0.00235244	9.746E-10
rs114055010	C	G	C	G	-0.0320754	-0.0301195	0.9852	0.987465	0.0672977	0.73				0.00441722	3.84E-13
rs114221183	A	G	A	G	0.00827272	-0.0004271	0.1412	0.138336	0.0207412	0.9	rs17013497	T	C	0.00150271	3.688E-08
rs114340522	C	G	C	G	0.0144511	-0.0109483	0.0528	0.049088	0.0331374	0.649999				0.00234906	7.671E-10
rs114468556	A	T	A	T	0.0294941	-0.0337207	0.0427	0.046427	0.0342307	0.38				0.00260857	1.22E-29
rs114590673	A	T	A	T	-0.0294775	0.0222621	0.0142	0.013315	0.0626048	0.74				0.00446522	4.064E-11
rs11505217	A	C	A	C	0.00949123	-0.0107852	0.8389	0.846681	0.0196929	0.719999				0.00142334	2.585E-11
rs115124024	A	C	A	C	0.0178321	-0.0077323	0.9505	0.94863	0.0322209	0.77				0.00241295	1.465E-13
rs1151282	A	C	A	C	0.0070194	0.010117	0.282	0.286081	0.0156851	0.6				0.00116301	1.587E-09
rs115330285	A	G	A	G	0.00740769	0.0063854	0.2011	0.196674	0.0179694	0.84				0.00131339	1.703E-08
rs115420676	T	C	T	C	-0.0201491	-0.0813517	0.0258	0.027504	0.0438372	0.0719996				0.00366983	4.003E-08
rs115438240	T	G	T	G	-0.0187073	-0.0136655	0.941	0.945251	0.0320731	0.719999				0.00222222	3.813E-17
rs1154780	A	G	A	G	0.00842432	0.00932457	0.5444	0.546316	0.014422	0.64				0.00105095	1.091E-15
rs11562848	T	C	T	C	0.0112585	0.0686643	0.0947	0.09171	0.024638	0.00549997				0.00178707	2.979E-10
rs11571404	T	C	T	C	0.00875024	0.011265	0.1971	0.202739	0.0177094	0.37				0.00131897	3.268E-11
rs11583404	T	C	T	C	-0.0062937	-0.0119345	0.6373	0.632408	0.0147077	0.4				0.00108834	7.36E-09
rs11589723	T	C	T	C	-0.0127441	0.0421877	0.8976	0.90248	0.0239927	0.0759994				0.00172592	1.537E-13
rs11595166	T	C	T	C	0.0132371	-0.0256931	0.0813	0.084416	0.0256791	0.39				0.00192099	5.536E-12
rs115994176	T	C	T	C	-0.0109417	-0.0029248	0.6175	0.599678	0.0144767	0.93	rs7033596	A	G	0.00124483	1.498E-18
rs11599424	A	T	A	T	0.0104771	-0.0100784	0.3793	0.389372	0.0147233	0.5				0.00108558	4.856E-22
rs11607466	T	C	T	C	-0.0116861	0.00189345	0.1032	0.106996	0.0229806	0.92				0.00172009	1.091E-11
rs11614957	A	G	A	G	0.0120583	-0.0182905	0.4423	0.444369	0.0142993	0.27				0.00105455	2.822E-30
rs116173579	T	C	T	C	-0.0164886	-0.0220715	0.0331	0.03718	0.0375776	0.7				0.00292547	1.735E-08
rs116193437	A	C	A	C	0.0190304	0.025655	0.0258	0.023469	0.0486822	0.88				0.00340596	2.308E-08
rs11623285	T	G	T	G	-0.0129564	-0.0431776	0.8654	0.860409	0.020582	0.0109999				0.00153993	3.974E-17
rs11633934	A	C	A	C	0.00803016	0.00096948	0.5433	0.539389	0.0143286	0.56				0.00105045	2.102E-14
rs11643516	T	C	T	C	-0.0081182	-0.0206265	0.4128	0.407382	0.0144376	0.16				0.00106293	2.217E-14
rs11645537	A	G	A	G	0.00963241	-0.0317866	0.1458	0.13724	0.0208796	0.11				0.0014835	8.396E-11
rs116547571	A	G	A	G	-0.026437	-0.0095083	0.0183	0.019045	0.0519147	0.82				0.00394853	2.154E-11
rs11657342	A	G	A	G	0.0145941	-0.0177239	0.3671	0.369256	0.0147099	0.0969996				0.00234394	4.787E-10
rs11657896	T	G	T	G	0.00633053	0.0134112	0.3778	0.379247	0.0146294	0.36				0.00107924	4.466E-09
rs11664320	T	C	T	C	0.0175932	-0.0084542	0.5649	0.576449	0.0143628	0.75				0.00105544	2.21E-62
rs11667653	T	C	T	C	-0.0086372	0.0102112	0.1897	0.189257	0.0184963	0.66				0.00133886	1.109E-10
rs11672103	T	C	T	C	-0.0076066	-0.0143672	0.5447	0.55342	0.0143586	0.34				0.00105101	4.57E-13
rs116762274	C	G	C	G	0.0213959	-0.0787764	0.019	0.024066	0.0479797	0.0819993				0.00391738	4.715E-08
rs1167796	A	G	A	G	0.0120302	-0.0092911	0.4253	0.434802	0.0143087	0.49				0.00105963	7.132E-30
rs1168114	A	G	A	G	-0.007286	-0.0098057	0.3306	0.347653	0.0149131	0.55				0.00111229	5.728E-11
rs11681881	A	G	A	G	-0.010292	0.0284967	0.8651	0.87114	0.0214586	0.13				0.00154576	2.776E-11
rs11686212	A	G	A	G	-0.0076582	-0.0079063	0.6288	0.630735	0.0146937	0.66				0.0010838	1.594E-12
rs11690035	T	C	T	C	-0.019325	3.1456E-05	0.0742	0.07068	0.0277941	0.82				0.00199642	3.664E-22
rs11692364	A	G	A	G	-0.005855	0.00426601	0.4843	0.484805	0.0142384	0.69				0.00104702	2.249E-08
rs116973	T	C	T	C	-0.0067846	0.0069316	0.5356	0.529983	0.0141983	0.74				0.00104917	1.001E-10
rs11698330	T	G	T	G	0.0101121	0.0151619	0.2011	0.203815	0.0176525	0.22				0.00130545	9.484E-15
rs117005905	T	C	T	C	0.0155759	-0.0140173	0.119	0.110858	0.0229602	0.35				0.00161649	5.633E-22
rs11703948	A	G	A	G	-0.0145783	0.0325498	0.9047	0.898759	0.0235013	0.1				0.00178331	2.967E-16
rs11706224	T	C	T	C	0.00686095	-0.0121518	0.6442	0.646534	0.0149154	0.3				0.00109295	3.442E-10
rs117070316	T	G	T	G	0.0350656	0.0585971	0.032	0.034602	0.0388341	0.0619998				0.00342604	1.386E-24
rs11708735	T	C	T	C	0.0069326	0.012214	0.2731	0.282291	0.0160706	0.37				0.0011752	3.652E-09

rs11709621	T	C	T	C	-0.0095736	-0.0082162	0.2031	0.204666	0.0177416	0.48				0.00130063	1.827E-13
rs1171150	T	C	T	C	0.00920477	-0.0135425	0.3735	0.366579	0.0147327	0.33				0.00108394	2.033E-17
rs11724690	T	G	T	G	0.00949049	-0.0185336	0.2939	0.284822	0.0158379	0.19				0.00114895	1.454E-16
rs11731597	T	C	T	C	0.00844621	-0.0220597	0.2947	0.287197	0.015707	0.12				0.00114772	1.846E-13
rs117349657	A	G	A	G	-0.0208044	-0.0700268	0.9783	0.976122	0.0464144	0.0759994				0.00366546	1.381E-08
rs11737459	A	T	A	T	0.0107297	-0.0276813	0.6958	0.697243	0.015462	0.1				0.00113734	3.943E-21
rs11741009	T	C	T	C	-0.0071912	-0.0023922	0.2225	0.231823	0.0170555	0.94				0.00125857	1.107E-08
rs117416770	T	C	T	C	-0.0182963	-0.0073743	0.0347	0.034843	0.0391168	0.88				0.00287167	1.875E-10
rs11746390	T	C	T	C	-0.0085993	0.0317045	0.7315	0.735906	0.0161134	0.0490004				0.00118084	3.279E-13
rs117520198	A	G	A	G	0.0106951	0.0151757	0.1911	0.193821	0.0179897	0.54				0.00133269	1.012E-15
rs117520996	T	G	T	G	0.0219636	-0.0286738	0.049	0.047653	0.0333302	0.21				0.00249812	1.465E-18
rs11752914	T	C	T	C	0.00796861	-0.0219166	0.8043	0.804148	0.01791	0.15				0.00131926	1.54E-09
rs11755241	T	C	T	C	-0.011565	0.0126517	0.1923	0.191166	0.0180368	0.46	rs4947086	T	C	0.00133985	6.058E-18
rs11755280	T	C	T	C	-0.007118	-0.0026212	0.7393	0.733593	0.0161638	0.73				0.00119221	2.361E-09
rs117568184	T	C	T	C	0.0184479	-0.115351	0.0259	0.027817	0.0432698	0.0280001				0.00334628	3.522E-08
rs117588584	T	C	T	C	-0.0132326	-0.0094536	0.0917	0.09476	0.0242227	0.69				0.00181379	2.982E-13
rs117623407	A	G	A	G	-0.011147	0.0150319	0.8515	0.85277	0.0200194	0.31				0.0014804	5.075E-14
rs11766968	T	C	T	C	-0.00973	-0.0019377	0.1433	0.146878	0.0201952	0.89				0.00150301	9.55E-11
rs11768912	T	C	T	C	0.00931177	0.00058543	0.8749	0.867304	0.0209034	0.96				0.00158163	3.919E-09
rs11772232	T	C	T	C	0.019178	-0.0065523	0.1681	0.166032	0.019104	0.62	rs11764779	G	A	0.00140636	2.436E-42
rs117736305	A	G	A	G	-0.0169635	0.0245535	0.0556	0.058206	0.0302867	0.43				0.00229013	1.288E-13
rs11773992	C	G	C	G	-0.0138016	0.00217559	0.1783	0.175873	0.0187911	0.84				0.00136722	5.842E-24
rs11774212	T	C	T	C	0.0156703	-0.0019364	0.5107	0.515782	0.0142447	0.97				0.00104855	1.695E-50
rs11775108	A	G	A	G	-0.0093447	0.00105573	0.4871	0.480797	0.0142161	0.82				0.0010478	4.734E-19
rs117755721	A	G	A	G	0.0193993	-0.0101398	0.0268	0.028259	0.0428083	0.84				0.00329631	3.985E-09
rs117769860	T	C	T	C	0.0275366	0.012369	0.9859	0.983164	0.055949	0.83				0.00447671	7.686E-10
rs11777093	T	C	T	C	0.0067712	-0.00729	0.483	0.489052	0.014258	0.630001				0.00104734	1.013E-10
rs11780633	T	C	T	C	-0.0143116	-0.0195849	0.1408	0.149234	0.0199555	0.31				0.00150502	1.925E-21
rs11783263	C	G	C	G	0.00726173	-0.0134266	0.6471	0.641644	0.0149204	0.31				0.00109511	3.328E-11
rs117849041	T	C	T	C	0.0116296	0.0422194	0.1052	0.111274	0.0228462	0.0899995				0.00171491	1.187E-11
rs11786807	A	G	A	G	-0.0081145	-0.0166025	0.5729	0.573782	0.0143425	0.13				0.00105795	1.724E-14
rs11789013	T	C	T	C	0.0116659	0.0105393	0.7554	0.758835	0.0166615	0.38				0.00122032	1.183E-21
rs118004210	A	G	A	G	0.0178388	-0.0122677	0.0271	0.030208	0.0414999	0.83				0.00322402	3.149E-08
rs118057662	T	G	T	G	0.0153545	0.00556064	0.9556	0.955829	0.0346471	0.86				0.00254336	1.569E-09
rs118084175	T	C	T	C	-0.0163997	-0.0042525	0.0397	0.03721	0.0376807	0.97				0.00268102	9.544E-10
rs118134876	T	C	T	C	-0.0310947	-0.0007686	0.0611	0.059923	0.0299183	0.77				0.00218685	6.973E-46
rs1182532	A	C	A	C	0.0110653	-0.0165768	0.1549	0.145814	0.02128	0.44				0.00146338	3.997E-14
rs11841508	T	C	T	C	0.00703346	-0.0055911	0.3006	0.306128	0.0153905	0.95				0.00114143	7.184E-10
rs11857810	A	G	A	G	0.00757805	-0.0093741	0.3924	0.392664	0.0145535	0.38				0.00107176	1.541E-12
rs11861256	A	C	A	C	0.0100396	-0.0063159	0.191	0.187136	0.0182636	0.55				0.00133168	4.733E-14
rs11870612	A	G	A	G	-0.0096183	-0.0187026	0.1024	0.099512	0.0237159	0.42				0.00174247	3.395E-08
rs11871722	T	C	T	C	0.00711884	-0.0335479	0.2626	0.261848	0.0161537	0.02				0.00119189	2.333E-09
rs11877152	T	C	T	C	-0.0142529	0.00282305	0.8905	0.886865	0.0223791	0.97				0.00167671	1.892E-17
rs11886336	A	G	A	G	0.00862355	-0.0194127	0.5781	0.571858	0.0143403	0.21				0.00105951	3.983E-16
rs11897647	T	C	T	C	0.00919216	0.00993948	0.1811	0.166162	0.0190511	0.86				0.00135874	1.333E-11
rs11915916	T	C	T	C	0.00959412	-0.0117049	0.1368	0.14337	0.0203234	0.73	rs2215708	A	G	0.0015227	2.959E-10
rs11921658	T	C	T	C	-0.0074234	0.0428759	0.8065	0.807545	0.0183237	0.02				0.00132547	2.138E-08
rs11925699	A	G	A	G	-0.007664	-0.0289564	0.4667	0.465158	0.0142465	0.0359998				0.00104884	2.733E-13
rs11932176	T	G	T	G	0.00605335	-0.0063873	0.4173	0.446617	0.0147302	0.5	rs13139176	A	G	0.00107077	1.575E-08
rs1193240	A	C	A	C	0.00846827	-0.0140422	0.4107	0.43858	0.0143285	0.39	rs1193238	T	C	0.00106395	1.73E-15
rs11937610	A	G	A	G	0.00622419	-0.0097387	0.6519	0.653771	0.0149274	0.4				0.00109842	1.459E-08
rs11942352	A	G	A	G	-0.0089357	0.0235217	0.8053	0.799525	0.0177351	0.19				0.00132235	1.401E-11
rs11982791	A	C	A	C	-0.0204716	-0.0450704	0.0528	0.054336	0.0312775	0.12				0.00233999	2.158E-18
rs11990191	A	G	A	G	0.00577143	0.0205714	0.5275	0.529477	0.0142791	0.18	rs62527281	T	C	0.00104925	3.781E-08
rs12028526	T	G	T	G	0.0071093	-0.0220711	0.5576	0.563031	0.0143368	0.16				0.00105352	1.501E-11
rs12034740	A	C	A	C	0.00711399	-0.0015575	0.4987	0.504644	0.0142464	0.98				0.00104831	1.151E-11

rs12038928	A	G	A	G	-0.0059064	0.00851967	0.6074	0.599533	0.0145471	0.74	0.00107182	3.581E-08
rs12045428	A	C	A	C	-0.0105793	-0.0274231	0.1881	0.185924	0.0182603	0.1	0.00133896	2.764E-15
rs12050945	C	G	C	G	-0.0083295	0.00627288	0.7575	0.770137	0.0168498	0.49	0.00122102	8.992E-12
rs12054166	C	G	C	G	0.00730381	-0.0118188	0.7352	0.740889	0.0162635	0.42	0.00118591	7.324E-10
rs12055782	A	G	A	G	-0.0117761	-0.0301455	0.7179	0.717629	0.0158021	0.0539995	0.00116273	4.154E-24
rs1206133	T	C	T	C	0.00759357	0.00097762	0.7524	0.754224	0.0166951	0.87	0.00121265	3.804E-10
rs12064553	A	T	A	T	0.0092982	0.0132385	0.2278	0.228117	0.0169444	0.53	0.00124759	9.137E-14
rs12065238	A	G	A	G	0.0100911	0.0115077	0.1696	0.168856	0.0189718	0.48	0.0013943	4.57E-13
rs12071309	T	C	T	C	-0.0061738	0.00405725	0.6804	0.680551	0.0152826	0.99	0.00112272	3.827E-08
rs12078410	T	C	T	C	0.00630185	-0.0116035	0.4731	0.476306	0.0142053	0.48	0.00104803	1.818E-09
rs1207953	C	G	C	G	-0.0079709	-0.0131407	0.619	0.612686	0.0149184	0.36	0.00108073	1.643E-13
rs12089815	A	G	A	G	0.0185277	-0.0082209	0.5396	0.548621	0.0142996	0.58	0.0010501	1.141E-69
rs12107377	A	G	A	G	0.0063775	-0.0129986	0.5155	0.50513	0.0143034	0.34	0.0010471	1.123E-09
rs12113634	T	C	T	C	0.00907811	-0.0182045	0.6426	0.645115	0.0148508	0.2	0.00109185	9.23E-17
rs12124493	A	G	A	G	-0.0117305	-0.01184	0.313	0.326014	0.0151306	0.5	0.00113129	3.425E-25
rs12126231	A	G	A	G	0.0117433	-0.0228403	0.6159	0.601637	0.0144859	0.0810009	0.00107581	9.701E-28
rs12131823	A	G	A	G	0.0103049	0.0004749	0.5374	0.525531	0.0142093	0.93	0.00105201	1.182E-22
rs12134760	A	C	A	C	0.0127757	0.0111018	0.1392	0.141392	0.0204065	0.46	0.00151204	2.928E-17
rs12142143	T	C	T	C	0.00601695	-0.0069114	0.4715	0.46527	0.0142906	0.42	0.00104851	9.566E-09
rs12151015	C	G	C	G	-0.0095597	-0.0248397	0.8835	0.885065	0.0224439	0.29	0.00164016	5.603E-09
rs12154239	T	C	T	C	0.00656012	0.0062932	0.5018	0.502593	0.0144087	0.58	0.0010493	4.046E-10
rs12155345	A	C	A	C	0.00770216	-0.0098078	0.6689	0.662823	0.0150046	0.79	0.00111186	4.293E-12
rs12155540	T	C	T	C	0.0117212	0.0330859	0.3295	0.336243	0.0150774	0.015	0.00111398	6.826E-26
rs12158831	T	G	T	G	0.0188537	0.00697802	0.0255	0.022571	0.0485713	0.96	0.00335988	2.009E-08
rs12188092	T	C	T	C	-0.0096224	0.0165014	0.1093	0.109094	0.0228112	0.52	0.00167723	9.647E-09
rs12201073	T	C	T	C	-0.0070757	0.0115174	0.3821	0.386131	0.0145765	0.35	0.00107687	5.021E-11
rs12201105	A	G	A	G	-0.0102199	0.00796383	0.8626	0.859355	0.0204749	0.73	0.0015199	1.767E-11
rs12206040	T	C	T	C	-0.0071428	0.0340924	0.7188	0.714644	0.0156793	0.0239999	0.0012633	1.57E-08
rs12209720	T	C	T	C	-0.0071665	0.00813993	0.7379	0.737638	0.0161722	0.6	0.00118982	1.71E-09
rs12224337	A	G	A	G	-0.0066815	-0.0049421	0.5028	0.50823	0.0141734	0.86	0.00104652	1.718E-10
rs12234936	T	C	T	C	-0.0145951	0.0110373	0.419	0.419884	0.0144207	0.46	0.00106285	6.538E-43
rs12240387	A	G	A	G	-0.0059899	0.00419293	0.3646	0.373791	0.0147047	0.93	0.00108713	3.592E-08
rs12260313	T	C	T	C	0.00713786	-0.0103962	0.2985	0.290027	0.0157051	0.43	0.00114984	5.366E-10
rs12272462	A	G	A	G	0.00747938	0.0129351	0.7325	0.734351	0.016087	0.33	0.00118255	2.531E-10
rs12273435	A	G	A	G	-0.0140853	0.0179147	0.201	0.211332	0.0175725	0.19	0.00131066	6.111E-27
rs12305290	A	G	A	G	-0.0086934	0.0061818	0.1728	0.180196	0.0184956	0.57	0.001384	3.364E-10
rs12313886	T	C	T	C	0.0067258	-0.0040418	0.241	0.235894	0.0169262	0.77	0.00122379	3.881E-08
rs12314891	A	G	A	G	-0.0081434	0.0177827	0.7338	0.731421	0.0160739	0.33	0.00118391	6.037E-12
rs12351052	T	C	T	C	0.00963169	-0.0301446	0.8528	0.852228	0.0199951	0.11	0.00147704	6.972E-11
rs12359372	T	C	T	C	-0.009816	0.0114568	0.6616	0.669992	0.015208	0.34	0.00110632	7.158E-19
rs12362273	A	C	A	C	-0.0070828	0.0230261	0.761	0.762857	0.0167343	0.14	0.00122728	7.885E-09
rs12375473	T	C	T	C	-0.0103433	-0.0251051	0.1053	0.108873	0.022878	0.25	0.00170497	1.308E-09
rs12437429	A	G	A	G	0.00680989	-0.0151504	0.3002	0.303753	0.0155142	0.36	0.00114194	2.474E-09
rs12438304	T	C	T	C	0.0136344	-0.0031439	0.131	0.134131	0.0209977	0.9	0.00155104	1.485E-18
rs12442630	A	T	A	T	0.0232255	-0.0215664	0.9686	0.969245	0.0411251	0.47	0.00300138	1.006E-14
rs12445627	A	G	A	G	-0.0065879	-0.0009356	0.4672	0.472985	0.014298	0.85	0.00104906	3.39E-10
rs12449488	A	G	A	G	-0.0068371	0.0122899	0.681	0.672624	0.0152065	0.35	0.00112342	1.156E-09
rs12468040	T	G	T	G	0.0115338	-0.0111017	0.3774	0.379639	0.014612	0.59	0.00107946	1.196E-26
rs12469557	T	C	T	C	-0.0060698	0.00539871	0.6008	0.6054	0.0146209	0.79	0.00107751	1.768E-08
rs12473986	T	C	T	C	-0.0066777	0.0131153	0.2867	0.286769	0.0157084	0.450001	0.00115708	7.861E-09
rs12496649	T	C	T	C	-0.0067168	-0.0221617	0.5676	0.569066	0.0143234	0.0769999	0.00105899	2.256E-10
rs12506222	T	C	T	C	-0.0142283	0.018086	0.4417	0.424087	0.0143914	0.54	0.00105369	1.503E-41
rs12507497	A	G	A	G	-0.0086305	0.015094	0.6417	0.641636	0.0148421	0.23	0.00109125	2.595E-15
rs12516485	A	G	A	G	0.0125058	0.0148548	0.1251	0.130726	0.0210272	0.54	0.00158808	3.415E-15
rs12518468	T	C	T	C	0.00844692	-0.0097209	0.6672	0.67132	0.0151156	0.43	0.00111106	2.9E-14
rs12523817	T	C	T	C	0.00747152	-0.0021115	0.7563	0.7445	0.0162936	0.94	0.00121881	8.787E-10

rs12524795	T	C	T	C	0.00851288	0.00950184	0.4448	0.45074	0.0142804	0.48				0.00105324	6.323E-16
rs12532008	T	C	T	C	-0.0098941	0.0144767	0.6242	0.627342	0.0146793	0.52				0.00108111	5.613E-20
rs12534982	A	G	A	G	0.00757355	-0.0158643	0.7374	0.730925	0.0161389	0.19				0.00118942	1.922E-10
rs12538157	A	T	A	T	-0.0082205	0.00560105	0.6746	0.68574	0.0153169	0.84				0.00111681	1.825E-13
rs12549644	A	G	A	G	-0.008357	-0.0363677	0.8469	0.853263	0.0201915	0.11				0.00145314	8.885E-09
rs12574281	A	C	A	C	-0.0071516	0.0176825	0.6334	0.619566	0.0146515	0.38				0.00108587	4.517E-11
rs12581070	T	C	T	C	0.0126792	-0.0051548	0.1419	0.136323	0.0206975	0.88				0.00149952	2.774E-17
rs12596890	A	G	A	G	0.0110492	0.0213909	0.7984	0.799575	0.0177352	0.16				0.00130424	2.419E-17
rs12602072	A	G	A	G	0.0093663	-0.0355034	0.8141	0.810843	0.018464	0.04				0.00134832	3.745E-12
rs12609965	A	C	A	C	0.00705976	-0.0222901	0.245	0.250172	0.0163948	0.22	rs7256290	A	G	0.00122411	8.05E-09
rs12615721	A	G	A	G	-0.0116455	0.0056726	0.08	0.074233	0.0270268	0.99				0.00192874	1.564E-09
rs12617656	T	C	T	C	-0.0129625	0.0117703	0.6754	0.688809	0.015385	0.24				0.00127986	4.164E-24
rs12627339	A	T	A	T	0.00977487	0.00364836	0.6982	0.686781	0.0153171	0.83				0.00114004	9.963E-18
rs1263671	T	C	T	C	-0.0087073	0.00477649	0.8335	0.835933	0.0192974	0.62				0.00140564	5.861E-10
rs12637456	A	T	A	T	-0.0115279	-0.0177794	0.2833	0.280564	0.015829	0.23				0.00116134	3.204E-23
rs12638960	A	G	A	G	0.0106866	0.00409168	0.09	0.088898	0.0249752	0.85				0.0018284	5.072E-09
rs12646225	T	C	T	C	0.0118055	0.00721015	0.1099	0.104493	0.0231841	0.73				0.00169908	3.693E-12
rs12649053	T	C	T	C	-0.0070978	-0.003063	0.2252	0.222656	0.0170682	0.760001				0.00125277	1.462E-08
rs1267488	A	G	A	G	-0.0149462	0.0118902	0.8202	0.819848	0.0184818	0.49				0.0013637	5.968E-28
rs12676597	T	C	T	C	0.0106621	-0.0132082	0.3652	0.377091	0.0146715	0.57				0.0010872	1.049E-22
rs12678691	A	G	A	G	0.00683879	-0.0002434	0.513	0.509705	0.0142131	0.95				0.001047	6.512E-11
rs12679743	T	C	T	C	-0.0134837	0.00846784	0.1474	0.140833	0.0207318	0.59				0.00147621	6.602E-20
rs12686734	A	T	A	T	0.00609325	0.00809718	0.408	0.398995	0.0144922	0.7				0.00106483	1.052E-08
rs12692779	T	C	T	C	-0.0105238	0.00462962	0.2748	0.260492	0.0162305	0.89				0.00117246	2.805E-19
rs12699131	A	G	A	G	0.0138205	0.00255808	0.4729	0.475782	0.0141682	0.68				0.00104805	1.045E-39
rs12702698	T	C	T	C	0.00687158	-0.0026467	0.4627	0.474606	0.0143021	0.74				0.00105015	6.007E-11
rs12706031	A	C	A	C	0.00578276	8.7703E-05	0.4553	0.450446	0.0142869	0.99				0.00105071	3.726E-08
rs12709063	A	G	A	G	-0.007071	0.00091736	0.4925	0.489115	0.014192	0.9				0.00104676	1.427E-11
rs12709690	T	C	T	C	-0.0185836	0.0303894	0.3548	0.356675	0.0148129	0.0530005				0.00109364	9.282E-65
rs12713018	A	C	A	C	-0.0089306	-0.0002541	0.6027	0.605935	0.0145441	0.93				0.00106963	6.872E-17
rs12743874	A	T	A	T	-0.0102549	-0.0192752	0.7183	0.7048	0.0155931	0.12				0.00117175	2.104E-18
rs12747808	T	C	T	C	0.00786197	-0.0233807	0.8432	0.836016	0.0191926	0.19				0.00143904	4.666E-08
rs12764593	C	G	C	G	0.021906	-0.0124256	0.0611	0.058658	0.0305124	0.64				0.00219297	1.703E-23
rs12768641	A	G	A	G	0.00940253	0.00394248	0.7614	0.765592	0.0167642	0.64				0.00122886	1.992E-14
rs1278537	T	G	T	G	-0.0067386	0.00528167	0.4803	0.477403	0.0142063	0.719999				0.00104732	1.244E-10
rs12793532	A	G	A	G	-0.0089967	-0.0100856	0.8727	0.867432	0.0209359	0.36				0.00156988	1.001E-08
rs12801458	A	T	A	T	-0.0115148	-0.0094762	0.0784	0.086144	0.0253736	0.86				0.00195144	3.617E-09
rs12814006	T	C	T	C	0.0088049	-0.0287676	0.5259	0.527322	0.0142812	0.0519996				0.00104969	4.933E-17
rs12820589	A	G	A	G	-0.0100402	-0.0021929	0.7831	0.77909	0.0172155	0.88				0.00126998	2.665E-15
rs12821032	A	G	A	G	-0.0070095	0.0386874	0.3879	0.376645	0.0146405	0.0140001				0.00107415	6.765E-11
rs12828220	T	C	T	C	-0.0097468	-0.0007781	0.6333	0.637563	0.0148069	0.99				0.00108581	2.789E-19
rs12893970	A	G	A	G	-0.0127241	0.0740518	0.0983	0.108032	0.0229941	0.00065				0.00175943	4.755E-13
rs12897542	A	G	A	G	-0.0103491	0.0145762	0.8034	0.796896	0.0176476	0.43				0.0013168	3.854E-15
rs12897763	A	G	A	G	0.019682	0.00053754	0.1911	0.187395	0.0181742	0.89				0.00133087	1.73E-49
rs1289861	A	G	A	G	-0.0078402	0.00923699	0.2271	0.22438	0.0170282	0.69				0.00124894	3.449E-10
rs1290505	A	G	A	G	0.00734916	0.0103176	0.41	0.401841	0.0144716	0.53				0.00106388	4.924E-12
rs12912465	T	C	T	C	-0.0091121	0.00677992	0.2689	0.26742	0.0163118	0.54				0.00118885	1.793E-14
rs12915175	A	G	A	G	-0.00832	-0.0246434	0.1833	0.187564	0.0181859	0.29				0.00135294	7.751E-10
rs12916023	A	G	A	G	-0.0084894	-0.0077263	0.4908	0.483482	0.0142214	0.44				0.00104712	5.187E-16
rs1291865	T	G	T	G	-0.0103137	0.00430183	0.5105	0.505202	0.0142763	0.87				0.00104703	6.829E-23
rs12922671	T	G	T	G	0.00910742	0.037777	0.1523	0.150123	0.0202149	0.051				0.00146055	4.49E-10
rs12928216	A	G	A	G	0.00748224	-0.0008139	0.4299	0.437575	0.0143128	0.93				0.00105751	1.491E-12
rs12937411	T	C	T	C	0.0106351	-0.0142323	0.4094	0.408176	0.0144314	0.39				0.00106441	1.655E-23
rs12957463	A	G	A	G	0.0125225	0.0107399	0.7956	0.797574	0.0176484	0.53				0.00130138	6.424E-22
rs12957987	A	G	A	G	0.00865081	0.00982188	0.4783	0.476279	0.0142296	0.48				0.00104749	1.476E-16
rs12962980	C	G	C	G	0.0168181	-0.0028382	0.1822	0.192368	0.0180052	0.91				0.00135555	2.396E-35

rs12972944	A	G	A	G	0.00745531	0.0154288	0.684	0.682315	0.0152348	0.36				0.00112549	3.496E-11
rs12974657	T	C	T	C	0.00916851	-0.0084396	0.6826	0.682087	0.015237	0.59				0.00112529	3.706E-16
rs12976992	A	G	A	G	-0.0063275	-0.0049418	0.5222	0.529167	0.0143224	0.92				0.00105706	2.15E-09
rs12981405	T	C	T	C	-0.0110564	0.017794	0.1656	0.165893	0.0191245	0.43				0.00140805	4.094E-15
rs12983038	A	G	A	G	-0.0080883	-0.0260185	0.1914	0.189256	0.0181352	0.15				0.00133428	1.345E-09
rs13010288	T	G	T	G	0.020104	-0.031009	0.1276	0.133712	0.0209166	0.12				0.00156844	1.298E-37
rs13015323	A	C	A	C	0.00763098	1.1393E-05	0.4435	0.444653	0.0144087	1				0.00105325	4.324E-13
rs13037516	T	C	T	C	0.00588703	0.0052159	0.4984	0.500172	0.0141988	0.630001				0.00104651	1.854E-08
rs13048654	C	G	C	G	-0.0085059	-0.0210217	0.7477	0.736503	0.016238	0.11				0.00120523	1.694E-12
rs13060816	T	C	T	C	-0.0082302	-0.0115736	0.4705	0.466963	0.0142518	0.450001				0.00104833	4.124E-15
rs13061596	A	G	A	G	-0.019479	0.11397	0.045	0.042595	0.0351498	0.00129999				0.00253117	1.405E-14
rs130649	A	G	A	G	0.00935242	0.0405846	0.2544	0.253783	0.0163094	0.00840001				0.00120774	9.639E-15
rs13065045	A	C	A	C	-0.0076317	-0.0188491	0.531	0.526952	0.014232	0.2				0.00104852	3.379E-13
rs13073489	A	G	A	G	-0.0094597	0.0109467	0.7401	0.740712	0.0162352	0.52				0.00119351	2.271E-15
rs13078603	A	G	A	G	0.00758092	0.00583798	0.3962	0.409319	0.0144238	0.43	rs9854306	T	C	0.00106981	1.377E-12
rs13102416	A	C	A	C	-0.0062893	-0.0118917	0.5795	0.571417	0.0143779	0.32				0.00106029	3.005E-09
rs13104877	A	G	A	G	0.0059449	0.0123185	0.4884	0.506129	0.0142234	0.28				0.00105346	1.672E-08
rs13112269	A	G	A	G	0.0107045	0.0357727	0.117	0.122301	0.021827	0.0830004				0.00163243	5.463E-11
rs13121486	A	G	A	G	-0.0075047	0.00207001	0.334	0.338528	0.0150501	0.68				0.00110944	1.335E-11
rs13122283	A	G	A	G	0.00694827	-0.0186316	0.4279	0.436575	0.0143454	0.2				0.00105786	5.084E-11
rs13125074	A	G	A	G	0.00891073	0.0163259	0.2328	0.234853	0.016911	0.39				0.00124105	6.982E-13
rs13133213	A	G	A	G	0.00873111	-0.0102745	0.4994	0.493886	0.0141701	0.53				0.00104651	7.243E-17
rs13135092	A	G	A	G	0.0299025	-0.016554	0.9129	0.916522	0.0257761	0.43				0.00186124	4.441E-58
rs13138842	A	G	A	G	0.00834251	0.00852918	0.197	0.19696	0.0178396	0.67				0.00132113	2.703E-10
rs13140041	A	G	A	G	-0.0063595	0.00905506	0.5414	0.545452	0.0142609	0.41				0.00105259	1.524E-09
rs13158057	T	C	T	C	0.00685253	-0.0121249	0.3254	0.332564	0.015047	0.51				0.00111727	8.616E-10
rs13159124	A	G	A	G	-0.0112665	0.0145863	0.6795	0.668917	0.0150909	0.34				0.00112212	1.012E-23
rs13161488	T	C	T	C	-0.0086917	-0.0107346	0.648	0.636074	0.0147899	0.32				0.00109802	2.45E-15
rs13163062	T	C	T	C	0.00939159	-0.0417582	0.426	0.435597	0.0143693	0.00389996				0.0010583	7.033E-19
rs13164530	T	G	T	G	-0.0100803	0.0272368	0.1309	0.125573	0.0217042	0.16				0.00155155	8.196E-11
rs13169187	A	G	A	G	-0.006982	-0.0067348	0.5398	0.53536	0.0142368	0.55				0.00104998	2.941E-11
rs1320654	A	C	A	C	0.00600135	-0.0263427	0.395	0.388069	0.0145662	0.089				0.00107038	2.058E-08
rs13212041	T	C	T	C	0.0112336	0.0283101	0.7995	0.795835	0.0176316	0.11				0.00130851	9.056E-18
rs13238939	T	C	T	C	-0.0074278	0.0229307	0.5065	0.511029	0.0142195	0.11				0.0010466	1.278E-12
rs13244634	T	C	T	C	-0.0078389	0.0553748	0.2363	0.23198	0.0167752	0.00091				0.00123364	2.099E-10
rs13247375	T	C	T	C	-0.0098064	0.0207617	0.2062	0.209338	0.0174433	0.16				0.0012937	3.445E-14
rs13252156	A	C	A	C	0.00898821	-0.0098213	0.4	0.395207	0.0145899	0.54				0.00107076	4.686E-17
rs13254311	A	G	A	G	0.0110242	-0.0229198	0.6753	0.6757	0.0151725	0.14				0.00111775	6.029E-23
rs13255037	T	C	T	C	0.0059172	-0.0075922	0.4895	0.460865	0.0143115	0.68	rs2517333	T	C	0.00104697	1.591E-08
rs13278836	A	G	A	G	0.013733	0.00560483	0.1155	0.113036	0.0225958	0.630001				0.00163796	5.096E-17
rs13289150	A	G	A	G	-0.0059928	0.00296287	0.6185	0.623036	0.0146444	0.709999				0.00107734	2.655E-08
rs1329125	T	C	T	C	-0.0124262	0.0345357	0.3254	0.327565	0.015211	0.0129999				0.00111796	1.059E-28
rs13296345	T	C	T	C	0.00867033	-0.0046083	0.6249	0.62971	0.0147695	0.8				0.00108091	1.046E-15
rs13298735	T	C	T	C	-0.0168103	-0.0289028	0.9643	0.96602	0.0397516	0.649999				0.00282146	2.547E-09
rs1332894	T	C	T	C	-0.0119258	0.0324614	0.134	0.141349	0.0203602	0.16				0.00153637	8.362E-15
rs13332638	A	G	A	G	-0.0130358	0.0187101	0.0862	0.07613	0.0269153	0.77				0.00186968	3.118E-12
rs1334205	A	C	A	C	0.00617869	0.00063889	0.3324	0.337208	0.0150751	0.98				0.00111091	2.668E-08
rs1334297	A	G	A	G	0.0239964	-0.0257744	0.7407	0.735519	0.0161113	0.0840001				0.00119412	8.08E-90
rs13361997	A	C	A	C	0.00793435	0.0271819	0.8446	0.85274	0.0200725	0.0980009				0.0014445	3.948E-08
rs13376365	T	C	T	C	0.00903893	-0.0128928	0.7878	0.786528	0.0173696	0.35				0.00127977	1.635E-12
rs1337731	A	G	A	G	0.00863246	0.034846	0.3344	0.329408	0.0151012	0.0359998				0.0011091	7.073E-15
rs1338263	A	G	A	G	-0.008005	-0.0014374	0.5467	0.549308	0.0143415	0.79				0.0010511	2.622E-14
rs1338955	A	G	A	G	-0.0131226	0.028683	0.0762	0.075432	0.0270227	0.25				0.00197273	2.899E-11
rs13403620	T	G	T	G	0.00586601	-0.0151517	0.5147	0.503487	0.0142798	0.23				0.00104696	2.109E-08
rs13413752	A	G	A	G	-0.0175642	0.027119	0.0299	0.034856	0.0387228	0.32				0.00308364	1.224E-08
rs13422459	T	C	T	C	-0.0068708	0.0174187	0.2386	0.237384	0.0168515	0.19				0.00122799	2.207E-08

rs13429686	A	G	A	G	0.0145425	-0.0246288	0.8725	0.872659	0.021277	0.25				0.00156882	1.862E-20
rs1344594	C	G	C	G	0.0124182	-0.0048342	0.0766	0.0791	0.0264458	0.88				0.00196873	2.838E-10
rs134545	T	C	T	C	-0.0079994	0.0152477	0.6426	0.62527	0.0146589	0.709999				0.00109195	2.377E-13
rs1351665	T	C	T	C	0.00818289	-0.0059791	0.4491	0.459226	0.0142434	0.95				0.00105197	7.354E-15
rs1353286	T	G	T	G	0.00663949	-0.0275618	0.537	0.546046	0.0142788	0.0850002				0.00105002	2.561E-10
rs1357913	A	G	A	G	0.00713999	0.0154347	0.5839	0.602074	0.0144974	0.34	rs7465344	T	C	0.00106826	2.325E-11
rs135896	A	G	A	G	0.00718169	0.0288814	0.7832	0.779297	0.0171208	0.0629999				0.00126983	1.554E-08
rs1361580	C	G	C	G	0.00961622	-0.0149021	0.2066	0.209154	0.0177341	0.44				0.00129295	1.029E-13
rs1365355	A	G	A	G	-0.0059998	-0.005919	0.5847	0.585461	0.0144279	0.53				0.00106435	1.728E-08
rs137121	T	C	T	C	0.0126618	-0.0124452	0.127	0.134704	0.0207766	0.84				0.00157243	8.117E-16
rs1372086	A	G	A	G	-0.0101955	-0.0073959	0.8461	0.835987	0.0191459	0.41				0.00145005	2.048E-12
rs1375013	T	C	T	C	-0.0091632	0.00224233	0.6752	0.663193	0.0151263	0.84				0.00112027	2.85E-16
rs1378893	A	G	A	G	0.00907707	-0.0130769	0.2379	0.240027	0.0166419	0.4				0.00122904	1.52E-13
rs137926116	T	C	T	C	-0.0220605	-0.0268614	0.0184	0.015589	0.0580992	0.709999				0.00391405	1.736E-08
rs1380669	A	G	A	G	0.0092445	-0.0161807	0.7552	0.76562	0.0169435	0.48				0.00121696	3.039E-14
rs1382693	A	T	A	T	-0.0062293	-0.0045986	0.6055	0.605369	0.0145365	0.6	rs9808903	C	T	0.00107061	5.935E-09
rs138334012	T	C	T	C	-0.0345106	-0.0264083	0.9928	0.994783	0.101554	0.92				0.00632935	4.975E-08
rs1387148	A	C	A	C	-0.0094556	-0.0018402	0.1982	0.20453	0.0176352	0.99				0.00131296	5.938E-13
rs138850618	T	C	T	C	0.0198024	-0.0281468	0.9726	0.96842	0.0412205	0.57				0.00323728	9.521E-10
rs1389997	T	G	T	G	0.00791109	-0.0055108	0.3238	0.316361	0.0152585	0.49				0.00111824	1.496E-12
rs1392816	T	C	T	C	0.00959338	-0.007359	0.3814	0.388078	0.0146868	0.66				0.00107755	5.424E-19
rs1396558	A	G	A	G	-0.0076254	-0.015311	0.4505	0.466061	0.0142387	0.31				0.00105181	4.167E-13
rs1407350	T	G	T	G	0.00784214	0.00992965	0.651	0.66121	0.0151658	0.3	rs10738158	T	C	0.00109791	9.129E-13
rs141503359	T	C	T	C	-0.0283543	-0.0142089	0.013	0.012799	0.0649536	0.67				0.00477892	2.973E-09
rs1418004	T	C	T	C	-0.0080931	0.0191008	0.4184	0.416375	0.0144354	0.15				0.00106073	2.352E-14
rs14184	C	G	C	G	-0.0134303	-0.0055376	0.7007	0.704939	0.0155981	0.649999				0.00114292	6.957E-32
rs142237780	T	C	T	C	0.01756	-0.104313	0.0368	0.03998	0.0373656	0.00430002				0.0028203	4.772E-10
rs1427298	T	C	T	C	0.00866701	0.0133974	0.4237	0.428027	0.0144929	0.23				0.0010599	2.912E-16
rs1427323	T	C	T	C	0.00676413	-0.0012306	0.313	0.30831	0.0154069	0.91				0.0011284	2.042E-09
rs1428102	A	G	A	G	-0.0068044	0.010989	0.3866	0.373968	0.0148003	0.83				0.00107491	2.442E-10
rs1430047	A	G	A	G	-0.0090251	0.0077661	0.7329	0.727103	0.0159353	0.8				0.00118264	2.318E-14
rs143095689	T	C	T	C	0.0103051	0.0426883	0.1857	0.185198	0.0183554	0.00980009				0.00134559	1.877E-14
rs143141266	T	C	T	C	-0.0140975	-0.0203707	0.0453	0.041261	0.0361266	0.56				0.00253222	2.588E-08
rs143747834	C	G	C	G	-0.0078207	0.0141896	0.2053	0.197078	0.0178689	0.44	rs190523649	T	A	0.00129544	1.57E-09
rs1440980	T	C	T	C	0.0141491	0.00421035	0.2287	0.218893	0.0172009	0.79				0.00124875	9.245E-30
rs144336753	A	G	A	G	0.0395657	-0.0421319	0.0176	0.019255	0.051773	0.38				0.00399182	3.708E-23
rs1447200	A	C	A	C	0.00901162	0.0135023	0.4944	0.487044	0.014213	0.41				0.00104657	7.256E-18
rs1448355	T	C	T	C	0.00851076	-0.0054562	0.6238	0.618449	0.0146681	0.69				0.00108044	3.349E-15
rs1452366	T	G	T	G	-0.0119117	-0.0150201	0.3518	0.344384	0.0149244	0.29				0.00109575	1.583E-27
rs145481578	T	C	T	C	0.0235412	0.057037	0.0199	0.017076	0.0564282	0.41				0.00386173	1.087E-09
rs145979116	T	G	T	G	-0.0321899	0.00217139	0.9788	0.978336	0.0487703	0.9				0.00370438	3.639E-18
rs1462852	A	T	A	T	-0.0072892	0.0018981	0.6671	0.676337	0.0152005	0.61				0.00111068	5.273E-11
rs1464018	T	C	T	C	-0.0136104	0.0107027	0.2106	0.204211	0.017603	0.780001				0.00128332	2.812E-26
rs1464297	T	C	T	C	-0.0106953	0.0273536	0.6515	0.654537	0.0149948	0.0659994				0.00109813	2.052E-22
rs146851424	A	C	A	C	-0.0198516	-0.0181823	0.9774	0.978379	0.0491828	0.91				0.0035231	1.756E-08
rs1470801	A	G	A	G	-0.008922	-0.0163567	0.1952	0.190709	0.0180747	0.38				0.00132017	1.394E-11
rs1479119	A	G	A	G	-0.0058579	0.00341472	0.438	0.438084	0.0144782	0.81				0.00105471	2.793E-08
rs1483148	T	G	T	G	0.0120892	-0.0121394	0.7312	0.722765	0.01585	0.34				0.00118026	1.28E-24
rs148512052	A	G	A	G	-0.0161685	0.0562399	0.034	0.031542	0.0411444	0.2				0.00289014	2.21E-08
rs1485272	T	C	T	C	-0.0071811	0.0273276	0.6535	0.657933	0.01499	0.0479999				0.00110188	7.172E-11
rs1492014	T	C	T	C	0.00728542	-0.0040417	0.5767	0.564834	0.0143014	0.73				0.00105965	6.188E-12
rs1492027	A	G	A	G	0.00743534	-0.0139478	0.7806	0.776078	0.0171449	0.37				0.00126474	4.125E-09
rs1493159	T	C	T	C	0.0150309	-0.0372245	0.8453	0.836792	0.0192289	0.0420001				0.00144698	2.828E-25
rs149352678	T	C	T	C	0.0100909	-0.0033326	0.0979	0.093539	0.0245043	0.97				0.00176559	1.094E-08
rs149914551	A	C	A	C	0.0172234	-0.0407696	0.0349	0.035481	0.0384609	0.23				0.00295069	5.304E-09
rs150121450	T	G	T	G	0.019662	0.0656122	0.0307	0.033639	0.0394271	0.0739997				0.00304161	1.017E-10

rs150470230	A	G	A	G	0.0214124	-0.0236439	0.9723	0.971462	0.0427595	0.6				0.00322911	3.333E-11
rs1505676	C	G	C	G	0.0066757	0.0293849	0.6275	0.627523	0.0146505	0.0329997				0.00108229	6.909E-10
rs151144406	T	C	T	C	-0.0182101	-0.0434474	0.9716	0.969888	0.0445371	0.35				0.00325779	2.278E-08
rs1511473	C	G	C	G	0.00734157	0.0164083	0.191	0.193754	0.0179721	0.24				0.00133113	3.478E-08
rs1517480	T	G	T	G	0.0127472	0.0178127	0.1985	0.200369	0.0177194	0.16				0.00131184	2.544E-22
rs1527878	A	G	A	G	-0.0101417	0.0136443	0.7522	0.742368	0.0162958	0.31	rs1918700	G	T	0.00121198	5.855E-17
rs153098	A	C	A	C	-0.0066585	-0.0071502	0.531	0.535551	0.0143618	0.69				0.00104852	2.151E-10
rs1539414	A	G	A	G	-0.0107426	0.0401314	0.2121	0.212455	0.0173314	0.00430002				0.00127999	4.765E-17
rs1544	A	G	A	G	-0.0077654	-0.009626	0.2713	0.265298	0.016051	0.48				0.00117698	4.182E-11
rs1547173	A	G	A	G	-0.0058983	0.00295262	0.4798	0.482775	0.0142127	0.85				0.0010475	1.798E-08
rs1561743	C	G	C	G	0.00707504	-0.0129546	0.7591	0.761552	0.016727	0.42				0.00122396	7.455E-09
rs1581168	T	C	T	C	-0.0101066	0.00281625	0.2879	0.282067	0.0158176	0.87				0.00115564	2.215E-18
rs1585272	T	C	T	C	0.00689152	0.0138569	0.6531	0.646075	0.014863	0.2				0.00109931	3.633E-10
rs158839	A	C	A	C	-0.0062345	-0.0233337	0.612	0.61695	0.0147288	0.11				0.00107393	6.416E-09
rs1610243	T	C	T	C	-0.0063579	-0.001139	0.6186	0.614224	0.0146006	0.89				0.00107735	3.598E-09
rs1630579	T	C	T	C	-0.0099998	0.00048257	0.781	0.780051	0.0171328	0.98				0.00126562	2.771E-15
rs1632822	C	G	C	G	0.00587519	-0.0068434	0.382	0.37521	0.0146946	0.6				0.00107707	4.912E-08
rs1637492	A	G	A	G	-0.0064216	-0.0056488	0.4916	0.493111	0.0141808	0.780001				0.00104675	8.531E-10
rs1638526	T	C	T	C	0.00888041	0.00821222	0.7414	0.734426	0.0160658	0.77				0.00119501	1.076E-13
rs1647267	A	T	A	T	0.0087825	-0.0085484	0.62	0.610687	0.0145466	0.64				0.00107801	3.723E-16
rs1648404	T	C	T	C	0.00775717	0.02119	0.4732	0.476192	0.0142239	0.12				0.00104801	1.341E-13
rs164938	T	G	T	G	-0.0080638	0.0144822	0.3936	0.389695	0.0145524	0.42				0.00107104	5.122E-14
rs1663564	A	G	A	G	0.0169952	0.0129108	0.9116	0.093485	0.024659	0.49	rs1196760	G	C	0.00264908	1.402E-10
rs1670459	C	G	C	G	-0.0080358	-0.0067933	0.6083	0.597279	0.0144805	0.33				0.00107195	6.552E-14
rs16844417	A	G	A	G	-0.0093193	-0.0085416	0.1166	0.12361	0.0217102	0.99				0.00163036	1.09E-08
rs16854920	T	C	T	C	-0.0083397	0.00231655	0.6632	0.655889	0.0149201	0.88				0.00110975	5.707E-14
rs16864081	T	C	T	C	0.00691124	0.012522	0.2375	0.237462	0.0168468	0.51				0.00122994	1.917E-08
rs16878083	A	G	A	G	-0.0071933	0.023296	0.7709	0.781073	0.017162	0.19				0.00124509	7.573E-09
rs16890200	T	C	T	C	-0.0124303	0.00374109	0.067	0.071884	0.0274688	0.97	rs16890269	C	G	0.00209394	2.916E-09
rs1689510	C	G	C	G	0.0174406	-0.021699	0.3272	0.338472	0.0150005	0.18				0.00111523	3.98E-55
rs1691017	A	G	A	G	0.00631092	-0.0065314	0.4754	0.476919	0.0142795	0.77				0.00104778	1.707E-09
rs16917562	A	T	A	T	-0.0134561	0.00552223	0.8844	0.889726	0.02273	0.68				0.00163781	2.109E-16
rs16919774	A	T	A	T	-0.0093474	-0.0413616	0.7873	0.790394	0.0174565	0.017				0.00127867	2.666E-13
rs16962845	T	C	T	C	-0.0131864	0.0396124	0.8932	0.902161	0.0239193	0.0569994				0.00169437	7.115E-15
rs16966271	T	C	T	C	0.00998087	-0.0028326	0.2831	0.280601	0.015868	0.51				0.00116207	8.781E-18
rs16970633	T	G	T	G	-0.0111471	-0.0175361	0.1582	0.164061	0.0191585	0.38				0.00143404	7.663E-15
rs16983844	A	G	A	G	0.0107414	0.0190295	0.2089	0.216379	0.0172453	0.24				0.00128902	7.862E-17
rs16993330	A	C	A	C	-0.0239806	0.0186574	0.0587	0.053498	0.0315437	0.630001				0.00223126	6.064E-27
rs170035	A	G	A	G	0.0084942	0.0306286	0.6208	0.589385	0.0146653	0.0230001	rs10030562	C	T	0.00108143	4.007E-15
rs17014412	A	G	A	G	-0.0201397	0.018307	0.0341	0.03679	0.0376581	0.44				0.00288665	3.02E-12
rs17088142	T	G	T	G	-0.0068268	-0.0091006	0.4691	0.461763	0.0143356	0.38				0.00104865	7.51E-11
rs17092597	A	G	A	G	0.0170589	0.00354936	0.0506	0.046452	0.0336777	0.95				0.00239216	9.928E-13
rs17096452	A	T	A	T	0.0113818	-0.0332919	0.783	0.781769	0.0171861	0.0539995				0.00126992	3.175E-19
rs17126938	T	C	T	C	-0.0126379	0.032061	0.8604	0.86268	0.0206964	0.11				0.00151	5.793E-17
rs171697	C	G	C	G	0.0151658	-0.0005184	0.6689	0.671704	0.0151	0.88				0.00111201	2.366E-42
rs17184588	T	C	T	C	-0.0069339	0.0108848	0.513	0.496763	0.0143477	0.56				0.00104911	3.864E-11
rs17184889	T	C	T	C	0.00907646	-0.0033636	0.8847	0.876955	0.0215981	0.97				0.00163832	3.02E-08
rs17224289	T	C	T	C	-0.0131703	0.0560986	0.1197	0.119271	0.0219115	0.00830004				0.00161215	3.095E-16
rs17234990	T	C	T	C	-0.0083571	0.0371077	0.6802	0.68035	0.0152265	0.0140001				0.0011219	9.375E-14
rs17236381	A	C	A	C	0.00846417	-0.0037864	0.1368	0.137492	0.0208589	0.760001				0.00152333	2.75E-08
rs17248751	A	G	A	G	-0.0137524	-0.0008623	0.7846	0.773446	0.0169707	0.6				0.00127334	3.424E-27
rs17250623	A	G	A	G	-0.0070034	0.00829421	0.6872	0.679091	0.0151891	0.760001				0.00112874	5.47E-10
rs17257579	T	C	T	C	-0.0131005	-0.0044702	0.8755	0.870366	0.021162	0.75				0.0015851	1.403E-16
rs17276956	A	C	A	C	-0.0079521	0.00166316	0.5213	0.501937	0.0142326	0.75				0.00104789	3.226E-14
rs17311988	T	C	T	C	-0.0275388	0.0199606	0.0191	0.01933	0.0515157	0.709999				0.00388753	1.401E-12
rs17361576	A	C	A	C	0.00840154	-0.0182518	0.4114	0.440778	0.0142366	0.12	rs7672160	C	A	0.00106333	2.771E-15

rs17365719	T	C	T	C	0.00890498	0.0304554	0.1541	0.150148	0.0199368	0.14				0.00145027	8.239E-10
rs1738050	C	G	C	G	-0.0110034	0.0268287	0.6159	0.616526	0.0146092	0.0830004				0.00107581	1.488E-24
rs17400325	T	C	T	C	-0.0242337	0.0883132	0.9597	0.958446	0.035539	0.0109999				0.0026674	1.038E-19
rs17404787	A	T	A	T	-0.0064897	-0.0058135	0.6858	0.681998	0.0153035	0.55				0.00112754	8.613E-09
rs17426562	A	G	A	G	0.0169736	0.00768112	0.0646	0.062406	0.0293261	0.99				0.00212862	1.532E-15
rs174434	T	C	T	C	-0.0057847	0.00241843	0.5142	0.52113	0.0142175	0.89	rs435193	A	G	0.00104693	3.284E-08
rs17463127	A	T	A	T	-0.0082609	-0.0030469	0.7348	0.729082	0.0159573	0.95				0.00118549	3.203E-12
rs17468213	A	T	A	T	0.0100301	0.0108209	0.2756	0.278234	0.0159444	0.33				0.00117107	1.083E-17
rs174768	T	C	T	C	0.0103903	-0.0277239	0.8562	0.85058	0.0199176	0.11				0.00149123	3.224E-12
rs17503473	T	C	T	C	0.0119359	0.00964163	0.6977	0.658193	0.0150233	0.3	rs62379847	C	A	0.0011395	1.129E-25
rs17515793	T	C	T	C	0.00677432	0.0121441	0.3512	0.34866	0.0149008	0.35				0.00109676	6.551E-10
rs17516383	A	G	A	G	0.00687623	-0.0312051	0.3112	0.308321	0.0153764	0.061				0.00115985	3.059E-09
rs17520385	T	C	T	C	0.00608407	-0.0094426	0.6719	0.677611	0.0152365	0.8				0.00111504	4.867E-08
rs17522122	T	G	T	G	-0.0118007	0.0211299	0.4767	0.471236	0.0142632	0.0909997				0.00104866	2.238E-29
rs17557610	T	C	T	C	-0.0099716	0.0175224	0.197	0.201952	0.0176704	0.29				0.00131576	3.484E-14
rs17561583	T	C	T	C	-0.0097118	0.0126394	0.1368	0.135887	0.0207082	0.56				0.0015271	2.026E-10
rs17563464	A	C	A	C	-0.0126827	-0.0238961	0.2189	0.223299	0.0173116	0.15				0.00127379	2.354E-23
rs17565975	A	G	A	G	-0.0121073	-0.0176243	0.5529	0.563123	0.0143745	0.27				0.00105241	1.25E-30
rs17598373	T	C	T	C	0.00731426	-0.023484	0.5573	0.549905	0.014285	0.16				0.00105374	3.888E-12
rs17631311	C	G	C	G	0.00704542	-0.011193	0.5893	0.586366	0.0144229	0.47				0.00106361	3.496E-11
rs17639442	T	G	T	G	-0.0134102	-0.0012599	0.9161	0.909193	0.0247625	0.87				0.00189036	1.301E-12
rs17649841	A	C	A	C	-0.008005	0.0120436	0.2356	0.23929	0.0166512	0.35				0.001233	8.445E-11
rs17669337	T	C	T	C	-0.0100845	-0.0176881	0.4117	0.412946	0.0143892	0.21				0.00106336	2.458E-21
rs17706089	T	C	T	C	-0.0080658	-0.0374245	0.8156	0.816408	0.0183553	0.0290001				0.00134991	2.305E-09
rs17721809	A	G	A	G	-0.0062254	-0.0177933	0.695	0.687891	0.0154248	0.28				0.00113933	4.646E-08
rs17742342	A	C	A	C	-0.0126672	0.0287032	0.799	0.799171	0.0177218	0.12				0.00130569	2.973E-22
rs17743339	A	G	A	G	0.00892176	-0.0178549	0.2476	0.260962	0.0161511	0.31				0.00121247	1.86E-13
rs17760841	T	C	T	C	0.0077451	0.00119512	0.2325	0.22693	0.0170629	0.82				0.00123869	4.039E-10
rs17835368	A	C	A	C	0.00973189	-0.0070931	0.3344	0.329941	0.0151102	0.62				0.00111585	2.748E-18
rs1786313	T	C	T	C	0.00770977	-0.0017115	0.3137	0.308706	0.0153746	0.98	rs1693559	C	T	0.00112786	8.148E-12
rs17881016	A	G	A	G	-0.0131417	-0.0007349	0.739	0.730503	0.0161372	0.9				0.00119787	5.273E-28
rs179942	T	C	T	C	-0.0064765	0.0100204	0.4019	0.396633	0.0150373	0.630001				0.00107237	1.548E-09
rs1809471	T	C	T	C	0.00642724	0.0235674	0.4658	0.475037	0.0142428	0.0719996				0.00104926	9.03E-10
rs1810649	T	C	T	C	-0.006482	0.00580829	0.2759	0.276815	0.0160513	0.61				0.00117068	3.084E-08
rs181164682	A	T	A	T	0.0243553	0.0580233	0.02	0.022089	0.0487917	0.35	rs149541331	C	T	0.00374831	8.159E-11
rs1817452	T	C	T	C	-0.0087385	0.008357	0.8315	0.826175	0.0188187	0.83				0.00139792	4.072E-10
rs1820986	A	G	A	G	-0.0116032	0.025897	0.1373	0.138293	0.020618	0.22				0.00152079	2.349E-14
rs1841023	A	C	A	C	-0.0091126	0.00598346	0.6892	0.687195	0.0153667	0.73				0.00113135	7.962E-16
rs184654	C	G	C	G	0.016505	0.0231083	0.8416	0.83958	0.0193888	0.14				0.00143352	1.129E-30
rs1851013	T	C	T	C	-0.0095048	0.0189979	0.374	0.361127	0.0147896	0.22				0.00108173	1.544E-18
rs185248842	T	C	T	C	0.0221283	0.111104	0.0276	0.020717	0.0513736	0.015				0.00325004	9.831E-12
rs1857508	C	G	C	G	0.00592291	0.0130985	0.386	0.393118	0.0145805	0.21				0.00107482	3.57E-08
rs1861109	C	G	C	G	0.00656955	0.0335299	0.3625	0.366223	0.0147521	0.00909997				0.00108847	1.584E-09
rs1861786	A	G	A	G	-0.0076438	0.0165942	0.3807	0.383427	0.0146015	0.27				0.00107763	1.311E-12
rs1865407	A	G	A	G	-0.0153677	-0.0151929	0.1296	0.134228	0.0209296	0.58				0.00155814	6.044E-23
rs1866710	A	G	A	G	-0.0144898	0.0338252	0.2828	0.319565	0.0152679	0.0269998	rs7102042	T	C	0.00116186	1.069E-35
rs1866823	A	G	A	G	0.0105762	-0.0285464	0.5413	0.544616	0.0144064	0.0389996				0.00105109	8.099E-24
rs1868502	T	C	T	C	0.00724392	-0.0230006	0.2114	0.206045	0.0175663	0.17				0.00128154	1.581E-08
rs1871745	A	G	A	G	0.0101912	0.0248221	0.7901	0.796395	0.0176371	0.1				0.00128506	2.186E-15
rs1880088	A	T	A	T	-0.0077297	0.0208365	0.264	0.255826	0.0162775	0.17				0.00118706	7.429E-11
rs188029	A	G	A	G	-0.0075879	0.0370271	0.27	0.271701	0.0159974	0.02				0.00117861	1.208E-10
rs1880692	A	G	A	G	0.00766629	0.0195676	0.5376	0.532996	0.014274	0.21				0.00104948	2.78E-13
rs188192	A	C	A	C	0.00837883	-0.0051958	0.2938	0.28756	0.0156643	0.56	rs39998	A	C	0.00114922	3.08E-13
rs1881975	A	G	A	G	0.00753487	-0.0099402	0.7657	0.756886	0.0166406	0.54				0.00123886	1.185E-09
rs1888765	C	G	C	G	0.00900915	0.0250761	0.7758	0.784224	0.0172726	0.0739997				0.00125516	7.091E-13
rs1889438	A	G	A	G	-0.0081154	-0.0135327	0.2804	0.282261	0.0157713	0.36				0.00116487	3.247E-12

rs1899888	A	C	A	C	0.00689062	0.00051407	0.4935	0.504932	0.0142185	0.77				0.00104673	4.609E-11
rs1904132	T	C	T	C	-0.0084553	0.0129189	0.3304	0.321565	0.015255	0.47				0.00111455	3.3E-14
rs1904823	T	C	T	C	-0.012007	-0.0125405	0.2492	0.253345	0.0163415	0.66				0.0012097	3.233E-23
rs190737	A	C	A	C	-0.006132	-0.005392	0.5287	0.521669	0.0141986	0.649999				0.00104857	4.968E-09
rs1910005	T	C	T	C	-0.0091214	-0.0130955	0.7124	0.711061	0.0156414	0.48				0.00115615	3.025E-15
rs191103	T	C	T	C	0.00618481	-0.0124561	0.4862	0.491471	0.0142137	0.4				0.00104747	3.534E-09
rs191413135	A	G	A	G	0.0312878	0.0700725	0.011	0.012125	0.0667752	0.24				0.00515609	1.295E-09
rs1921608	A	G	A	G	0.00982504	0.00685893	0.615	0.615737	0.0145955	0.6				0.00107566	6.586E-20
rs192230289	T	C	T	C	-0.026646	-0.0685665	0.0171	0.019544	0.0519045	0.17				0.00405919	5.236E-11
rs192436652	T	C	T	C	-0.0276467	-0.0054057	0.0262	0.027271	0.0436598	0.85				0.00332857	9.902E-17
rs1934619	A	G	A	G	-0.0086012	0.00086266	0.2808	0.292288	0.0156364	0.94				0.00116436	1.502E-13
rs1937526	T	C	T	C	0.00766766	0.00539811	0.3785	0.390321	0.0145507	0.53				0.00107899	1.191E-12
rs1939800	T	C	T	C	0.00827494	-0.0158257	0.3833	0.386019	0.0145769	0.41				0.00107658	1.51E-14
rs1944386	T	C	T	C	-0.0082203	0.0098828	0.6447	0.656065	0.0149732	0.38				0.00109329	5.509E-14
rs1948897	A	G	A	G	-0.0073791	-0.0045	0.5555	0.560703	0.0146567	0.53				0.00105519	2.682E-12
rs1950265	A	C	A	C	0.00752506	-0.0131094	0.4382	0.447284	0.0143	0.37				0.00105489	9.789E-13
rs1961225	T	C	T	C	0.00771508	-0.0220093	0.4208	0.412209	0.0144379	0.0980009				0.00105998	3.372E-13
rs1971218	A	G	A	G	0.00853371	-0.0329018	0.5241	0.559379	0.0144167	0.012	rs1989717	A	G	0.0010544	5.785E-16
rs1972863	A	G	A	G	-0.0094564	-0.0056836	0.3049	0.305483	0.0154363	0.630001				0.00113661	8.811E-17
rs1973419	T	C	T	C	0.00750579	-0.0080485	0.4903	0.485358	0.0142504	0.54				0.00104684	7.513E-13
rs1974708	T	C	T	C	-0.0075487	0.00739124	0.1829	0.177651	0.0185736	0.49				0.00135498	2.534E-08
rs2001628	A	G	A	G	0.00628593	0.0140667	0.6133	0.614889	0.0146063	0.43				0.0010749	4.978E-09
rs2003850	T	C	T	C	0.00747349	-0.0187619	0.3212	0.325165	0.0151656	0.16				0.00112075	2.591E-11
rs2006853	A	G	A	G	0.00924513	0.0134246	0.5875	0.593768	0.0150937	0.34				0.00106525	3.993E-18
rs2008486	A	C	A	C	-0.0062508	-0.0091795	0.5046	0.506546	0.0142137	0.58				0.00104655	2.337E-09
rs2011074	T	C	T	C	0.00825671	0.00501668	0.3521	0.341957	0.0150921	0.68				0.00109599	4.943E-14
rs2017850	T	C	T	C	0.00949171	0.00426636	0.3475	0.343438	0.0149899	0.74				0.00109887	5.746E-18
rs2021871	T	C	T	C	0.00654473	0.0265795	0.6738	0.682781	0.0153042	0.0700003				0.00111842	4.866E-09
rs2026037	T	C	T	C	0.015448	-0.0052862	0.8408	0.832569	0.0191189	0.69				0.00143384	4.557E-27
rs2028789	T	C	T	C	0.00913871	-0.0057287	0.1378	0.139444	0.0204877	0.760001	rs996463	T	C	0.00151824	1.755E-09
rs2035891	C	G	C	G	0.00605581	0.0156467	0.406	0.39239	0.014609	0.39				0.00106565	1.323E-08
rs2041543	T	C	T	C	0.00913945	-0.0170272	0.1587	0.148717	0.0200626	0.26				0.00143221	1.752E-10
rs2050256	A	G	A	G	0.0155544	-0.0350006	0.8284	0.829858	0.0189354	0.0560003				0.0013897	4.421E-29
rs2050846	T	C	T	C	-0.0062582	-0.0036275	0.3387	0.338581	0.0150685	0.89				0.00110867	1.655E-08
rs2054805	T	C	T	C	0.00908304	-0.0076677	0.725	0.73691	0.016186	0.66	rs13265644	T	G	0.00117202	9.216E-15
rs2055940	A	G	A	G	0.00703177	-0.0135859	0.3231	0.329973	0.0151568	0.46				0.00111919	3.319E-10
rs2063569	C	G	C	G	-0.0096189	0.0130025	0.3731	0.377191	0.0146615	0.42				0.00108193	6.076E-19
rs2065057	A	T	A	T	0.00822781	-0.013632	0.7055	0.711558	0.0156938	0.58				0.00114891	7.974E-13
rs2071387	A	G	A	G	0.00901585	-0.0097753	0.8179	0.818053	0.0183554	0.64				0.00135584	2.937E-11
rs2076312	A	C	A	C	-0.0096856	-0.0164292	0.1646	0.16649	0.0190354	0.4	rs2855426	A	G	0.00141226	6.978E-12
rs2078357	C	G	C	G	-0.0091519	-0.0044387	0.8852	0.893327	0.0232028	0.91				0.00164595	2.696E-08
rs2081201	T	C	T	C	0.00621966	0.00010774	0.6199	0.616222	0.0147751	0.99				0.00107873	8.122E-09
rs2088913	A	G	A	G	0.0065382	0.0130356	0.5699	0.57145	0.0143735	0.37				0.0010572	6.231E-10
rs2097532	T	C	T	C	0.00827667	-0.003799	0.6437	0.641024	0.0147798	0.780001				0.00109335	3.74E-14
rs2098432	A	G	A	G	-0.0087552	-0.0092139	0.3665	0.36435	0.0147633	0.5				0.00108593	7.49E-16
rs2104598	A	G	A	G	-0.006651	-0.0289999	0.53	0.527335	0.0145091	0.0439997	rs11196169	A	G	0.00105183	2.562E-10
rs210608	T	C	T	C	0.00595412	-0.0162489	0.4419	0.438171	0.014348	0.35				0.00105364	1.593E-08
rs211283	C	G	C	G	-0.0092236	-0.0019533	0.24	0.241497	0.0165503	0.760001				0.00122534	5.175E-14
rs213014	T	C	T	C	-0.0067334	0.0209157	0.5459	0.537527	0.0143132	0.17				0.00105094	1.487E-10
rs2141277	A	G	A	G	0.00984668	0.0216605	0.4789	0.475795	0.0142703	0.11				0.00104744	5.436E-21
rs2142840	C	G	C	G	-0.0064146	-0.0106149	0.3145	0.313681	0.0153211	0.56	rs1574829	T	C	0.00112693	1.257E-08
rs2145265	T	C	T	C	0.010953	0.0031649	0.8408	0.844582	0.0195803	0.69				0.00143019	1.886E-14
rs2146820	T	C	T	C	0.00603878	0.0120973	0.4393	0.441583	0.0143279	0.34				0.00105431	1.02E-08
rs2147810	A	G	A	G	0.00628474	0.019785	0.2907	0.298318	0.0155452	0.1				0.00115248	4.944E-08
rs2160317	T	C	T	C	0.00664996	-0.0067035	0.4324	0.430129	0.0143432	0.57				0.0010562	3.058E-10
rs2160514	A	C	A	C	-0.0111099	0.00158595	0.5614	0.561385	0.0145146	0.77				0.00105449	5.901E-26

rs2172122	C	G	C	G	-0.0092452	0.0144919	0.361	0.371417	0.0147232	0.15				0.00108989	2.207E-17
rs2173108	T	C	T	C	0.00894438	0.0201586	0.136	0.133359	0.0209022	0.36				0.00152646	4.647E-09
rs2182918	T	G	T	G	0.00594936	-0.0528461	0.4911	0.481516	0.014224	0.00026				0.0010472	1.338E-08
rs2192639	C	G	C	G	-0.0110073	-0.0093081	0.1258	0.119382	0.0219137	0.64	rs4781320	T	G	0.00157785	3.039E-12
rs2195041	A	G	A	G	0.00659146	-0.0166297	0.4059	0.412315	0.0145039	0.21				0.00106555	6.164E-10
rs2195086	T	G	T	G	0.0124901	0.00421956	0.8447	0.836112	0.0192228	0.780001				0.00144469	5.368E-18
rs2202781	A	G	A	G	-0.0076803	-0.0056764	0.7686	0.776727	0.0172025	0.98				0.00124125	6.113E-10
rs2204506	A	G	A	G	0.0079013	0.00992442	0.5657	0.581575	0.014388	0.25				0.00105785	8.059E-14
rs2208030	T	C	T	C	0.0059117	0.00708663	0.4824	0.494094	0.0141963	0.64				0.00104758	1.67E-08
rs2210054	A	G	A	G	0.00850114	-0.0038849	0.5825	0.587487	0.0145216	0.96				0.00106105	1.127E-15
rs2210174	T	C	T	C	0.00612558	-0.0026586	0.6201	0.623826	0.0146654	0.89				0.00107807	1.328E-08
rs2214631	A	T	A	T	0.00706197	-0.0235943	0.2788	0.267024	0.0160308	0.11				0.00116691	1.432E-09
rs2216009	T	G	T	G	0.00620674	-0.0076472	0.4502	0.436277	0.0143412	0.53				0.00105174	3.597E-09
rs2223728	A	G	A	G	-0.0065174	0.00089317	0.3506	0.34544	0.0149961	0.75				0.0010966	2.798E-09
rs2223928	T	C	T	C	0.00652663	-0.0235749	0.549	0.547787	0.0144727	0.0810009				0.0010552	6.191E-10
rs2224427	A	G	A	G	-0.0072062	0.00384647	0.4712	0.490404	0.0142706	0.68				0.00104825	6.208E-12
rs2237316	T	G	T	G	0.00910798	0.0123879	0.2008	0.202673	0.0177171	0.29				0.00130618	3.108E-12
rs2237432	T	C	T	C	-0.007257	-0.0088472	0.7528	0.748149	0.0164262	0.47				0.00121296	2.188E-09
rs2238483	A	G	A	G	0.0100938	-0.0094755	0.8309	0.826075	0.0189709	0.719999				0.00139856	5.309E-13
rs2239736	T	C	T	C	0.00855693	-0.0162981	0.5098	0.517828	0.0142168	0.26				0.00104714	3.038E-16
rs2240471	A	G	A	G	0.00674211	0.0170672	0.4388	0.446344	0.0143248	0.11	rs10257510	T	C	0.00106143	2.13E-10
rs2240662	T	G	T	G	-0.0066205	-0.0115177	0.342	0.34511	0.0149298	0.25				0.00110974	2.43E-09
rs2241722	A	C	A	C	0.00757964	0.00408914	0.3233	0.326698	0.0151372	0.66				0.00112516	1.62E-11
rs225285	C	G	C	G	-0.0087602	0.00270249	0.729	0.741123	0.0161823	0.52				0.00117724	9.972E-14
rs2269506	T	C	T	C	0.0134335	0.00681572	0.1039	0.101729	0.0236114	0.9				0.00171944	5.588E-15
rs2271593	T	C	T	C	0.00965212	-0.000655	0.1533	0.153957	0.019672	1				0.00145237	3.018E-11
rs2273551	T	C	T	C	0.00853241	0.0275994	0.1754	0.173486	0.0188072	0.18				0.00137586	5.584E-10
rs2275154	A	G	A	G	0.015584	-0.0004689	0.6708	0.677131	0.015171	0.709999				0.00111349	1.663E-44
rs2279574	A	C	A	C	0.0105526	-0.0009374	0.5354	0.541372	0.0142164	0.84				0.00104923	8.532E-24
rs2283250	A	G	A	G	-0.0077742	0.00790761	0.4843	0.483375	0.0142778	0.55				0.00104702	1.126E-13
rs2288004	C	G	C	G	0.00926668	-0.0246045	0.3936	0.381679	0.0145906	0.0490004				0.00107118	5.117E-18
rs2289769	A	G	A	G	-0.008382	-0.0003883	0.8604	0.860669	0.0205139	1				0.0015098	2.831E-08
rs2293445	A	G	A	G	0.0118545	-0.0119485	0.384	0.387297	0.0145738	0.67	rs2293446	A	G	0.00107621	3.226E-28
rs2297293	C	G	C	G	0.00791431	0.0171213	0.3161	0.305596	0.0154351	0.25				0.00112834	2.316E-12
rs2298679	T	C	T	C	-0.0097912	-0.0080671	0.2051	0.209565	0.0174556	0.719999				0.00129607	4.211E-14
rs2301015	T	C	T	C	-0.0106495	-0.0169064	0.3267	0.335665	0.01503	0.21				0.00111581	1.37E-21
rs2301465	T	C	T	C	-0.0155932	0.0189728	0.0466	0.046729	0.0336727	0.68				0.00248246	3.355E-10
rs2303083	A	G	A	G	0.0133282	-0.0117695	0.1889	0.194081	0.0179606	0.57				0.00133695	2.084E-23
rs2303907	T	C	T	C	0.00852176	0.00099711	0.5239	0.517639	0.0141789	0.82				0.0010477	4.151E-16
rs2303929	A	G	A	G	0.0093103	-0.0264303	0.2329	0.227209	0.0169162	0.089				0.0012389	5.675E-14
rs2304282	A	G	A	G	0.011001	0.0238378	0.4148	0.406494	0.0144604	0.14				0.00106252	4.037E-25
rs2313547	T	C	T	C	0.00630311	0.0107861	0.5026	0.491766	0.0142298	0.44				0.00104662	1.721E-09
rs232464	T	C	T	C	0.00918058	-0.0104885	0.2226	0.227076	0.0169198	0.62				0.00125785	2.908E-13
rs2336723	T	C	T	C	0.00684103	-0.0095298	0.4327	0.437005	0.0142946	0.64				0.00105612	9.303E-11
rs2336866	A	C	A	C	-0.0079548	-0.0479504	0.8443	0.842844	0.0196235	0.00940005				0.00144477	3.668E-08
rs2338719	T	C	T	C	0.00839849	-0.0012977	0.3413	0.336531	0.0150229	0.88				0.00110424	2.83E-14
rs2342525	A	G	A	G	-0.0069398	0.025675	0.5803	0.589386	0.0144636	0.0769999				0.00106057	6.001E-11
rs2343522	T	G	T	G	-0.0081073	-0.0273355	0.1507	0.146418	0.0202399	0.15				0.00147163	3.6E-08
rs2345617	C	G	C	G	-0.0074307	0.0166031	0.2747	0.283292	0.0157829	0.17				0.00117241	2.329E-10
rs2362462	T	C	T	C	-0.0071746	0.0296437	0.6002	0.616223	0.0146922	0.0230001				0.00106848	1.883E-11
rs2364972	A	G	A	G	0.00719395	0.00373566	0.5435	0.536716	0.0142517	0.649999				0.00105121	7.72E-12
rs2371001	A	G	A	G	-0.0092875	0.0189921	0.5108	0.516448	0.0142085	0.23				0.00104675	7.151E-19
rs2413005	T	C	T	C	0.00812354	-0.0110527	0.3118	0.313957	0.0152919	0.62				0.00112958	6.394E-13
rs2421694	A	G	A	G	-0.0083387	0.0121037	0.5686	0.576347	0.0143612	0.23				0.00105673	2.99E-15
rs2424637	A	G	A	G	0.0073151	-0.0144043	0.6191	0.618909	0.0148129	0.450001				0.00107783	1.148E-11
rs2428373	T	C	T	C	-0.0083229	0.0262419	0.1466	0.152306	0.0204033	0.18				0.00150167	2.984E-08

rs2438390	A	G	A	G	-0.0092877	-0.0114538	0.1607	0.162136	0.0192643	0.51				0.00142496	7.12E-11
rs2441019	A	T	A	T	-0.0102206	0.0269594	0.687	0.688762	0.0153189	0.0779992				0.00112854	1.35E-19
rs2447091	T	C	T	C	0.00912078	0.0203746	0.6112	0.610125	0.0145724	0.18				0.00107641	2.388E-17
rs2448929	T	C	T	C	0.00685473	-0.0041217	0.5185	0.524667	0.0142945	0.780001				0.0010477	6.043E-11
rs2456523	T	G	T	G	0.00739876	-0.0244973	0.7463	0.739114	0.0162043	0.16	rs2456531	C	A	0.00120269	7.642E-10
rs2459338	A	G	A	G	0.00770696	-0.0008705	0.484	0.495831	0.0142223	0.99				0.00104704	1.826E-13
rs2473351	T	C	T	C	0.00884587	-0.0233343	0.7551	0.752442	0.0165963	0.12				0.00121729	3.67E-13
rs2477674	A	T	A	T	-0.0099889	-0.0025065	0.3236	0.325216	0.0152592	0.58				0.0011258	7.119E-19
rs2481061	A	G	A	G	0.00627683	-0.0238989	0.4162	0.411501	0.0145089	0.0710003				0.00106166	3.376E-09
rs2488697	T	C	T	C	-0.0095262	0.0128715	0.2711	0.273696	0.0159744	0.51				0.00117725	5.882E-16
rs2491365	T	C	T	C	0.0158832	-0.0095385	0.7297	0.724513	0.0159232	0.35				0.00117835	2.075E-41
rs2498018	C	G	C	G	-0.0089306	0.0141097	0.1912	0.19679	0.0179807	0.55				0.0013306	1.922E-11
rs2504846	T	C	T	C	-0.0257931	0.00574583	0.0262	0.03037	0.0414684	0.68				0.00328026	3.743E-15
rs2517063	A	C	A	C	0.00708615	0.0243267	0.5438	0.540326	0.0142851	0.0870001				0.00105372	1.761E-11
rs252991	A	G	A	G	0.0102574	-0.0173495	0.375	0.370424	0.0146969	0.29	rs3843490	C	T	0.00108097	2.333E-21
rs2535692	A	G	A	G	0.00686048	-0.0076554	0.2731	0.266113	0.0160361	0.49				0.00117439	5.17E-09
rs2542673	A	C	A	C	0.00773671	-0.0216029	0.3222	0.324509	0.0151767	0.17				0.00112276	5.545E-12
rs2550218	A	G	A	G	0.00706266	-0.0294435	0.4125	0.417021	0.0145307	0.032				0.00106335	3.089E-11
rs2552505	T	C	T	C	0.00619365	-0.0243532	0.6513	0.665983	0.0151113	0.14	rs2552490	A	C	0.00109829	1.705E-08
rs2555506	A	G	A	G	0.00925845	-0.0277896	0.1769	0.178259	0.0185627	0.13				0.00137961	1.935E-11
rs2561477	A	G	A	G	0.00990309	-0.0301767	0.3171	0.322404	0.0151885	0.0710003				0.00112459	1.301E-18
rs2565059	A	G	A	G	0.00892604	-0.0054636	0.1998	0.195308	0.0179531	0.649999				0.0013095	9.332E-12
rs2567609	T	C	T	C	-0.0064976	-0.0009511	0.547	0.551278	0.0142624	1				0.00105116	6.348E-10
rs2570492	A	G	A	G	0.012994	5.8784E-06	0.3964	0.392356	0.0145936	0.95				0.00106972	5.948E-34
rs2584240	A	T	A	T	-0.0113465	0.00176974	0.8912	0.894162	0.0232002	0.98				0.00168162	1.506E-11
rs258696	C	G	C	G	0.00730626	-0.006321	0.7318	0.732457	0.0160433	0.760001				0.0011811	6.158E-10
rs2591114	A	G	A	G	-0.0080543	0.00750497	0.5455	0.547146	0.0142795	0.7				0.00105116	1.822E-14
rs2593017	A	T	A	T	-0.0124062	-0.0146809	0.8487	0.841175	0.0195211	0.33				0.0014604	1.982E-17
rs2610990	A	G	A	G	0.0115793	-0.0067776	0.2583	0.260003	0.0161714	0.83				0.00119546	3.461E-22
rs2614463	A	T	A	T	-0.0099034	-0.0277877	0.5874	0.580154	0.0144161	0.017				0.00106418	1.322E-20
rs2618039	A	T	A	T	0.0106432	-0.0022232	0.6105	0.618524	0.0146282	0.93				0.00107314	3.489E-23
rs2624841	T	C	T	C	-0.0264476	0.0186385	0.3383	0.340008	0.0149879	0.23				0.00110629	2.62E-126
rs2630767	T	C	T	C	0.013004	0.00351941	0.4413	0.436955	0.0143062	0.86				0.00105498	6.555E-35
rs2631535	A	G	A	G	0.0122965	-0.0107214	0.3289	0.316806	0.0152508	0.37				0.00111375	2.438E-28
rs2632667	A	G	A	G	-0.0061806	-0.020025	0.5062	0.505407	0.0142779	0.11				0.00104909	3.835E-09
rs2633723	T	C	T	C	0.00872976	0.00412772	0.6118	0.605486	0.0145346	0.780001				0.00107399	4.355E-16
rs2634436	T	C	T	C	0.00698359	-0.0226844	0.25	0.242614	0.0166967	0.13				0.00120895	7.607E-09
rs2637609	A	G	A	G	0.00808334	-0.0075123	0.738	0.731051	0.0159841	0.69				0.00119007	1.105E-11
rs2639655	C	G	C	G	0.0092191	-0.0393171	0.8065	0.815085	0.0183876	0.0439997				0.00132565	3.537E-12
rs2653344	T	C	T	C	-0.0085787	-0.0254027	0.2016	0.212961	0.0173506	0.2				0.00130424	4.777E-11
rs265981	A	G	A	G	0.007221	0.00418515	0.3796	0.384051	0.0145837	0.49				0.00107838	2.144E-11
rs2668196	A	T	A	T	-0.011857	0.00304455	0.1939	0.191236	0.0180824	0.91				0.00132351	3.28E-19
rs2669890	A	T	A	T	0.00934719	-0.0039644	0.2176	0.207914	0.0175016	0.8				0.00126814	1.695E-13
rs267152	A	C	A	C	-0.0089459	0.0314358	0.8618	0.862584	0.0205817	0.14				0.00152814	4.79E-09
rs2676507	A	G	A	G	0.00825973	-0.0161114	0.3179	0.321273	0.0152192	0.36				0.00112368	1.971E-13
rs268134	A	G	A	G	0.00931409	-0.0148361	0.2483	0.249021	0.0164006	0.31				0.00121116	1.465E-14
rs2702565	T	C	T	C	0.00603308	-0.017301	0.6129	0.620528	0.0146568	0.22				0.00107425	1.954E-08
rs2706762	T	C	T	C	0.00821664	-0.0205482	0.1478	0.14959	0.0198675	0.29				0.00148043	2.85E-08
rs2710873	A	G	A	G	-0.0113418	0.0357374	0.1607	0.163327	0.0193532	0.0359998				0.00147231	1.328E-14
rs2729790	T	C	T	C	0.0063537	0.00683782	0.3759	0.366113	0.014775	0.51				0.00108076	4.132E-09
rs2729987	T	C	T	C	0.00747282	0.00514687	0.7863	0.783424	0.0173735	0.74				0.00127701	4.856E-09
rs273028	A	T	A	T	-0.0135331	0.00450621	0.0751	0.070834	0.0278096	0.84				0.00198539	9.317E-12
rs2731034	A	G	A	G	0.00601408	0.00935837	0.5959	0.591365	0.0144612	0.41				0.0010663	1.702E-08
rs2736752	T	G	T	G	0.00904727	-0.0201557	0.784	0.788002	0.0173566	0.26				0.00127479	1.271E-12
rs2742690	A	C	A	C	-0.0086062	0.0358295	0.1999	0.207809	0.0176852	0.0379997				0.00131012	5.07E-11
rs2750097	A	G	A	G	-0.0073479	0.0173381	0.5883	0.58488	0.0144283	0.24				0.00106322	4.802E-12

rs276105	T	G	T	G	0.00586996	-0.0103449	0.4558	0.463018	0.0142593	0.4				0.00105379	2.547E-08
rs2764684	T	C	T	C	0.0150109	-0.0047419	0.8279	0.821364	0.0186185	0.68				0.00138678	2.639E-27
rs2795022	T	C	T	C	0.00581376	0.0131074	0.4042	0.41002	0.0144265	0.31				0.00106626	4.965E-08
rs2824999	T	C	T	C	-0.0090232	0.0118911	0.7304	0.728743	0.0160191	0.73				0.00117916	1.977E-14
rs283272	T	C	T	C	-0.0092296	-0.0325439	0.3179	0.31893	0.0152973	0.025				0.00112412	2.2E-16
rs28360512	A	G	A	G	-0.0091383	-0.0018043	0.35	0.350062	0.0149109	0.79				0.00109735	8.253E-17
rs28381527	A	G	A	G	-0.0199902	0.0376334	0.0784	0.078928	0.0263161	0.1				0.00194663	9.736E-25
rs28415206	T	G	T	G	0.00781658	-0.0086721	0.7981	0.799247	0.0178005	0.630001				0.00130363	2.025E-09
rs28438078	A	G	A	G	-0.009642	-0.0010633	0.1584	0.166698	0.0191516	0.86				0.00143312	1.723E-11
rs28448822	A	C	A	C	0.00984516	0.02008	0.8692	0.868306	0.0209788	0.41				0.00155185	2.239E-10
rs28458909	T	C	T	C	-0.0190198	0.00755704	0.1235	0.122421	0.0216116	0.760001				0.00164419	5.978E-31
rs28469453	C	G	C	G	-0.0061843	0.0144217	0.3638	0.378255	0.0147196	0.36				0.00109153	1.466E-08
rs28473320	T	C	T	C	-0.0071486	0.0277435	0.6969	0.69922	0.0155444	0.0739997				0.00113947	3.52E-10
rs2848780	A	G	A	G	-0.00808	-0.0097109	0.2717	0.277183	0.0159469	0.47				0.00117628	6.464E-12
rs28542042	T	C	T	C	0.00660804	-0.0060009	0.2995	0.308887	0.0155052	0.6				0.00114317	7.463E-09
rs2856327	T	C	T	C	-0.0091895	-0.0184362	0.154	0.16218	0.0192284	0.39				0.0015685	4.657E-09
rs28588750	A	G	A	G	0.0114613	0.00082843	0.6402	0.633302	0.0147557	1				0.00109024	7.584E-26
rs28589407	T	C	T	C	0.0202202	-0.0595974	0.9772	0.97961	0.0505164	0.35				0.00351074	8.451E-09
rs28594971	A	G	A	G	0.00870002	0.0177211	0.179	0.174986	0.0186768	0.37				0.0013655	1.877E-10
rs28603130	C	G	C	G	0.00861003	0.011335	0.7492	0.743337	0.0162609	0.44				0.00120712	9.865E-13
rs28607949	T	C	T	C	-0.0081954	0.0132502	0.4539	0.453382	0.0143634	0.31	rs380322	T	C	0.00105811	9.514E-15
rs28610468	A	T	A	T	-0.0088824	-0.0017974	0.2048	0.204432	0.0176189	0.81				0.00129661	7.373E-12
rs28622594	A	G	A	G	-0.0062192	-0.0048745	0.4422	0.441291	0.0142993	0.760001				0.00105357	3.566E-09
rs28622958	A	C	A	C	-0.0057763	-0.0110508	0.4649	0.467782	0.0142277	0.5				0.0010491	3.674E-08
rs28661002	T	C	T	C	0.0127997	0.00700408	0.757	0.765188	0.0168528	0.47				0.00122016	9.561E-26
rs2867932	A	G	A	G	0.00714813	0.0133511	0.3907	0.399017	0.0144953	0.23				0.00107259	2.653E-11
rs28711500	A	C	A	C	0.00748525	0.00555705	0.3025	0.308776	0.0154966	0.8				0.00113961	5.092E-11
rs28737363	A	C	A	C	-0.0068761	0.00073585	0.3004	0.297	0.0155571	0.96	rs57349056	C	T	0.00115065	2.289E-09
rs28741121	A	G	A	G	0.0106031	-0.0252859	0.166	0.171442	0.0190185	0.35				0.00141472	6.641E-14
rs28807191	T	C	T	C	-0.007239	0.0168264	0.5588	0.570491	0.0143738	0.15				0.00105421	6.564E-12
rs28807201	T	C	T	C	-0.0113948	0.00402342	0.6998	0.711394	0.0156827	0.55				0.0011424	1.966E-23
rs2884364	A	G	A	G	-0.0159498	0.0196686	0.3818	0.382927	0.0146339	0.11				0.00107703	1.286E-49
rs2886405	T	G	T	G	0.00834816	0.00083342	0.3604	0.357323	0.0148732	1				0.00109672	2.707E-14
rs2901785	A	G	A	G	0.0141211	0.0125069	0.4485	0.450966	0.0142639	0.32				0.0010521	4.523E-41
rs2911653	A	C	A	C	0.00626755	0.0152355	0.6279	0.635887	0.0150437	0.2				0.00108252	7.058E-09
rs2916490	A	G	A	G	-0.0104226	0.0550718	0.3069	0.301843	0.0156833	0.00039				0.00113453	4.044E-20
rs2919151	T	G	T	G	0.00869276	0.00595097	0.4865	0.47949	0.0142974	0.719999				0.00104864	1.135E-16
rs2934510	T	C	T	C	0.00738558	-0.0218425	0.2309	0.230082	0.016953	0.14				0.00124203	2.747E-09
rs2939261	C	G	C	G	0.0115023	0.00040413	0.788	0.782302	0.0171999	0.88				0.00128038	2.627E-19
rs2941318	A	G	A	G	0.00821406	-0.0388482	0.4299	0.426391	0.0143832	0.00729995				0.00105724	7.911E-15
rs2958942	T	C	T	C	0.00702679	-0.0263752	0.7459	0.745148	0.0163427	0.0940005				0.00120239	5.093E-09
rs2964252	A	G	A	G	0.010242	0.0160567	0.3111	0.316564	0.0152864	0.17				0.00113042	1.298E-19
rs2974337	T	C	T	C	0.0118039	0.00355303	0.4942	0.499822	0.0141804	0.61				0.0010496	2.416E-29
rs2977467	T	C	T	C	-0.0118535	-0.0064623	0.7868	0.779301	0.0172241	0.62				0.00128552	2.938E-20
rs2999158	T	C	T	C	0.00837216	-0.0087017	0.3324	0.334577	0.01504	0.46				0.00111108	4.886E-14
rs3008032	A	G	A	G	0.00717328	-0.0173246	0.2014	0.19819	0.0179484	0.29				0.00130779	4.132E-08
rs3013170	T	C	T	C	0.00707139	-0.0211273	0.6063	0.604119	0.0145439	0.1				0.00107381	4.528E-11
rs303752	A	G	A	G	-0.0060412	0.0219597	0.4049	0.408396	0.0146374	0.2				0.00106642	1.472E-08
rs305191	T	C	T	C	0.00906586	-0.0197351	0.2675	0.274849	0.0159015	0.21				0.00118246	1.763E-14
rs307913	A	G	A	G	0.00928996	-0.0141273	0.8508	0.851793	0.0204134	0.49				0.00147679	3.158E-10
rs3095075	A	G	A	G	-0.0136006	0.0190099	0.5548	0.554679	0.0143743	0.17				0.0010533	3.812E-38
rs3097862	A	G	A	G	-0.0060424	0.0140837	0.4054	0.40236	0.0144821	0.31				0.0010659	1.436E-08
rs3098650	C	G	C	G	0.0118136	-0.0047623	0.6237	0.637202	0.0147673	0.8				0.00108018	7.71E-28
rs3099082	T	G	T	G	0.0070392	0.0291385	0.5673	0.562499	0.0143676	0.0479999				0.00105612	2.639E-11
rs3106836	A	G	A	G	-0.0062251	-0.0123126	0.3861	0.399693	0.0144879	0.34				0.00107476	6.936E-09
rs3108680	T	C	T	C	0.0101964	-0.0251402	0.6531	0.64926	0.0149007	0.0439997				0.00110006	1.888E-20

rs3111336	T	G	T	G	-0.0068562	0.00951913	0.3228	0.315552	0.0152941	0.46				0.00111915	9.012E-10
rs3112178	A	G	A	G	-0.0069665	-0.0174372	0.2514	0.256784	0.0163763	0.37				0.00120616	7.66E-09
rs3121984	T	C	T	C	0.00956813	0.0242917	0.69	0.701356	0.015508	0.11				0.00113138	2.746E-17
rs3134530	A	C	A	C	0.00689868	0.00770028	0.3393	0.359501	0.014777	0.780001	rs753488	A	G	0.00110758	4.7E-10
rs321250	T	G	T	G	-0.0088313	-0.0074249	0.4225	0.407034	0.0144597	0.41				0.00105931	7.635E-17
rs322547	A	G	A	G	-0.0078749	0.0149969	0.273	0.266244	0.0161656	0.37				0.00117453	2.013E-11
rs322627	C	G	C	G	-0.0100006	-0.0126195	0.3777	0.383149	0.0146089	0.39				0.00107939	1.956E-20
rs329120	T	C	T	C	0.0132651	0.00217646	0.4245	0.419323	0.0143956	0.69				0.00105878	5.213E-36
rs336433	A	G	A	G	0.00774529	-0.0246959	0.4398	0.451706	0.0143013	0.0790005				0.00105447	2.054E-13
rs34124911	T	C	T	C	-0.0064295	-0.0183863	0.5886	0.594385	0.0144541	0.25				0.0010698	1.855E-09
rs34189321	A	T	A	T	0.0147459	-0.0216592	0.8736	0.871008	0.0212055	0.15				0.00157509	7.838E-21
rs34305371	A	G	A	G	0.0314328	-0.0113378	0.0993	0.101326	0.0235066	0.719999				0.00175826	1.768E-71
rs34316	A	C	A	C	0.0162889	0.0161348	0.4285	0.419484	0.0144326	0.22				0.00106395	6.577E-53
rs34353826	A	T	A	T	0.00769301	0.0340004	0.1726	0.170021	0.0189854	0.0949992				0.00138939	3.072E-08
rs34408666	A	G	A	G	-0.0093235	0.00496541	0.1399	0.130853	0.021114	1				0.0015089	6.464E-10
rs34488670	T	C	T	C	0.0140229	-0.0234992	0.7882	0.788874	0.0174517	0.18				0.00128082	6.793E-28
rs34591327	C	G	C	G	0.014208	-0.0250399	0.1234	0.118759	0.0220145	0.18				0.00160062	6.905E-19
rs34632631	T	C	T	C	-0.0184117	0.0322204	0.0307	0.029479	0.0423978	0.54				0.00306985	1.998E-09
rs34719425	T	C	T	C	0.00950644	-0.0289038	0.243	0.249564	0.0164017	0.0680002				0.00122408	8.074E-15
rs34743418	T	C	T	C	-0.0099871	0.0099254	0.4367	0.429769	0.0143341	0.68				0.00106142	5.014E-21
rs34762508	T	C	T	C	0.00671382	0.0104327	0.4245	0.44233	0.0142789	0.35				0.00105878	2.28E-10
rs348036	T	G	T	G	-0.007991	-0.0005733	0.1936	0.191696	0.0180067	0.87				0.00133307	2.045E-09
rs34885384	T	C	T	C	-0.011861	0.0286225	0.8301	0.828092	0.018986	0.16				0.00139852	2.235E-17
rs34943464	A	G	A	G	-0.0063456	-0.0142647	0.3697	0.32961	0.015191	0.32	rs10183538	T	C	0.00108396	4.802E-09
rs34959108	A	G	A	G	-0.0071119	0.0181552	0.7663	0.772783	0.0169286	0.48				0.00123647	8.84E-09
rs34961029	T	G	T	G	-0.0065973	-0.0082421	0.262	0.267199	0.0160614	0.58				0.00119084	3.03E-08
rs34967558	T	C	T	C	-0.0100957	0.00169891	0.764	0.765607	0.0168378	0.97				0.00123228	2.557E-16
rs34980782	T	C	T	C	0.00916846	-0.0381083	0.8894	0.884931	0.0224907	0.0860003				0.00167343	4.29E-08
rs34984805	T	C	T	C	0.00740438	0.00592734	0.5151	0.510194	0.0142072	0.55				0.00104708	1.536E-12
rs35039660	T	C	T	C	-0.0135806	0.0312727	0.9457	0.939116	0.0298387	0.34				0.00232322	5.058E-09
rs35041900	T	C	T	C	-0.015522	-0.0071946	0.0907	0.092517	0.0245614	0.97				0.00182203	1.608E-17
rs35079242	T	G	T	G	-0.0093133	0.00759619	0.132	0.129097	0.0213793	0.760001				0.00154584	1.697E-09
rs35111506	C	G	C	G	-0.0144576	0.0105109	0.9461	0.942727	0.0306326	0.91				0.00231855	4.497E-10
rs35111947	A	G	A	G	0.00693626	-0.0238657	0.6604	0.670441	0.0152065	0.26				0.00110522	3.477E-10
rs35170529	T	C	T	C	-0.0098085	-0.0308772	0.8861	0.884484	0.0225294	0.0739997				0.0016482	2.665E-09
rs35181444	T	C	T	C	0.00861774	-0.0105301	0.258	0.259488	0.0162909	0.64				0.00119882	6.537E-13
rs35209304	T	C	T	C	0.00998886	-0.0483126	0.1915	0.183949	0.0183635	0.0015				0.00133098	6.164E-14
rs35226705	A	C	A	C	0.00614474	0.0166378	0.5358	0.540024	0.014227	0.26				0.00105006	4.862E-09
rs35271256	A	G	A	G	0.0131367	0.00411638	0.836	0.835427	0.0192687	0.94				0.00141315	1.452E-20
rs35316276	T	C	T	C	0.010525	-0.0236129	0.2846	0.275528	0.0158623	0.0749998				0.00116525	1.685E-19
rs35319653	T	C	T	C	0.0147005	-0.0292684	0.3396	0.342727	0.0149749	0.0490004				0.0011049	2.163E-40
rs35345466	A	T	A	T	0.00732264	-0.0285525	0.2641	0.259379	0.0162535	0.0769999	rs11598785	G	A	0.00118807	7.129E-10
rs35359254	C	G	C	G	0.0113883	0.00926323	0.9038	0.902224	0.0243066	0.56				0.00178094	1.612E-10
rs35372440	A	T	A	T	0.0072661	0.00231229	0.8085	0.792932	0.0177699	0.86				0.00133035	4.714E-08
rs35375125	T	C	T	C	-0.0094599	-0.0167563	0.6453	0.62908	0.0147149	0.11				0.00109371	5.192E-18
rs35414043	A	G	A	G	-0.0153931	0.0425401	0.0764	0.07244	0.0273711	0.089				0.00197007	5.576E-15
rs35414759	T	C	T	C	-0.0109385	0.0207784	0.8149	0.805003	0.0179438	0.37				0.00134728	4.693E-16
rs35445341	A	G	A	G	0.00579432	-0.0002823	0.4522	0.441342	0.0145276	0.74				0.00105594	4.078E-08
rs35452994	A	G	A	G	-0.0091242	0.00221137	0.1957	0.196806	0.0178731	0.89				0.00133796	9.163E-12
rs35553446	C	G	C	G	0.00723874	0.00238454	0.2151	0.217797	0.017199	0.88				0.00127346	1.313E-08
rs35564420	T	C	T	C	-0.0070404	-0.0111698	0.7645	0.765876	0.0167559	0.42				0.00123318	1.134E-08
rs35567946	C	G	C	G	-0.0098595	0.00200148	0.5908	0.582491	0.0143963	0.89				0.00106434	1.973E-20
rs35583726	A	G	A	G	0.0118542	0.00412324	0.8334	0.83163	0.0189644	0.93				0.00140426	3.131E-17
rs35625885	A	G	A	G	0.00917352	-0.004881	0.8745	0.875242	0.0216559	0.79				0.00158126	6.572E-09
rs35657180	A	T	A	T	0.00780225	0.017649	0.226	0.220318	0.0172539	0.39				0.00125923	5.783E-10
rs35683183	A	G	A	G	0.00874894	-0.0053988	0.2136	0.207595	0.0175954	0.649999				0.00128162	8.711E-12

rs356903	T	C	T	C	-0.007191	-0.0198481	0.2517	0.239798	0.016613	0.17	rs356862	C	A	0.00120568	2.457E-09
rs35693868	T	C	T	C	0.00741322	-0.0154681	0.2552	0.251014	0.0163774	0.23				0.0012002	6.561E-10
rs35700858	A	C	A	C	0.00900009	0.0109291	0.163	0.169023	0.0189897	0.42				0.00142649	2.804E-10
rs35711462	A	G	A	G	0.00572772	0.0238648	0.4926	0.488789	0.014233	0.0470002				0.00104676	4.456E-08
rs35733856	A	G	A	G	-0.0118819	-0.0089314	0.5961	0.600232	0.0145195	0.54				0.00106683	8.256E-29
rs35739581	T	C	T	C	0.0129609	0.0233107	0.9179	0.919349	0.026094	0.34				0.00196007	3.776E-11
rs35742190	T	G	T	G	0.00734737	-0.0182736	0.4867	0.492808	0.0142215	0.2				0.00104914	2.498E-12
rs35754740	T	C	T	C	0.00651514	0.00026486	0.5786	0.574758	0.0144011	0.81				0.00106012	7.948E-10
rs35759537	T	C	T	C	0.00755153	0.0274081	0.187	0.194854	0.0178841	0.16				0.00134537	1.987E-08
rs35769536	A	G	A	G	0.00853235	0.00165173	0.8486	0.844034	0.0196286	0.98				0.00145981	5.066E-09
rs35797585	A	G	A	G	-0.0101612	0.0103452	0.2448	0.243475	0.0167835	0.54	rs61692722	C	G	0.00122532	1.106E-16
rs35878043	T	G	T	G	-0.0083425	0.00067615	0.1431	0.149492	0.0202305	0.88				0.00149996	2.669E-08
rs359233	A	G	A	G	0.0102384	-0.0088015	0.3738	0.370197	0.0147477	0.49				0.00108152	2.883E-21
rs35951883	A	G	A	G	-0.0080504	-0.0054347	0.7794	0.784291	0.0172567	0.709999				0.00127034	2.334E-10
rs36035373	A	G	A	G	-0.0244003	0.0151359	0.0146	0.014886	0.0585877	0.94				0.00444161	3.936E-08
rs36048136	A	G	A	G	0.0117873	0.0200634	0.2269	0.221968	0.0170734	0.22				0.00125192	4.696E-21
rs36116433	T	C	T	C	-0.0069843	-0.0160647	0.4525	0.445365	0.0143345	0.29				0.0010534	3.358E-11
rs36120534	T	C	T	C	0.0093836	0.0265268	0.1819	0.188751	0.0182271	0.15				0.00135642	4.591E-12
rs36142021	T	C	T	C	-0.0120691	-0.0051652	0.3024	0.284744	0.0157587	0.7				0.00113957	3.274E-26
rs3731507	T	C	T	C	0.0275792	0.00739656	0.0438	0.047273	0.0335093	0.61				0.00258907	1.708E-26
rs3740484	T	G	T	G	0.00960386	-0.0009549	0.3153	0.330301	0.0150649	0.83				0.00112631	1.506E-17
rs3747631	C	G	C	G	0.0204311	0.0234903	0.2148	0.209268	0.0174288	0.17				0.0012741	7.165E-58
rs3748328	T	C	T	C	0.0061131	0.0239289	0.3293	0.33337	0.0150469	0.0649995				0.0011134	4.005E-08
rs3751667	T	C	T	C	-0.0088902	0.00705153	0.2291	0.232355	0.0169125	0.59				0.00124898	1.095E-12
rs3759344	A	G	A	G	-0.0098423	0.0442902	0.1002	0.105517	0.0231436	0.0269998				0.00174432	1.678E-08
rs3768992	A	G	A	G	0.00659983	-0.0153872	0.5085	0.522281	0.0142788	0.35				0.00104695	2.902E-10
rs3770772	T	C	T	C	0.00750145	-0.0006948	0.7452	0.749114	0.0164531	0.67				0.0012033	4.549E-10
rs378342	T	C	T	C	-0.0081493	0.00210603	0.2675	0.264055	0.0160915	0.99				0.00118223	5.444E-12
rs3788337	A	G	A	G	0.00675177	-0.0114976	0.3666	0.354147	0.0148608	0.2				0.00108602	5.071E-10
rs37903	A	G	A	G	0.0109628	-0.0233765	0.8601	0.856305	0.0205626	0.3				0.00150844	3.665E-13
rs3796432	T	G	T	G	0.00684095	-0.0140138	0.3714	0.367186	0.0147778	0.38				0.00108294	2.67E-10
rs3811038	T	C	T	C	0.00950087	-0.0133807	0.7214	0.723858	0.0159644	0.64				0.00116764	4.05E-16
rs3817741	A	G	A	G	-0.0107773	-0.038575	0.1867	0.194075	0.0179219	0.0280001				0.00134281	1.009E-15
rs3817923	A	G	A	G	-0.0112783	0.0194692	0.1132	0.116421	0.0223359	0.51				0.00165196	8.667E-12
rs382196	T	G	T	G	-0.0070273	0.00945066	0.3772	0.382354	0.0146505	0.58				0.00107957	7.531E-11
rs3824992	A	G	A	G	-0.0058892	0.00033576	0.4027	0.396442	0.014562	0.88				0.0010672	3.414E-08
rs3844013	T	C	T	C	0.0145324	-0.0295284	0.9457	0.946684	0.0317428	0.31				0.00230988	3.147E-10
rs3863241	T	C	T	C	-0.0085901	0.00957637	0.5411	0.527769	0.0142105	0.630001				0.0010502	2.856E-16
rs3910664	T	C	T	C	-0.005884	-0.00439	0.6016	0.606727	0.0145256	0.97				0.00106881	3.688E-08
rs3922612	T	C	T	C	0.0101027	0.0172074	0.0937	0.087292	0.0251473	0.56				0.00179642	1.869E-08
rs3936100	T	G	T	G	0.00822656	0.0148481	0.3674	0.369319	0.0147256	0.32				0.00108762	3.907E-14
rs3948593	T	C	T	C	-0.0096651	0.0195682	0.1628	0.163146	0.019404	0.42				0.00141889	9.649E-12
rs4076457	T	C	T	C	0.0122215	0.00361139	0.2514	0.257367	0.0162527	0.96				0.00120753	4.468E-24
rs4082337	T	C	T	C	0.00877507	-0.0025504	0.6867	0.681703	0.0152591	0.760001				0.00113512	1.073E-14
rs41119	T	G	T	G	0.00600214	0.00909589	0.3577	0.353462	0.0150539	0.42				0.00109196	3.865E-08
rs4127499	A	G	A	G	0.0109595	0.0312606	0.345	0.356611	0.0148047	0.021				0.00110304	2.902E-23
rs41322948	A	G	A	G	0.0060852	-0.014536	0.357	0.357902	0.0148267	0.24				0.00109213	2.521E-08
rs4140762	A	G	A	G	0.00730111	0.0002843	0.5207	0.511741	0.014266	0.96				0.00104741	3.155E-12
rs4142949	A	C	A	C	0.0105629	0.0246447	0.5269	0.530851	0.0142419	0.0819993				0.00104832	7.056E-24
rs41741	T	G	T	G	0.00737479	0.00235463	0.6694	0.673273	0.0151497	0.66				0.00111229	3.353E-11
rs419438	T	G	T	G	-0.0071005	-0.0013412	0.5395	0.529142	0.0142621	0.88				0.00105089	1.411E-11
rs421154	C	G	C	G	0.0109765	0.0108847	0.7745	0.784026	0.0172957	0.41				0.00125388	2.062E-18
rs42210	C	G	C	G	-0.0117682	0.011749	0.7123	0.710776	0.0157518	0.36				0.00115875	3.129E-24
rs424387	C	G	C	G	-0.0061683	-0.0115326	0.5933	0.583966	0.0144438	0.47				0.00106552	7.093E-09
rs4262652	A	G	A	G	-0.0080502	0.00428376	0.6331	0.643565	0.0148512	0.73				0.00109068	1.572E-13
rs4272628	A	G	A	G	-0.0076477	-0.0078641	0.525	0.523444	0.014269	0.53				0.00104782	2.9E-13

rs4283969	T	G	T	G	0.011122	-0.00363	0.6436	0.634873	0.0148555	0.54				0.00109253	2.444E-24
rs4297478	T	C	T	C	-0.009218	0.00300121	0.5509	0.543859	0.0142735	0.74				0.00105197	1.906E-18
rs4319543	A	C	A	C	-0.0068071	-0.0215929	0.5212	0.513027	0.0142356	0.11				0.00104787	8.256E-11
rs4341632	A	G	A	G	0.0116989	0.0267761	0.1225	0.126155	0.0217055	0.24				0.00159661	2.346E-13
rs4347543	T	C	T	C	-0.0063279	-0.0155454	0.6274	0.622123	0.0146178	0.25				0.00108223	5.004E-09
rs4358081	A	C	A	C	-0.009704	0.00099769	0.531	0.52352	0.0142002	0.89				0.00104852	2.136E-20
rs4376462	T	G	T	G	-0.0083481	-0.0213643	0.3977	0.40655	0.014434	0.11				0.00106926	5.837E-15
rs4378243	T	G	T	G	0.0162224	-0.0209331	0.8392	0.834825	0.0191228	0.2				0.00142441	4.747E-30
rs4381456	T	C	T	C	0.00620822	-0.0023318	0.6386	0.646915	0.0148114	0.760001				0.00108964	1.217E-08
rs438788	A	T	A	T	0.00847396	0.0180911	0.2067	0.206778	0.017877	0.32				0.00130415	8.166E-11
rs4391653	T	C	T	C	-0.0081619	0.0211566	0.2621	0.266989	0.0160573	0.13				0.00118982	6.888E-12
rs4414947	T	C	T	C	0.00625531	-0.0103262	0.5846	0.580954	0.0143741	0.58	rs9994056	G	A	0.00107052	5.129E-09
rs444944	A	G	A	G	-0.0077847	-0.0066904	0.4692	0.461658	0.0142581	0.67				0.00105097	1.292E-13
rs4456284	T	C	T	C	-0.0099994	0.0109002	0.1253	0.123606	0.0216499	0.54				0.00158099	2.53E-10
rs4467547	T	G	T	G	0.0132237	0.0162006	0.4053	0.411761	0.0144556	0.23				0.00106831	3.439E-35
rs4483480	T	C	T	C	-0.0076832	-0.0032071	0.4256	0.428006	0.0143511	0.81				0.00105843	3.904E-13
rs4484264	A	G	A	G	0.0086422	0.0345536	0.1601	0.157869	0.019532	0.0619998				0.00142693	1.392E-09
rs4493405	A	G	A	G	0.00734481	-0.0131624	0.5317	0.517283	0.0142532	0.34				0.00104901	2.534E-12
rs4522099	C	G	C	G	0.0129085	-0.0542481	0.9314	0.928329	0.0276504	0.0359998				0.00207116	4.598E-10
rs4551987	A	G	A	G	0.0124831	-0.0135367	0.8372	0.842545	0.0195195	0.33				0.00142169	1.624E-18
rs4553692	A	G	A	G	0.0112065	-0.0019007	0.3714	0.367661	0.0147281	0.84				0.00108294	4.247E-25
rs4554425	T	C	T	C	0.00701761	-0.0002966	0.4864	0.497609	0.014196	0.95				0.00104703	2.053E-11
rs45570933	A	G	A	G	0.0118981	0.00917029	0.0713	0.073796	0.0273342	0.649999				0.00204571	6.013E-09
rs4557790	A	C	A	C	0.0204899	0.0194364	0.5537	0.558548	0.0143068	0.089				0.00105491	4.879E-84
rs4559697	A	G	A	G	-0.006658	-0.0026924	0.2926	0.309688	0.0153711	0.709999	rs4420280	A	C	0.0011512	7.307E-09
rs4561398	T	C	T	C	-0.0088686	0.0282058	0.485	0.486056	0.014253	0.0739997				0.00104751	2.527E-17
rs4583487	T	G	T	G	0.0229768	-0.0250268	0.3961	0.400973	0.014475	0.0879995				0.00106986	2.58E-102
rs4586770	T	C	T	C	-0.0079935	0.00628122	0.3983	0.390604	0.0145649	0.89	rs9848224	C	T	0.00106885	7.496E-14
rs4588749	A	G	A	G	0.0125645	-0.015353	0.4464	0.447813	0.0142933	0.36				0.00105257	7.59E-33
rs4597364	T	C	T	C	-0.0068548	-0.0109243	0.6643	0.663234	0.0152341	0.49				0.00111271	7.25E-10
rs4621481	T	G	T	G	0.00813086	-0.0105356	0.7149	0.705378	0.0157078	0.39				0.00115935	2.331E-12
rs4635115	T	C	T	C	-0.007762	-0.0298132	0.1742	0.177681	0.0185591	0.17	rs73015142	C	T	0.00138001	1.858E-08
rs4650228	A	G	A	G	-0.0084733	-0.0137933	0.4639	0.465022	0.0142505	0.19				0.00104925	6.694E-16
rs4652548	T	C	T	C	0.00804295	-0.0190727	0.5889	0.597492	0.014466	0.25				0.00106345	3.932E-14
rs4654441	A	G	A	G	0.00724157	-0.0126552	0.5755	0.578547	0.0143686	0.47				0.00105865	7.913E-12
rs4656013	T	C	T	C	0.00718768	-0.0033115	0.5799	0.584962	0.0143912	0.780001				0.00106919	1.789E-11
rs4662573	T	C	T	C	0.00692923	-0.0016187	0.5362	0.543253	0.014263	0.99				0.00104926	3.998E-11
rs4662909	A	G	A	G	-0.0062249	-0.0069307	0.6358	0.622219	0.0146449	0.48				0.00108738	1.036E-08
rs4663416	T	C	T	C	0.00619393	-0.006436	0.5336	0.523921	0.0142006	0.68				0.00104902	3.544E-09
rs4663617	A	T	A	T	0.0103504	-0.0350052	0.2399	0.235935	0.0169573	0.05				0.00122635	3.173E-17
rs4667489	T	C	T	C	0.0086329	-0.008996	0.5694	0.570646	0.0143923	0.57				0.00105674	3.103E-16
rs4668897	C	G	C	G	0.00787334	0.00150782	0.2477	0.253804	0.0164217	0.630001				0.00121549	9.316E-11
rs4677087	A	G	A	G	-0.0060622	-0.0050165	0.6377	0.63403	0.0147265	0.67				0.0010886	2.562E-08
rs4678463	T	G	T	G	-0.0095026	-0.001591	0.2518	0.259752	0.0162213	0.97				0.00120552	3.217E-15
rs4680176	T	C	T	C	0.00688731	-0.031891	0.3796	0.3713	0.0146919	0.0140001				0.00107823	1.685E-10
rs4685448	A	G	A	G	-0.0067876	0.00552503	0.4403	0.436477	0.0143868	0.59				0.00105405	1.198E-10
rs4697062	T	C	T	C	0.00775689	-0.0039306	0.4365	0.444918	0.0142983	0.7				0.00105505	1.951E-13
rs4709383	A	G	A	G	0.0125449	-0.0256761	0.9234	0.920339	0.0262566	0.41	rs73017548	C	G	0.00196873	1.863E-10
rs4709995	T	C	T	C	-0.0073586	0.0281281	0.3957	0.382338	0.0146082	0.13				0.00107005	6.106E-12
rs4714990	T	C	T	C	0.00653301	0.0252967	0.6197	0.628326	0.0146829	0.0439997				0.00107785	1.35E-09
rs4724085	A	C	A	C	-0.0087458	-0.0576974	0.6856	0.691191	0.0154417	0.00027				0.00112782	8.844E-15
rs4725065	A	G	A	G	-0.0121832	0.00105728	0.4896	0.486289	0.0142214	0.97				0.00104673	2.613E-31
rs4726070	A	G	A	G	0.0102141	-0.0382083	0.5979	0.588685	0.0144998	0.0109999	rs1345278	T	C	0.00106716	1.054E-21
rs4728354	T	C	T	C	0.00986285	0.0250683	0.5508	0.550174	0.0142895	0.0569994				0.00105237	7.143E-21
rs4737137	T	C	T	C	0.00713958	-0.0222517	0.7432	0.74158	0.016209	0.21				0.00120553	3.173E-09
rs4739408	T	G	T	G	0.00617606	0.0239613	0.5363	0.545738	0.0142338	0.14				0.00104971	4.011E-09

rs474000	A	G	A	G	0.00642198	-0.0146868	0.441	0.446099	0.0142702	0.38				0.00105387	1.101E-09
rs4741600	T	C	T	C	0.0158013	-0.0081376	0.3404	0.335504	0.0150056	0.82				0.00110472	2.087E-46
rs474210	A	C	A	C	-0.0062701	0.0112609	0.4802	0.474036	0.0142952	0.450001				0.00104733	2.143E-09
rs4751360	T	C	T	C	0.00909742	-0.0145002	0.4725	0.467246	0.0142248	0.21				0.00104823	4.012E-18
rs4772268	A	G	A	G	0.0099177	-0.0246456	0.3342	0.327588	0.0152894	0.0920005				0.00110983	4.025E-19
rs477723	T	C	T	C	0.00775909	-0.0170019	0.3745	0.367006	0.014758	0.17				0.00108112	7.119E-13
rs4780865	A	G	A	G	-0.0142523	0.0306508	0.0897	0.088144	0.0250759	0.28				0.00183115	7.057E-15
rs4785187	A	G	A	G	-0.0116212	0.00643993	0.2233	0.223031	0.0170665	0.66				0.00125747	2.418E-20
rs4785819	T	C	T	C	-0.0143991	-0.0016304	0.8991	0.897267	0.0233703	0.92				0.00174108	1.334E-16
rs4788080	T	C	T	C	-0.0133895	-0.0138343	0.3595	0.368734	0.0146459	0.37				0.00109059	1.203E-34
rs4790841	T	C	T	C	0.00999353	-0.0296738	0.154	0.15448	0.01971	0.13				0.00145289	6.067E-12
rs4793084	T	G	T	G	-0.0097328	0.008176	0.6939	0.700491	0.0155266	0.58				0.00113668	1.101E-17
rs4793090	A	G	A	G	0.00712775	-0.0189814	0.6671	0.656332	0.0149567	0.14				0.00111035	1.366E-10
rs4805761	A	G	A	G	-0.0139463	-0.0076577	0.1567	0.156867	0.0195268	0.62				0.00144818	5.971E-22
rs4818226	A	G	A	G	0.0112177	-0.0202566	0.3202	0.316244	0.0152831	0.15				0.00112168	1.507E-23
rs4821904	T	C	T	C	0.00635779	0.0050741	0.6061	0.62213	0.0147134	0.43				0.00107184	3.002E-09
rs483673	A	G	A	G	-0.0071194	-0.0086175	0.2455	0.252229	0.0163677	0.67				0.00121613	4.8E-09
rs4846724	A	G	A	G	0.00766902	-0.0054495	0.5326	0.533171	0.014279	0.81				0.00104874	2.623E-13
rs4848732	A	G	A	G	-0.0071558	0.0133844	0.446	0.460663	0.0142557	0.28				0.00105266	1.06E-11
rs4850810	T	G	T	G	-0.0131458	0.0175137	0.5292	0.537539	0.0143304	0.2				0.00105089	6.678E-36
rs4855037	T	G	T	G	-0.0061039	0.00883813	0.425	0.43207	0.0143147	0.37				0.00105994	8.475E-09
rs4859144	T	C	T	C	0.00878807	-0.0119663	0.33	0.322742	0.0151944	0.37				0.0011128	2.843E-15
rs4859423	T	C	T	C	0.009336	-0.0059498	0.8771	0.890003	0.022707	0.95				0.00159372	4.68E-09
rs4864881	T	C	T	C	-0.0069829	0.0173359	0.6067	0.598074	0.0144631	0.29				0.0010734	7.738E-11
rs4867608	A	T	A	T	0.00775729	0.0104031	0.695	0.686949	0.0153239	0.49				0.00113726	9.023E-12
rs4869579	T	C	T	C	0.00633947	-0.008035	0.5202	0.515601	0.0143245	0.51				0.0010478	1.442E-09
rs4872449	A	G	A	G	0.00953027	0.00590692	0.4304	0.431073	0.0143413	0.79				0.00105693	1.94E-19
rs4876775	A	G	A	G	-0.0112554	-0.0100834	0.3152	0.318031	0.0152266	0.46				0.0011264	1.648E-23
rs4879832	T	C	T	C	-0.0063744	0.00283375	0.3772	0.384686	0.0145774	0.67				0.00107971	3.556E-09
rs4883624	T	C	T	C	-0.0068898	-0.0163588	0.4473	0.447732	0.0143324	0.19				0.00105281	5.983E-11
rs489408	A	G	A	G	0.00766171	-0.0010032	0.4529	0.468232	0.0142099	0.77				0.00105187	3.249E-13
rs4894651	T	C	T	C	0.00914679	-0.0280431	0.4397	0.427879	0.0143445	0.0359998				0.0010542	4.094E-18
rs4894674	T	C	T	C	0.00611983	0.00430236	0.5381	0.53429	0.0142712	0.709999				0.00104956	5.516E-09
rs4899095	T	C	T	C	-0.0096425	-0.0299274	0.8937	0.896108	0.0233347	0.2				0.00170799	1.647E-08
rs4904523	A	G	A	G	-0.0074601	0.00979997	0.5176	0.512865	0.0142066	0.57				0.00104716	1.046E-12
rs4904871	A	G	A	G	-0.0059091	-0.0091567	0.4502	0.454663	0.0143129	0.35				0.00105203	1.941E-08
rs490535	T	C	T	C	0.00694748	-0.0144189	0.3345	0.343632	0.0149786	0.33				0.00110933	3.777E-10
rs4909854	T	G	T	G	-0.0063633	0.0006923	0.6173	0.616529	0.0146193	0.95				0.001077	3.456E-09
rs4911257	T	C	T	C	0.00979146	0.00787815	0.6131	0.606691	0.0145495	0.68				0.00107435	7.958E-20
rs4923705	A	G	A	G	0.00670683	0.0109991	0.7592	0.761578	0.0166523	0.59				0.00122379	4.243E-08
rs4925093	T	G	T	G	-0.0073556	0.0192546	0.482	0.466402	0.014216	0.15				0.00104719	2.153E-12
rs4934173	A	G	A	G	-0.0073589	0.0218596	0.1962	0.192596	0.0180451	0.34				0.00131816	2.37E-08
rs4934583	A	G	A	G	-0.007095	0.0171457	0.6421	0.63688	0.0149529	0.27				0.00109166	8.069E-11
rs4940668	A	G	A	G	-0.0068672	0.00774279	0.7003	0.699766	0.0154983	0.55				0.00114248	1.844E-09
rs4943074	A	C	A	C	0.00810865	0.0130274	0.5489	0.555188	0.014366	0.34				0.00105155	1.251E-14
rs4961398	A	T	A	T	0.00748284	-0.0152389	0.2981	0.298849	0.0155415	0.47				0.00114778	7.042E-11
rs4964731	A	C	A	C	-0.0070806	0.00025076	0.4815	0.48752	0.0141844	0.9				0.00104722	1.369E-11
rs4975219	T	C	T	C	0.0136769	0.0210312	0.9502	0.953302	0.0336625	0.5				0.00240542	1.3E-08
rs4981711	T	C	T	C	0.00841414	-0.0267659	0.6167	0.610355	0.0145632	0.0850002				0.00108278	7.795E-15
rs5021426	T	C	T	C	-0.0111567	0.0162427	0.7246	0.729074	0.0159547	0.18	rs4635727	G	A	0.00117133	1.657E-21
rs502245	A	G	A	G	0.00810193	-0.011346	0.1489	0.150423	0.0198296	0.51				0.00147044	3.594E-08
rs502506	A	G	A	G	-0.0066883	-0.0164572	0.4944	0.483461	0.0142115	0.17				0.00104657	1.653E-10
rs515513	A	C	A	C	-0.0087432	0.00021657	0.5332	0.528111	0.0143626	0.97				0.00105521	1.171E-16
rs517052	A	T	A	T	-0.0063534	-0.0197505	0.5954	0.584793	0.0144144	0.2				0.0010683	2.733E-09
rs526156	A	C	A	C	-0.0098475	-0.0366738	0.1108	0.116693	0.022161	0.0909997				0.00166713	3.492E-09
rs529020	T	C	T	C	-0.0063583	0.0100325	0.3172	0.3129	0.0153842	0.57				0.00112841	1.754E-08

rs537342	T	C	T	C	-0.0078968	-0.0026139	0.4215	0.425683	0.0143961	0.81				0.0010661	1.292E-13
rs538249	A	C	A	C	-0.0067376	0.00612623	0.5738	0.576181	0.0143728	0.48				0.00106059	2.121E-10
rs5396	T	C	T	C	0.00726311	-0.021419	0.7158	0.721747	0.0158417	0.21				0.00116045	3.874E-10
rs541969	T	C	T	C	-0.0058991	-0.0019512	0.5114	0.506454	0.0142157	0.81				0.00104678	1.749E-08
rs55633081	A	T	A	T	0.0115742	-0.0206844	0.744	0.737453	0.0161912	0.17				0.00119896	4.748E-22
rs55709029	T	C	T	C	-0.0066542	0.00464059	0.2488	0.253359	0.0164307	0.77				0.00121435	4.27E-08
rs55749333	T	C	T	C	-0.006646	0.0185308	0.6416	0.638538	0.0147789	0.23				0.00109118	1.123E-09
rs55787231	T	C	T	C	0.00731992	0.00102268	0.2098	0.197738	0.0178453	0.86				0.00128511	1.226E-08
rs55897719	A	C	A	C	-0.0126672	0.00369704	0.3055	0.316786	0.015282	0.54				0.00114572	2.042E-28
rs55901919	T	C	T	C	-0.00895	-0.0073062	0.2718	0.268299	0.0160639	0.44				0.00117615	2.75E-14
rs559073	A	G	A	G	0.00576378	-0.0267722	0.5304	0.540731	0.014254	0.13				0.00104845	3.846E-08
rs55972083	T	G	T	G	-0.0086653	-0.0359404	0.2153	0.217322	0.0172879	0.0350002	rs897627	T	C	0.00128189	1.379E-11
rs55972942	T	C	T	C	-0.0065246	0.0113001	0.6574	0.66079	0.015012	0.52				0.00110271	3.28E-09
rs55975662	A	G	A	G	0.0153152	-0.0153013	0.8566	0.860781	0.0204837	0.52				0.00149296	1.086E-24
rs56016333	T	C	T	C	-0.0138325	-0.017245	0.6488	0.657416	0.0149758	0.43				0.00110284	4.38E-36
rs56059718	A	C	A	C	-0.0121649	0.0168411	0.1943	0.19118	0.0180664	0.4				0.00132318	3.805E-20
rs560701	A	G	A	G	0.0091612	-0.0052444	0.7005	0.708701	0.015602	0.709999				0.00114933	1.579E-15
rs56101188	T	C	T	C	0.0102762	-0.0018295	0.9054	0.907574	0.0247467	0.95				0.00186637	3.663E-08
rs56103251	T	C	T	C	0.0126202	-0.0234236	0.0623	0.063038	0.0291827	0.58				0.00217044	6.076E-09
rs56133711	A	G	A	G	-0.0122384	0.0077041	0.2461	0.259345	0.0161925	0.6				0.00121479	7.162E-24
rs561655	A	G	A	G	-0.0089131	0.00704146	0.6553	0.652229	0.0149	0.54				0.00110096	5.691E-16
rs56181223	A	T	A	T	-0.0083845	0.00421475	0.343	0.349694	0.0149007	0.53				0.0011024	2.839E-14
rs56194430	T	C	T	C	-0.0186138	0.00444158	0.1669	0.169316	0.019086	0.95				0.00140674	5.716E-40
rs56317140	A	C	A	C	-0.0100802	-0.0303317	0.8733	0.871505	0.0217671	0.0990011				0.0015961	2.688E-10
rs56322375	T	G	T	G	-0.0075629	0.00608853	0.2327	0.240062	0.0169802	0.74				0.00124553	1.26E-09
rs56354797	A	G	A	G	-0.0061488	0.00084871	0.4491	0.50118	0.014235	0.9	rs1195233	C	T	0.00106059	6.722E-09
rs56355837	A	C	A	C	-0.0084241	-0.0149866	0.6641	0.671654	0.0151763	0.31				0.00110829	2.942E-14
rs56374036	A	G	A	G	-0.0087702	0.0248051	0.7872	0.787328	0.017458	0.18				0.00128475	8.695E-12
rs56409354	A	G	A	G	-0.0159882	-0.0110713	0.2182	0.22271	0.0170815	0.5				0.00126688	1.639E-36
rs564887699	C	G	C	G	0.0400029	-0.0060144	0.9844	0.984441	0.0576154	0.87				0.00446756	3.427E-19
rs565960	T	C	T	C	0.0087496	0.00525011	0.7114	0.726744	0.0160191	0.649999				0.00115594	3.752E-14
rs567775	T	C	T	C	0.00697067	0.0182044	0.4947	0.504691	0.014198	0.13				0.00104657	2.727E-11
rs56852527	T	C	T	C	0.00915296	0.00069748	0.2054	0.211662	0.0173554	0.93				0.0012952	1.588E-12
rs57123286	A	G	A	G	0.00790148	0.0281597	0.8396	0.838813	0.0192909	0.0870001				0.00142604	3.01E-08
rs57214198	T	G	T	G	-0.015497	0.0568468	0.0517	0.051214	0.0323402	0.0729995	rs1421120	A	T	0.00236329	5.473E-11
rs57319644	A	G	A	G	0.00944124	-0.0050942	0.4181	0.419237	0.014398	0.56				0.00106084	5.585E-19
rs57349798	A	G	A	G	0.00949562	0.00923214	0.4117	0.405653	0.0145727	0.57				0.00106352	4.319E-19
rs574967	A	G	A	G	0.00757265	-0.0064926	0.3273	0.339783	0.0150763	0.75	rs10774515	A	G	0.00111514	1.118E-11
rs5759002	A	G	A	G	0.00831722	-0.0085262	0.4878	0.480848	0.0142245	0.5				0.00104711	1.977E-15
rs5765717	T	G	T	G	-0.0072398	0.00201209	0.4867	0.489371	0.0142266	0.91				0.00104688	4.659E-12
rs5771204	T	C	T	C	0.00674104	-0.0073246	0.5802	0.579081	0.0143734	0.48				0.00106529	2.481E-10
rs57810758	A	G	A	G	0.0102677	-0.023108	0.8967	0.897457	0.0234182	0.34				0.00171976	2.361E-09
rs578527	A	G	A	G	0.00736617	-0.0201316	0.6742	0.6705	0.015139	0.23				0.0011166	4.189E-11
rs57985238	C	G	C	G	-0.0090887	0.0136732	0.603	0.601177	0.014529	0.3				0.00106998	1.99E-17
rs58130172	T	C	T	C	0.00975869	0.0300463	0.278	0.274364	0.0161466	0.032				0.00118065	1.389E-16
rs58137875	A	G	A	G	-0.0104735	0.0017136	0.2691	0.262346	0.0161423	0.85				0.00118032	7.1E-19
rs58385891	A	G	A	G	-0.0087955	0.00838622	0.1806	0.178808	0.0189507	0.760001				0.00136799	1.28E-10
rs58622306	A	C	A	C	0.0161534	0.0199279	0.9668	0.9666	0.0395182	0.56				0.00294958	4.344E-08
rs586829	A	G	A	G	0.00991755	0.0110119	0.6292	0.634796	0.0148487	0.47				0.0010834	5.487E-20
rs586875	A	G	A	G	0.00804842	0.0216517	0.4245	0.426871	0.0143266	0.08				0.00105896	2.96E-14
rs58694847	C	G	C	G	-0.0117414	0.0177394	0.2595	0.262274	0.0162033	0.33				0.00119366	7.843E-23
rs587021	T	G	T	G	0.00724957	-0.0015066	0.5805	0.583124	0.0144118	0.82	rs1184626	A	C	0.00106162	8.571E-12
rs590013	T	C	T	C	0.011071	0.00365648	0.6719	0.678665	0.0151872	0.67				0.00111444	2.962E-23
rs59093198	T	C	T	C	-0.0071103	0.0136547	0.6618	0.675618	0.0151741	0.23				0.00110616	1.293E-10
rs59123361	A	G	A	G	-0.0157301	-0.019125	0.1027	0.103293	0.0232931	0.53				0.00173675	1.34E-19
rs59234174	T	C	T	C	-0.0103012	0.036839	0.1566	0.155653	0.0196126	0.0710003				0.00144296	9.419E-13

rs596160	A	G	A	G	-0.0101737	0.0186864	0.4223	0.424687	0.0144433	0.16				0.00105968	7.94E-22
rs59832377	T	C	T	C	-0.0062434	0.0102918	0.5576	0.55871	0.0142717	0.46				0.00105352	3.095E-09
rs59903549	T	C	T	C	-0.0150095	0.0630933	0.0752	0.068182	0.0282443	0.0409996				0.00198417	3.895E-14
rs60041956	T	C	T	C	-0.006241	0.00295995	0.6623	0.669874	0.0152008	0.91				0.0011125	2.023E-08
rs60053512	A	G	A	G	0.0123262	-6.33E-05	0.1559	0.155305	0.0196069	0.9				0.00144242	1.277E-17
rs6008628	A	C	A	C	-0.0071137	-0.0084109	0.7795	0.776033	0.0170339	0.55				0.00126894	2.068E-08
rs6020560	T	C	T	C	-0.0095654	-0.0091093	0.5245	0.530489	0.0142381	0.649999				0.00104786	6.936E-20
rs6024420	T	C	T	C	-0.0069638	-0.0160405	0.3061	0.302889	0.0155411	0.29				0.0011357	8.707E-10
rs6028084	T	C	T	C	0.0151426	-0.0029626	0.6106	0.614514	0.0146931	0.82				0.00107339	3.438E-45
rs60291849	A	T	A	T	-0.0061561	0.0155636	0.617	0.609396	0.0145798	0.44				0.00107639	1.07E-08
rs60307735	T	C	T	C	0.00922929	-0.0166805	0.3395	0.347539	0.0149185	0.31				0.0011053	6.832E-17
rs6030819	A	T	A	T	-0.011383	-0.0050952	0.1958	0.194935	0.0180682	0.92				0.001319	6.12E-18
rs6035413	A	T	A	T	-0.0088227	0.0241967	0.6516	0.651743	0.0149255	0.0439997				0.0010982	9.482E-16
rs6035877	A	C	A	C	0.00721031	-0.0016926	0.5342	0.530556	0.0142248	0.98				0.00104896	6.261E-12
rs6044142	A	G	A	G	-0.0073916	-0.0049629	0.5694	0.560431	0.0143069	0.58				0.00105674	2.651E-12
rs60613433	A	G	A	G	0.0148774	0.0133589	0.0962	0.093596	0.0243745	0.56				0.00177478	5.184E-17
rs6061441	T	C	T	C	-0.0082819	0.0250802	0.2015	0.211559	0.0174134	0.17				0.00130448	2.173E-10
rs60637254	C	G	C	G	-0.0083855	0.0308736	0.3972	0.40475	0.014483	0.0280001				0.00106949	4.483E-15
rs6063897	C	G	C	G	-0.0097019	0.00094641	0.2431	0.246123	0.0166883	0.92				0.00122251	2.087E-15
rs6080772	A	T	A	T	0.00754027	-0.002487	0.1754	0.169499	0.018922	0.760001				0.00137586	4.246E-08
rs60814418	T	C	T	C	-0.0226273	0.0184429	0.2242	0.226442	0.0169448	0.450001				0.0012609	5.231E-72
rs60841615	T	G	T	G	0.0101053	0.00332895	0.0959	0.098512	0.023875	0.77				0.00177703	1.294E-08
rs6088618	A	G	A	G	0.00813085	0.00943672	0.449	0.38724	0.0145761	0.630001	rs6088620	G	A	0.00105199	1.084E-14
rs6091534	T	C	T	C	0.00737631	-0.007776	0.3794	0.390532	0.0145521	0.88				0.00107834	7.9E-12
rs6093705	A	C	A	C	0.00953909	-0.0041931	0.6805	0.665975	0.0150901	0.709999				0.00112218	1.885E-17
rs61160187	A	G	A	G	-0.019626	0.0108488	0.6118	0.607575	0.0145678	0.35				0.00107393	1.319E-74
rs61194013	T	C	T	C	0.011408	-0.0415527	0.0731	0.070515	0.0279434	0.12				0.00202238	1.692E-08
rs6122735	T	C	T	C	0.00734591	0.00064981	0.3927	0.400461	0.0144979	0.75				0.00107147	7.096E-12
rs61265411	C	G	C	G	0.0167102	-0.006129	0.9656	0.965898	0.0393081	0.91	rs9789733	G	A	0.00288888	7.284E-09
rs61274751	T	C	T	C	0.00936644	0.0372163	0.8821	0.882727	0.0220858	0.051				0.00162276	7.852E-09
rs61546403	T	C	T	C	0.00701186	-0.0108433	0.7137	0.708878	0.0156694	0.41				0.00115804	1.405E-09
rs61746505	C	G	C	G	0.0152789	-0.0363764	0.0483	0.04943	0.0327633	0.17				0.00245669	4.999E-10
rs61747226	T	C	T	C	-0.0188969	-0.0073113	0.0434	0.047723	0.0333036	0.88				0.00259682	3.412E-13
rs61757207	A	G	A	G	0.0352555	-0.0879305	0.9859	0.985193	0.0586343	0.0779992				0.0052699	2.233E-11
rs61768457	T	C	T	C	-0.018972	-0.0073563	0.0251	0.026684	0.04492	0.630001				0.00334857	1.462E-08
rs61822065	A	G	A	G	-0.0066243	0.0368387	0.5453	0.531893	0.0142707	0.015				0.00105083	2.907E-10
rs61902856	T	C	T	C	-0.0093127	0.0227143	0.2799	0.288865	0.0157322	0.0929994				0.00116551	1.345E-15
rs61937385	T	C	T	C	0.00965215	-0.0004261	0.8919	0.886826	0.0224051	0.99				0.00168516	1.018E-08
rs61940318	A	G	A	G	-0.008345	-0.0037051	0.1974	0.197783	0.017889	0.9				0.00131459	2.179E-10
rs61991641	T	C	T	C	-0.0092851	0.00611001	0.3456	0.348512	0.0149754	0.62				0.00110347	3.962E-17
rs62070652	T	C	T	C	-0.0070866	-0.0026508	0.2705	0.269363	0.0160259	0.98				0.00117792	1.782E-09
rs62078384	T	C	T	C	-0.0059224	0.0239966	0.5212	0.52194	0.014214	0.19				0.00104962	1.676E-08
rs62079997	T	C	T	C	-0.0067735	0.00165916	0.5644	0.57915	0.0143595	0.6				0.00105746	1.497E-10
rs62083308	T	C	T	C	-0.0078175	-0.0015086	0.1745	0.176168	0.018648	0.86				0.00137866	1.422E-08
rs62086577	A	G	A	G	0.00739293	-0.0043503	0.2153	0.220397	0.0171213	0.87				0.00128205	8.098E-09
rs62121816	T	C	T	C	0.00606649	0.00423971	0.4615	0.481154	0.0141916	0.62				0.00104962	7.494E-09
rs62137021	T	G	T	G	0.0100371	0.00502381	0.3163	0.31792	0.0152612	0.74				0.00112554	4.763E-19
rs62172117	A	G	A	G	0.0135597	-0.0133535	0.3525	0.357402	0.0148301	0.33				0.00109525	3.349E-35
rs62179650	A	G	A	G	0.00870135	-0.0030006	0.2979	0.304539	0.0154207	0.94				0.0011511	4.06E-14
rs62181533	A	C	A	C	0.00668342	-0.0148755	0.5083	0.509334	0.0143151	0.18				0.00104912	1.888E-10
rs62183418	A	G	A	G	0.0119589	0.00297557	0.8881	0.886996	0.0224293	0.98				0.00165984	5.8E-13
rs62194510	A	G	A	G	0.00627424	-0.0273024	0.3842	0.382306	0.0146068	0.04				0.0010759	5.499E-09
rs621996	A	G	A	G	0.00623125	-0.0260841	0.6251	0.624169	0.0147797	0.0819993				0.00108089	8.165E-09
rs62232802	A	C	A	C	0.00990567	0.0220399	0.174	0.175215	0.0187882	0.17				0.00138066	7.251E-13
rs62234664	A	G	A	G	-0.0066062	0.0153991	0.2814	0.287125	0.0157061	0.32				0.00116361	1.37E-08
rs62244884	A	G	A	G	0.0165461	-0.0020094	0.4275	0.431128	0.0143793	0.8				0.00105808	4.032E-55

rs62251261	A	T	A	T	-0.0070277	-0.004662	0.7486	0.740548	0.0165979	0.74				0.0012122	6.719E-09
rs62256287	A	G	A	G	0.0102039	0.0138943	0.331	0.343399	0.0149709	0.27				0.00111195	4.449E-20
rs62257211	T	C	T	C	-0.0101868	0.016555	0.714	0.716997	0.0157832	0.3				0.00115792	1.399E-18
rs62262312	C	G	C	G	0.0188859	0.0119391	0.162	0.161464	0.0193344	0.450001				0.00142015	2.359E-40
rs62267594	C	G	C	G	0.0123667	0.00466946	0.0878	0.089805	0.0247703	0.85	rs62267597	G	A	0.00185004	2.312E-11
rs62286094	T	C	T	C	0.00816774	0.0131738	0.1586	0.161548	0.0194592	0.36				0.00143279	1.192E-08
rs62300090	T	C	T	C	0.00942085	0.0208858	0.1032	0.101478	0.0235194	0.64				0.00171998	4.324E-08
rs623200	T	C	T	C	0.00704257	0.00374714	0.3849	0.381952	0.0146082	0.84				0.00107539	5.79E-11
rs62327424	A	G	A	G	0.0101173	0.0435325	0.0991	0.095874	0.0241824	0.0430002				0.00175121	7.597E-09
rs62392801	T	C	T	C	-0.0061578	-0.0233358	0.6494	0.64676	0.0148686	0.0909997				0.00109706	1.992E-08
rs62409395	T	C	T	C	0.0105597	-0.0144507	0.7618	0.75658	0.0176439	0.39				0.00126557	7.213E-17
rs62439683	A	C	A	C	-0.0120726	0.0106554	0.2506	0.252485	0.0163663	0.58				0.00120744	1.55E-23
rs62442809	T	C	T	C	-0.0090646	0.0154683	0.284	0.283906	0.015838	0.44				0.0011607	5.746E-15
rs62515110	T	C	T	C	0.00925026	0.0161406	0.811	0.812934	0.0183963	0.27				0.00134286	5.642E-12
rs62532760	C	G	C	G	-0.0075687	0.0115979	0.663	0.666932	0.0153574	0.34				0.00110973	9.07E-12
rs62541086	T	G	T	G	0.00689511	-0.0333868	0.6488	0.64553	0.0149095	0.0280001				0.00109663	3.219E-10
rs62543169	T	C	T	C	-0.0116492	-0.0105904	0.4276	0.434695	0.0142969	0.719999				0.00105779	3.33E-28
rs62583551	A	G	A	G	0.00832249	0.0113017	0.2276	0.227065	0.01703	0.48				0.00126187	4.231E-11
rs62622853	T	C	T	C	0.0196192	0.0362305	0.9737	0.97199	0.0429935	0.35				0.00332362	3.57E-09
rs628720	T	C	T	C	-0.009148	0.0270372	0.634	0.634327	0.0148557	0.0749998				0.00108699	3.899E-17
rs631287	A	G	A	G	0.008508	-0.0263967	0.5583	0.557376	0.0142752	0.0560003				0.00105426	7.036E-16
rs63303162	A	G	A	G	0.0112215	0.00123895	0.5593	0.555661	0.0143027	0.96	rs2581761	A	G	0.00105437	1.888E-26
rs633051	A	G	A	G	-0.0067657	-0.0290841	0.2453	0.263217	0.0160881	0.089	rs607431	C	T	0.00121612	2.648E-08
rs637096	A	G	A	G	0.00639241	0.0139742	0.4187	0.451856	0.0143345	0.33	rs559417	T	C	0.00106708	2.091E-09
rs6425839	T	G	T	G	0.00790107	-0.0079669	0.5082	0.511623	0.0143278	0.62				0.00104953	5.135E-14
rs6428152	A	G	A	G	-0.0080543	0.0083742	0.2775	0.275285	0.0158772	0.56				0.00116859	5.486E-12
rs6439359	T	C	T	C	-0.0060669	-0.0017746	0.4698	0.462654	0.0142535	0.92				0.00104842	7.163E-09
rs6440854	A	G	A	G	0.00948754	-0.0150651	0.8769	0.878978	0.0219471	0.49				0.00159261	2.567E-09
rs6445633	A	C	A	C	0.00964476	0.0244579	0.3113	0.321252	0.0153542	0.0479999				0.00113008	1.402E-17
rs6451704	T	C	T	C	-0.0072587	0.00621079	0.5271	0.529199	0.0142101	0.5				0.00104926	4.574E-12
rs6457796	T	C	T	C	0.0104006	-0.0489213	0.7189	0.730774	0.0159843	0.00519996				0.0011664	4.811E-19
rs6465603	A	G	A	G	0.0110417	-0.0288278	0.1809	0.185999	0.0183829	0.18				0.00135933	4.546E-16
rs6466499	A	G	A	G	-0.0112536	0.0132194	0.2006	0.200093	0.0177719	0.450001				0.00130789	7.657E-18
rs6466819	A	G	A	G	-0.0103106	0.00486155	0.3678	0.360188	0.0148069	0.81				0.00108737	2.494E-21
rs6472213	T	C	T	C	-0.0074381	-0.0006157	0.3304	0.332977	0.0150367	0.91				0.00111261	2.301E-11
rs6472530	A	T	A	T	-0.0072962	0.0204162	0.6508	0.652613	0.0149525	0.15				0.00109838	3.085E-11
rs648044	A	G	A	G	0.00606119	0.0114564	0.3825	0.392505	0.0149085	0.35				0.00108045	2.021E-08
rs6495127	T	C	T	C	0.00787334	-1.427E-05	0.6959	0.711024	0.01564	0.73				0.00113759	4.475E-12
rs6500234	T	C	T	C	0.00689443	-0.0051464	0.6437	0.652324	0.0149393	0.8				0.00109275	2.808E-10
rs6504417	T	C	T	C	-0.0063295	-0.0162696	0.3446	0.344872	0.0149459	0.28				0.00110104	9.011E-09
rs6504875	T	G	T	G	0.00898379	-0.0147862	0.5581	0.567971	0.0144039	0.25				0.00105407	1.558E-17
rs6534338	T	C	T	C	0.0104741	-0.0076925	0.3014	0.296422	0.0156275	0.36				0.00114042	4.141E-20
rs6534591	A	G	A	G	-0.00609	-0.0045163	0.3614	0.366453	0.0147336	0.84				0.00108919	2.259E-08
rs6534704	A	T	A	T	-0.0149996	0.00297878	0.0842	0.092274	0.0245862	0.81				0.00188823	1.964E-15
rs6539284	T	C	T	C	-0.0072088	0.00726711	0.5931	0.571378	0.0143457	0.67				0.00106643	1.382E-11
rs6545559	T	C	T	C	-0.0076694	-0.0143455	0.3196	0.318746	0.0152663	0.450001				0.00112209	8.215E-12
rs6547148	T	C	T	C	0.00627547	0.00082642	0.459	0.46367	0.0143537	0.98				0.00105014	2.293E-09
rs6547396	T	C	T	C	0.00772378	0.00852723	0.5972	0.595164	0.0144522	0.62				0.00106686	4.491E-13
rs654880	A	G	A	G	-0.0084191	-0.0011565	0.2224	0.224158	0.0170788	0.92				0.00126007	2.361E-11
rs6563363	T	G	T	G	-0.0092103	0.00864208	0.3732	0.364619	0.0147632	0.67				0.00108202	1.703E-17
rs6570660	T	C	T	C	-0.0117374	0.00297801	0.2688	0.243915	0.0165272	0.93	rs4526219	A	G	0.0011806	2.725E-23
rs6575340	A	G	A	G	-0.0090146	0.0253888	0.64	0.636034	0.0147804	0.12				0.00109021	1.355E-16
rs6587843	T	C	T	C	0.00741178	0.0115564	0.4833	0.490972	0.0142014	0.41				0.00104709	1.46E-12
rs658938	A	G	A	G	-0.0123583	0.0343305	0.194	0.199319	0.0177554	0.0369999				0.00132379	1.003E-20
rs660010	C	G	C	G	0.0186287	-0.0201601	0.1408	0.144065	0.0202363	0.32				0.0015044	3.244E-35
rs660549	T	C	T	C	-0.0103944	-0.0078952	0.5742	0.568408	0.0143171	0.57				0.00105822	8.985E-23

rs663234	C	G	C	G	0.00992597	-0.0161339	0.3996	0.392078	0.014601	0.21				0.00106857	1.553E-20
rs6669004	A	G	A	G	-0.007226	-0.0093947	0.7254	0.725457	0.0158864	0.630001				0.00117239	7.115E-10
rs66716825	C	G	C	G	-0.0112605	0.0189571	0.2426	0.232418	0.0168392	0.31				0.00122069	2.85E-20
rs66743759	T	G	T	G	0.00779977	-0.0121718	0.8286	0.822865	0.018611	0.32				0.00139056	2.031E-08
rs6684189	T	C	T	C	-0.0084437	-0.0106088	0.6302	0.622997	0.014724	0.62				0.00108421	6.792E-15
rs6689263	A	G	A	G	-0.0106131	-0.0154048	0.8872	0.884813	0.022282	0.52				0.00165429	1.402E-10
rs6693597	T	G	T	G	0.00896291	0.00651941	0.8645	0.865655	0.0208449	0.61				0.00153776	5.6E-09
rs66968950	A	C	A	C	0.00856892	0.00879627	0.6366	0.629286	0.0146816	0.54				0.0010889	3.562E-15
rs6697033	A	G	A	G	0.00692193	0.00221326	0.5459	0.546804	0.014269	0.9				0.00105124	4.566E-11
rs669952	A	G	A	G	0.00779624	-0.0102558	0.1875	0.183045	0.0184569	0.51				0.00134224	6.309E-09
rs67040074	T	C	T	C	-0.0121597	0.01242	0.3451	0.340139	0.0150166	0.53				0.00110097	2.33E-28
rs6704241	A	G	A	G	0.00859319	0.0125095	0.663	0.648259	0.0148607	0.38				0.00110715	8.38E-15
rs6704768	A	G	A	G	-0.0153293	0.00846141	0.5664	0.569464	0.0143358	0.46				0.00105609	9.672E-48
rs6706197	T	C	T	C	0.00648586	-0.0029541	0.4748	0.468958	0.0144586	0.74				0.00105138	6.864E-10
rs6711399	T	C	T	C	-0.0129307	0.0141669	0.181	0.170543	0.0188767	0.46	rs6711570	C	T	0.00136828	3.369E-21
rs6713695	A	G	A	G	-0.0105975	0.0109655	0.4684	0.461985	0.0142302	0.43				0.0010486	5.175E-24
rs6715321	T	C	T	C	-0.0097874	0.0118701	0.4284	0.429615	0.0143514	0.41				0.0010578	2.199E-20
rs6717169	T	C	T	C	0.0079815	0.0100793	0.8065	0.808526	0.018026	0.51				0.00132821	1.868E-09
rs6717900	A	G	A	G	0.00910517	0.0244995	0.3677	0.378515	0.0147073	0.051				0.00108549	4.929E-17
rs6721505	A	G	A	G	0.00736174	-0.0025561	0.3275	0.322193	0.0152539	0.97				0.00111528	4.092E-11
rs67224963	A	G	A	G	0.0102835	-0.0589634	0.2044	0.202501	0.0177067	0.00089				0.00129809	2.343E-15
rs6722777	T	C	T	C	-0.0147021	0.037314	0.0423	0.041496	0.0361458	0.41				0.00268078	4.143E-08
rs6728111	A	G	A	G	0.00608764	-0.0043912	0.4671	0.465195	0.0142772	0.84				0.00104878	6.466E-09
rs6731373	A	G	A	G	-0.0087233	-0.0195306	0.3456	0.358241	0.0147959	0.15				0.00110319	2.635E-15
rs6737433	C	G	C	G	0.0094532	-0.016727	0.7747	0.773372	0.0169608	0.3				0.00125313	4.564E-14
rs6743032	A	G	A	G	-0.0142717	0.0197391	0.0913	0.097164	0.0241791	0.34				0.00181663	3.962E-15
rs6747129	A	G	A	G	-0.0156817	-0.0388414	0.1026	0.101632	0.0235064	0.0810009				0.001728	1.137E-19
rs6748621	T	C	T	C	0.00636238	-0.0076178	0.6074	0.615616	0.0145863	0.98				0.00107374	3.107E-09
rs6756935	T	C	T	C	-0.0086516	-0.0087645	0.137	0.130346	0.0211729	0.38				0.00152222	1.318E-08
rs675806	A	C	A	C	-0.0080368	0.00903315	0.3454	0.343485	0.0149335	0.53				0.00110043	2.812E-13
rs67654519	T	C	T	C	-0.0086409	-0.0071667	0.4885	0.487594	0.0142025	0.7				0.00104678	1.519E-16
rs6779981	T	C	T	C	0.00675512	-0.0131012	0.5966	0.602348	0.014505	0.450001				0.0010666	2.394E-10
rs6782698	A	G	A	G	0.0138302	0.00414666	0.7537	0.748037	0.0163738	0.89				0.00121445	4.805E-30
rs67829508	A	G	A	G	-0.0091976	0.0170429	0.1855	0.181137	0.0184772	0.36				0.00135566	1.162E-11
rs67857806	A	G	A	G	0.00782405	-0.0370445	0.2258	0.227751	0.0169754	0.0359998				0.00125443	4.459E-10
rs6788064	A	G	A	G	-0.006979	0.0172042	0.4898	0.480999	0.0143269	0.31				0.00104745	2.692E-11
rs67981189	A	G	A	G	-0.0069947	0.0170233	0.6599	0.669655	0.0150891	0.15				0.00111123	3.084E-10
rs6802558	C	G	C	G	0.0063276	-0.0207231	0.2908	0.288459	0.0158802	0.1				0.00115524	4.319E-08
rs6808671	T	C	T	C	-0.0089387	0.0340069	0.368	0.379074	0.0146633	0.00700003				0.001085	1.742E-16
rs68140214	A	G	A	G	-0.0137907	0.0347066	0.1743	0.173967	0.0187781	0.0580003				0.00138271	1.994E-23
rs6815512	A	C	A	C	-0.0081479	0.00149355	0.5469	0.551817	0.0142684	0.58				0.00105114	9.08E-15
rs6824923	T	C	T	C	0.00910515	-0.048698	0.8341	0.835928	0.0192222	0.00659994				0.00140835	1.011E-10
rs6839051	A	C	A	C	-0.0069049	-0.0265562	0.3828	0.377118	0.0147031	0.0239999				0.0010768	1.435E-10
rs684192	A	G	A	G	-0.0068046	0.0132781	0.6353	0.641062	0.0148089	0.42				0.00108706	3.851E-10
rs6852084	A	C	A	C	-0.0104754	-0.0096633	0.6172	0.622281	0.01463	0.649999				0.0010765	2.228E-22
rs6860867	A	G	A	G	0.00852261	-0.0101516	0.1571	0.161633	0.0193957	0.73				0.0014383	3.113E-09
rs6864049	A	G	A	G	0.0083344	-0.0231207	0.4706	0.467551	0.0143862	0.0929994				0.00104846	1.873E-15
rs6866245	A	G	A	G	-0.0090057	0.00020807	0.6149	0.601918	0.0144948	0.760001				0.00107543	5.554E-17
rs6872619	A	G	A	G	-0.0085353	-0.0054233	0.1801	0.1767	0.0188445	0.54				0.00136186	3.663E-10
rs6880251	T	C	T	C	-0.0093501	-0.0267625	0.2305	0.226996	0.0169814	0.0870001				0.00124356	5.528E-14
rs6883584	T	G	T	G	0.0103717	0.018853	0.1283	0.116507	0.0220938	0.48				0.00156485	3.395E-11
rs6898748	T	C	T	C	-0.0066089	-0.0061314	0.5583	0.56307	0.014303	0.62				0.00105383	3.577E-10
rs6906818	T	C	T	C	0.00822943	0.00183183	0.5985	0.600827	0.0145039	0.79				0.00106743	1.261E-14
rs6907381	A	G	A	G	-0.0118461	0.00614646	0.1237	0.116095	0.0221743	0.92				0.00158928	9.094E-14
rs6911986	T	C	T	C	-0.0082766	-0.0065789	0.2682	0.262169	0.0162203	0.6				0.00118143	2.461E-12
rs6916517	T	C	T	C	0.00825743	0.0151483	0.2706	0.27548	0.0159164	0.33				0.00117778	2.365E-12

rs6930903	A	G	A	G	0.015874	0.0145823	0.7493	0.768337	0.0168087	0.22				0.00132543	4.726E-33
rs6946355	A	G	A	G	0.00691256	-0.0134078	0.462	0.453572	0.0142435	0.34				0.00104994	4.594E-11
rs6946362	T	C	T	C	0.0070532	-0.023857	0.6912	0.692972	0.0156537	0.17				0.0011345	5.062E-10
rs6950250	A	G	A	G	-0.0089317	0.00904882	0.1748	0.167551	0.0189933	0.649999				0.00137772	9.017E-11
rs6956241	T	G	T	G	0.0134098	-0.0023555	0.1403	0.14181	0.0204536	0.96				0.00150767	5.882E-19
rs6960056	A	G	A	G	0.0128551	-0.0115246	0.4397	0.430187	0.0144251	0.28				0.00105452	3.503E-34
rs6978797	T	C	T	C	0.00630731	0.0196104	0.6544	0.654565	0.0149312	0.18				0.00110059	9.984E-09
rs6986186	A	G	A	G	-0.0111785	0.0344347	0.9097	0.912893	0.0252211	0.15				0.00183047	1.014E-09
rs7002561	T	G	T	G	0.0105456	-0.05178	0.0877	0.089244	0.0249661	0.02				0.00185395	1.284E-08
rs7002655	A	G	A	G	0.00692229	-0.0213053	0.4425	0.44559	0.0142577	0.22				0.00105403	5.118E-11
rs7009856	C	G	C	G	0.00671103	-0.0110675	0.3887	0.384128	0.0146309	0.43				0.00107384	4.121E-10
rs7014300	A	C	A	C	0.00923732	0.0136465	0.1935	0.199214	0.0178693	0.23				0.00132473	3.105E-12
rs7026972	T	C	T	C	-0.0114958	-0.0162932	0.6439	0.637354	0.0147391	0.24				0.00109288	7.083E-26
rs7032484	A	G	A	G	0.0130126	0.00670819	0.566	0.55484	0.0142613	0.79				0.00105588	6.755E-35
rs7035315	A	G	A	G	-0.0086157	-0.0068423	0.6281	0.635658	0.0147644	0.719999				0.00108278	1.762E-15
rs7039819	A	G	A	G	0.00919465	-0.0223425	0.5813	0.572685	0.0143889	0.11				0.00106076	4.387E-18
rs7045411	A	G	A	G	0.0192529	-0.0089389	0.8494	0.853647	0.0200702	0.77				0.00146319	1.52E-39
rs705240	T	C	T	C	-0.0130133	-0.0112727	0.1853	0.184505	0.0182881	0.69				0.0013475	4.559E-22
rs705985	T	G	T	G	-0.0075323	-0.0074843	0.6046	0.598306	0.0144827	0.46				0.00107033	1.959E-12
rs707084	A	G	A	G	0.00658755	0.00984108	0.459	0.454656	0.0143036	0.35				0.00105004	3.528E-10
rs7072319	T	C	T	C	-0.0070683	0.00829491	0.494	0.496585	0.0142093	0.57				0.00104672	1.452E-11
rs7072915	T	G	T	G	0.00747702	-0.0259303	0.5414	0.556782	0.014312	0.0959997				0.00105136	1.142E-12
rs7074897	A	T	A	T	0.00797453	-0.0268311	0.8191	0.817921	0.0183995	0.2	rs1935996	A	T	0.00135951	4.469E-09
rs7078184	A	G	A	G	-0.0080996	0.00761656	0.1968	0.204185	0.0176442	0.53				0.00131627	7.594E-10
rs708912	T	C	T	C	0.0103668	0.00096128	0.7921	0.792222	0.0175957	0.98				0.00128942	8.995E-16
rs7095620	A	G	A	G	-0.0074602	0.00201243	0.3101	0.304805	0.0155749	0.86				0.00113153	4.315E-11
rs7097348	T	C	T	C	-0.0090539	0.0199705	0.7168	0.716711	0.015788	0.21				0.00116151	6.435E-15
rs7101996	T	C	T	C	-0.0066868	0.016212	0.5652	0.569782	0.0144576	0.28				0.00105582	2.4E-10
rs7109373	A	T	A	T	0.0104065	-0.0042502	0.804	0.806668	0.0181216	0.86				0.00131812	2.899E-15
rs7114098	A	C	A	C	-0.0084383	0.0218578	0.1691	0.16902	0.0189637	0.25				0.00139594	1.492E-09
rs7115551	A	C	A	C	0.0158522	-0.0014046	0.8916	0.887004	0.0225518	0.85				0.00168336	4.654E-21
rs7123652	T	C	T	C	-0.0073276	0.00161519	0.767	0.756935	0.0165999	0.95				0.00123776	3.216E-09
rs7125115	A	G	A	G	-0.0064547	0.00278864	0.3748	0.373009	0.0147173	0.77				0.00108407	2.618E-09
rs7125588	A	G	A	G	-0.0099608	0.0138705	0.5656	0.557502	0.0143582	0.19				0.00105635	4.122E-21
rs7126413	A	G	A	G	0.00877177	-0.0175828	0.6182	0.624276	0.0146524	0.2				0.00107803	4.064E-16
rs7131691	A	G	A	G	-0.0074794	0.0284566	0.4705	0.470699	0.0143521	0.0340001				0.00105066	1.088E-12
rs7134506	T	C	T	C	-0.0063282	-0.0127059	0.4363	0.441095	0.0143213	0.32				0.00105759	2.178E-09
rs71411521	T	C	T	C	0.0139823	0.00790817	0.1506	0.152523	0.0199223	0.709999				0.00146382	1.269E-21
rs7146843	A	G	A	G	0.00888843	-0.0023911	0.2408	0.287724	0.015808	0.780001	rs7156091	C	G	0.00123209	5.419E-13
rs7150195	T	G	T	G	0.0136491	0.010533	0.3758	0.381729	0.0146763	0.38				0.00108067	1.439E-36
rs71504489	T	C	T	C	0.0102114	0.01551	0.1207	0.131621	0.0209739	0.3				0.00160761	2.121E-10
rs7151326	T	G	T	G	-0.0086987	-0.0107681	0.3944	0.37474	0.0147273	0.450001				0.00107448	5.689E-16
rs71658797	A	T	A	T	-0.0174527	0.0282301	0.1116	0.120809	0.0218227	0.11				0.00166226	8.72E-26
rs717112	A	G	A	G	-0.0094248	-0.0079766	0.1804	0.18889	0.018128	0.67				0.00136099	4.362E-12
rs7172133	A	C	A	C	0.00741954	-0.0042044	0.6869	0.677343	0.0151771	0.58				0.00112881	4.923E-11
rs7173089	T	C	T	C	-0.009024	-0.0276065	0.3891	0.387779	0.0145464	0.0460002				0.00107403	4.393E-17
rs7176675	T	C	T	C	-0.0066485	-0.0310298	0.3673	0.364189	0.0147639	0.0309999				0.00108592	9.231E-10
rs717997	A	G	A	G	0.0129679	-0.0340292	0.4176	0.420303	0.0144221	0.0290001				0.00106141	2.492E-34
rs7184911	A	C	A	C	0.00758205	-0.0080854	0.5722	0.578378	0.0144852	0.49				0.00105759	7.562E-13
rs7195329	A	G	A	G	0.00856532	0.0143175	0.2635	0.265884	0.0160613	0.3				0.00118793	5.585E-13
rs7195739	A	G	A	G	0.00907162	-0.0064759	0.1481	0.151589	0.0198315	0.83				0.00147313	7.372E-10
rs7196426	A	C	A	C	0.00912097	-0.0014164	0.8361	0.839142	0.019324	0.98				0.00141368	1.106E-10
rs7204214	A	G	A	G	0.00832119	-0.0038949	0.788	0.781032	0.0171981	0.630001				0.00128817	1.05E-10
rs7212846	T	C	T	C	0.00783817	0.0127087	0.2471	0.249955	0.0166729	0.38				0.00121413	1.078E-10
rs7236339	A	G	A	G	-0.0153102	0.016694	0.2174	0.227706	0.0170201	0.14				0.00126874	1.569E-33
rs7240100	A	G	A	G	0.00727467	0.0196764	0.614	0.615467	0.0146445	0.28				0.00107482	1.304E-11

rs7240432	T	G	T	G	0.0103879	-0.0071702	0.3583	0.355295	0.0148308	0.69				0.00109125	1.738E-21
rs7247619	A	G	A	G	-0.01002	-0.036851	0.1007	0.103837	0.0232716	0.13				0.00173878	8.269E-09
rs7254263	T	C	T	C	-0.0099277	-0.0063934	0.2899	0.2884	0.0157136	0.649999				0.00115421	7.901E-18
rs72625841	T	G	T	G	0.00864832	0.0336976	0.7383	0.744866	0.0163201	0.015				0.00119074	3.779E-13
rs7263949	C	G	C	G	0.00981377	-0.018493	0.6923	0.70927	0.0156193	0.28	rs6073621	G	A	0.00113371	4.859E-18
rs72657787	A	G	A	G	-0.0149026	-0.0042018	0.0783	0.083758	0.025674	0.91				0.00194986	2.119E-14
rs72662300	T	G	T	G	0.0102219	0.00547138	0.3327	0.336118	0.0151344	0.61				0.00111128	3.629E-20
rs72672601	A	G	A	G	-0.0095302	-0.0076355	0.5715	0.576981	0.0143604	0.84				0.00105747	2.019E-19
rs72674843	T	C	T	C	-0.010692	0.0558437	0.7511	0.761179	0.0166322	0.00024				0.00121034	1.013E-18
rs72676302	T	C	T	C	0.0171384	0.02032	0.0452	0.0486	0.0332623	0.58				0.00253303	1.321E-11
rs72681238	T	C	T	C	-0.0120114	0.0682085	0.9329	0.928916	0.0281374	0.0129999				0.00209225	9.432E-09
rs72695265	T	C	T	C	-0.009616	-0.0119514	0.2292	0.234818	0.0167711	0.39				0.0012449	1.125E-14
rs72697581	A	G	A	G	0.0117513	0.0277622	0.9248	0.928724	0.0275566	0.22				0.00199152	3.619E-09
rs72717150	A	G	A	G	-0.0194229	-0.0168802	0.0747	0.072071	0.0274726	0.39				0.00199026	1.683E-22
rs72725852	T	C	T	C	0.00663025	0.00965347	0.3216	0.329667	0.0151248	0.52				0.00112056	3.277E-09
rs72748123	A	G	A	G	-0.0117951	-0.019674	0.1635	0.168058	0.0192145	0.24				0.00141507	7.742E-17
rs72751302	A	G	A	G	0.0110307	-0.023117	0.2027	0.202743	0.0176876	0.0940005				0.00131116	3.992E-17
rs72778512	C	G	C	G	-0.012231	-0.0230125	0.9123	0.911906	0.0250357	0.3				0.00185012	3.814E-11
rs72779695	T	C	T	C	-0.0139671	0.0058495	0.1181	0.118625	0.0221254	0.91				0.00162135	7.028E-18
rs72784111	T	C	T	C	-0.013502	0.0490984	0.913	0.91115	0.0249888	0.0389996				0.00185684	3.557E-13
rs7280000	C	G	C	G	0.00843317	-0.022476	0.4172	0.403226	0.0144086	0.051				0.0010613	1.92E-15
rs72801843	A	T	A	T	0.0151949	-0.006974	0.3036	0.301382	0.0154647	0.5				0.0011408	1.781E-40
rs72802661	A	C	A	C	-0.0259434	0.0227381	0.0231	0.026374	0.0451526	0.47				0.00350209	1.282E-13
rs72829857	A	G	A	G	-0.0170492	0.0144044	0.7527	0.765702	0.0167872	0.19				0.00121314	7.306E-45
rs72833096	C	G	C	G	-0.0106689	0.0535299	0.1481	0.157735	0.0195601	0.00309999				0.00147493	4.702E-13
rs72835052	A	G	A	G	0.0132743	-0.0371027	0.0664	0.064701	0.0295449	0.089				0.00211068	3.186E-10
rs72838750	T	G	T	G	-0.0087716	-0.0153333	0.4944	0.496989	0.0141879	0.18				0.00105352	8.348E-17
rs72842507	T	C	T	C	0.0125392	-0.0266706	0.1033	0.093485	0.0244349	0.22				0.00171947	3.052E-13
rs72850884	T	C	T	C	0.0106148	-0.0036109	0.9082	0.908142	0.0245743	0.84				0.00182337	5.836E-09
rs72887889	A	T	A	T	-0.0093675	0.00172554	0.8083	0.801543	0.0179441	0.87				0.00132965	1.856E-12
rs72890842	T	G	T	G	0.0125993	0.00556474	0.7563	0.755007	0.0164834	0.649999				0.00121881	4.761E-25
rs72899101	T	G	T	G	-0.0093654	0.0503649	0.8486	0.849767	0.0198885	0.00669993				0.00145981	1.403E-10
rs72915557	A	G	A	G	-0.0205705	0.0175668	0.0598	0.059802	0.0300801	0.46				0.00220694	1.158E-20
rs72938351	A	G	A	G	-0.0105145	0.0346859	0.8933	0.889883	0.0227491	0.0929994				0.00169952	6.146E-10
rs72944032	A	G	A	G	0.00855676	-0.0012552	0.2527	0.25822	0.016246	0.79				0.0012041	1.192E-12
rs7296742	A	G	A	G	-0.0085522	0.0133542	0.2849	0.290443	0.0159917	0.31				0.00116432	2.056E-13
rs729702	A	G	A	G	-0.0058878	0.00167422	0.4127	0.410537	0.0144417	0.74				0.00106307	3.044E-08
rs72976737	A	G	A	G	0.0251691	-0.0289076	0.0369	0.034003	0.03916	0.37				0.00277653	1.25E-19
rs7297828	A	G	A	G	0.00877341	0.0090765	0.7384	0.736326	0.016107	0.51				0.00119055	1.721E-13
rs72989297	T	G	T	G	-0.0100817	-0.0324922	0.1344	0.132138	0.0210458	0.15				0.0015455	6.873E-11
rs72993796	T	C	T	C	0.0148511	-0.0142134	0.882	0.881556	0.0221453	0.52				0.00163104	8.583E-20
rs73009622	C	G	C	G	-0.0122175	0.0568085	0.1005	0.098898	0.0237605	0.0189998				0.00174032	2.218E-12
rs7301232	A	T	A	T	-0.0077267	0.0439821	0.1668	0.170572	0.0189553	0.015				0.0014049	3.795E-08
rs7303346	T	C	T	C	0.00630435	-0.0082167	0.7052	0.71384	0.0158707	0.57				0.00115247	4.486E-08
rs73040337	T	C	T	C	-0.0074239	-0.0103504	0.7712	0.778411	0.0175851	0.75				0.00125125	2.973E-09
rs73040431	A	G	A	G	0.0120435	0.00206541	0.0763	0.076066	0.0268428	0.87				0.00197563	1.086E-09
rs7304399	A	G	A	G	0.0109316	-0.0127542	0.4606	0.443318	0.0142726	0.27				0.00104977	2.156E-25
rs73050239	T	C	T	C	-0.0128169	-0.0030967	0.817	0.815625	0.0183044	0.760001				0.00135324	2.774E-21
rs73055556	A	G	A	G	0.0111245	-0.0201619	0.1377	0.138734	0.0205319	0.34				0.00151998	2.507E-13
rs73055568	T	G	T	G	-0.0133315	-0.0042084	0.0581	0.055628	0.0311761	0.66				0.00227909	4.927E-09
rs73059315	A	G	A	G	-0.0065282	0.00061106	0.7238	0.718991	0.0157879	0.97				0.00117293	2.607E-08
rs7306755	A	G	A	G	0.018958	-0.0036266	0.2045	0.203619	0.0176465	0.74				0.00129783	2.509E-48
rs7307965	A	G	A	G	-0.0071904	-0.0138969	0.4791	0.478413	0.0143097	0.41				0.00104772	6.764E-12
rs73117392	T	C	T	C	-0.0231174	0.0174271	0.9437	0.944569	0.0311887	0.53				0.00227921	3.556E-24
rs7312511	T	C	T	C	-0.0097768	-0.0256466	0.0986	0.098929	0.0238636	0.19				0.00175515	2.548E-08
rs7313065	A	C	A	C	-0.0095791	-0.0021249	0.171	0.157051	0.0196033	0.69				0.00139263	6.04E-12

rs73141547	A	T	A	T	-0.0156094	-0.0265762	0.6554	0.65431	0.0150232	0.0580003				0.00110104	1.269E-45
rs73153727	T	C	T	C	-0.0238571	0.00620925	0.0184	0.018198	0.0530773	0.95				0.00393405	1.323E-09
rs73197422	A	G	A	G	-0.0129122	0.00314992	0.9276	0.927825	0.0274098	0.8				0.00201999	1.637E-10
rs73208982	C	G	C	G	-0.0138726	0.00119301	0.6626	0.648685	0.0149387	0.97				0.00110666	4.749E-36
rs73210523	A	G	A	G	0.0118358	0.0251878	0.1741	0.168167	0.018952	0.3				0.0013799	9.72E-18
rs73219806	A	C	A	C	0.0123988	-0.0190096	0.1664	0.167517	0.0194517	0.34				0.00140861	1.339E-18
rs73224350	A	G	A	G	0.00976036	-0.0356547	0.8618	0.866013	0.0211117	0.13				0.00151954	1.331E-10
rs73265641	A	G	A	G	0.0100487	-0.0032809	0.3122	0.31825	0.0152449	0.83				0.00112918	5.624E-19
rs7328289	T	C	T	C	-0.0070269	-0.0341145	0.7837	0.771651	0.017144	0.0379997	rs9588917	T	G	0.00127174	3.286E-08
rs7333511	A	T	A	T	0.00898807	0.0142528	0.6332	0.634962	0.0147853	0.22				0.00108589	1.26E-16
rs7336670	A	C	A	C	0.0105051	-0.0149985	0.2144	0.210238	0.0174732	0.48				0.00127513	1.747E-16
rs73400807	A	G	A	G	0.00841196	-0.0002206	0.8655	0.862639	0.0206124	0.9				0.00153362	4.128E-08
rs73456245	T	C	T	C	0.00745903	0.0106131	0.2206	0.231082	0.0169289	0.46				0.00126643	3.867E-09
rs73516862	T	C	T	C	0.010072	0.00626947	0.7296	0.722798	0.0158586	0.64				0.00117864	1.281E-17
rs7356921	T	C	T	C	-0.0148186	0.0081189	0.241	0.255631	0.0163091	0.450001				0.00122344	9.091E-34
rs73625120	A	G	A	G	0.0153176	0.00335066	0.0389	0.037103	0.0375465	0.86				0.00270616	1.509E-08
rs736281	T	C	T	C	0.00666016	-0.0121649	0.389	0.401102	0.0145167	0.49				0.00107329	5.456E-10
rs737902	A	G	A	G	-0.0085624	-0.0146631	0.2745	0.269141	0.0160774	0.25				0.00117253	2.832E-13
rs738988	A	G	A	G	-0.0160469	-0.0105171	0.7149	0.70781	0.0155968	0.25				0.00115902	1.355E-43
rs7397905	A	G	A	G	0.00988429	-0.0146481	0.8347	0.832938	0.0190438	0.52				0.00140905	2.303E-12
rs7402939	T	C	T	C	0.0067727	-0.0188712	0.3789	0.376728	0.0147866	0.22				0.00108236	3.916E-10
rs7405130	T	C	T	C	-0.0139844	-0.040656	0.149	0.155396	0.0195841	0.0259998				0.00147832	3.092E-21
rs7430651	T	C	T	C	-0.0112607	-0.0057443	0.2866	0.312877	0.0153524	0.62	rs7429735	C	T	0.0011572	2.217E-22
rs7431531	T	C	T	C	-0.0104763	-0.0108614	0.6791	0.679094	0.0152354	0.38				0.00112088	9.096E-21
rs74323354	A	G	A	G	0.0111949	0.0120519	0.9268	0.930302	0.0278687	0.649999				0.00200946	2.533E-08
rs7437726	T	G	T	G	0.0103576	-0.0115153	0.1273	0.127235	0.0213591	0.62				0.00156988	4.179E-11
rs7439403	C	G	C	G	-0.0066942	0.0421826	0.7477	0.743964	0.0162619	0.00779992				0.00121255	3.376E-08
rs74439972	A	G	A	G	0.00868783	-0.0072089	0.2666	0.283252	0.0163122	0.9				0.00120661	6.025E-13
rs7449561	A	G	A	G	0.0097312	0.00783443	0.2268	0.228467	0.0169225	0.62				0.00124952	6.829E-15
rs7452072	T	C	T	C	0.00907851	0.024714	0.8855	0.882296	0.0220073	0.39				0.00164329	3.299E-08
rs745280	A	G	A	G	0.00689516	0.00851287	0.5353	0.552363	0.0142696	0.48				0.0010499	5.124E-11
rs74532781	A	G	A	G	-0.0119196	0.0142809	0.1436	0.148317	0.0199906	0.58				0.00149422	1.495E-15
rs746839	C	G	C	G	0.00989129	-0.0129942	0.633	0.624946	0.0148917	0.3				0.00108642	8.644E-20
rs7468947	A	G	A	G	-0.0088809	-0.0189446	0.233	0.238189	0.0166367	0.28				0.00124252	8.831E-13
rs7469569	T	C	T	C	0.00953024	0.0199515	0.3575	0.365881	0.0147306	0.12				0.00109644	3.573E-18
rs74740938	A	T	A	T	0.0120845	-0.0420957	0.1186	0.117308	0.0221442	0.032				0.00161875	8.299E-14
rs74828610	T	G	T	G	-0.0125998	0.0249069	0.9263	0.920922	0.0263853	0.43				0.00200294	3.161E-10
rs74833567	A	G	A	G	0.0127896	-0.0593135	0.0976	0.095544	0.0241545	0.0140001				0.00176338	4.075E-13
rs748404	T	C	T	C	-0.0082069	0.0137862	0.7784	0.781024	0.0171156	0.32				0.00126004	7.353E-11
rs74844193	A	G	A	G	-0.0294911	-0.0542062	0.0161	0.01576	0.0569936	0.33				0.00496856	2.929E-09
rs748832	A	G	A	G	0.0100718	0.00469725	0.6314	0.635533	0.014728	0.85				0.00108463	1.608E-20
rs7517857	A	G	A	G	-0.0061716	-0.0195916	0.438	0.44027	0.0142977	0.15				0.00105465	4.856E-09
rs7520927	A	G	A	G	-0.0068431	0.0116397	0.4848	0.490932	0.0142142	0.31				0.00104729	6.382E-11
rs7523793	T	C	T	C	0.00779465	0.0161098	0.6384	0.641398	0.0148148	0.23				0.00108937	8.371E-13
rs75352055	A	T	A	T	-0.0209498	0.0141069	0.9762	0.975984	0.0464005	0.760001				0.00344671	1.217E-09
rs7543410	T	G	T	G	0.00720783	0.00082552	0.2422	0.255477	0.0162607	0.88				0.00122137	3.602E-09
rs7546304	C	G	C	G	-0.0078694	-0.005869	0.3012	0.300787	0.0154796	0.73				0.00114086	5.274E-12
rs7549469	A	T	A	T	0.00890771	0.0104314	0.6796	0.681644	0.0152585	0.52				0.00112135	1.96E-15
rs755043	A	G	A	G	-0.0063634	0.00891597	0.5115	0.507377	0.0142206	0.450001				0.00104692	1.217E-09
rs7556635	T	C	T	C	-0.0061897	-0.0036078	0.3216	0.318467	0.0152856	0.709999				0.00112024	3.291E-08
rs7559005	T	C	T	C	-0.0099311	0.00187491	0.4249	0.427382	0.014325	0.81				0.00105852	6.474E-21
rs75624576	T	G	T	G	0.0153607	-0.0058977	0.1004	0.091996	0.0247951	0.6				0.00174542	1.363E-18
rs7565063	A	T	A	T	0.00768547	-0.0049526	0.2434	0.241969	0.0166444	0.8				0.00121932	2.914E-10
rs75672140	A	C	A	C	0.0192538	-0.0658159	0.0414	0.04228	0.035762	0.0569994				0.00262683	2.304E-13
rs75687828	A	G	A	G	0.0191221	-0.017745	0.0852	0.094779	0.0245286	0.48				0.00188747	4.036E-24
rs75740317	T	C	T	C	0.0233528	0.052454	0.9764	0.976348	0.0472684	0.25				0.00346541	1.594E-11

rs757436	A	T	A	T	0.00645172	-0.0139468	0.4039	0.397034	0.0145388	0.31				0.00106731	1.493E-09
rs7578247	A	G	A	G	-0.0079832	-0.0162222	0.6377	0.655676	0.0149355	0.55	rs6430953	T	G	0.0010887	2.254E-13
rs75782645	A	G	A	G	-0.0106563	0.0131734	0.8756	0.866307	0.0210089	0.62				0.00158588	1.82E-11
rs7578633	T	C	T	C	0.00612669	-0.0111927	0.3636	0.360809	0.0147792	0.25				0.00108777	1.774E-08
rs7584255	T	C	T	C	0.00719668	-0.0308519	0.7521	0.759089	0.016608	0.05				0.00121216	2.901E-09
rs75877036	A	T	A	T	-0.0144694	-0.0119337	0.9422	0.942658	0.0307868	0.61				0.00224264	1.107E-10
rs7589741	A	T	A	T	-0.0069083	-0.0078283	0.3882	0.381139	0.0146137	0.5				0.00107369	1.244E-10
rs75915571	A	T	A	T	0.00922405	-0.0134065	0.1956	0.200346	0.0178421	0.61				0.00132755	3.695E-12
rs75931210	T	C	T	C	0.0193509	-0.0494821	0.9653	0.969737	0.0417179	0.28				0.00286069	1.336E-11
rs7597126	T	C	T	C	-0.008314	-0.0254785	0.4971	0.502644	0.0142557	0.0690001				0.00104938	2.324E-15
rs75986654	C	G	C	G	-0.0182236	0.027937	0.0372	0.037278	0.0375779	0.47				0.00276536	4.398E-11
rs7602601	T	C	T	C	-0.0104253	0.0421735	0.8808	0.883675	0.0221508	0.0280001				0.00161486	1.076E-10
rs7603132	A	G	A	G	0.0172902	0.00686597	0.1897	0.196671	0.017881	0.47				0.00133623	2.702E-38
rs76076331	T	C	T	C	0.0220599	-0.009874	0.1299	0.123513	0.0217024	0.719999				0.00155748	1.539E-45
rs7614270	A	C	A	C	-0.0101505	-0.0037619	0.1481	0.150981	0.0199156	0.709999				0.00147354	5.648E-12
rs76157199	A	G	A	G	0.0202127	-0.0969836	0.9638	0.9626	0.0373741	0.0129999				0.00282092	7.758E-13
rs7628120	T	C	T	C	0.0101265	-0.0215326	0.7023	0.71817	0.015766	0.29				0.00114673	1.042E-18
rs7632819	A	G	A	G	-0.0130068	-0.000429	0.755	0.739113	0.0161281	0.51				0.00121662	1.126E-26
rs7641534	T	C	T	C	-0.0079235	0.0286489	0.4567	0.457909	0.0142685	0.0369999				0.00105045	4.604E-14
rs76449306	T	G	T	G	-0.0105761	-0.0027024	0.8916	0.896692	0.0233958	0.91				0.00168311	3.308E-10
rs7650602	T	C	T	C	-0.0099841	-0.0041377	0.5651	0.551165	0.014354	0.780001				0.00105579	3.194E-21
rs76522376	T	C	T	C	0.00675308	0.00169163	0.7138	0.716155	0.0159255	0.99				0.00115801	5.486E-09
rs7654288	A	G	A	G	-0.0069911	-0.0080022	0.5224	0.517021	0.0141958	0.42				0.00104756	2.496E-11
rs7657329	A	C	A	C	-0.0085617	0.0113554	0.3184	0.320474	0.0153287	0.6				0.00112398	2.592E-14
rs76631016	T	C	T	C	-0.0147556	-0.0439426	0.0514	0.051744	0.0322252	0.16				0.00236989	4.779E-10
rs7668960	T	C	T	C	0.015867	0.00042651	0.1548	0.156061	0.0196211	0.99				0.0014466	5.423E-28
rs7670522	A	C	A	C	0.0126785	0.00853287	0.4727	0.455754	0.0142472	0.719999				0.00117966	6.068E-27
rs7677621	T	C	T	C	-0.0102896	0.0334658	0.3531	0.346814	0.0150151	0.025				0.00109483	5.543E-21
rs7679853	A	G	A	G	-0.0109153	-0.0060982	0.3359	0.335514	0.0150554	0.649999				0.00110819	6.87E-23
rs76829263	T	C	T	C	0.024552	-0.087209	0.0225	0.02394	0.048251	0.1				0.0035622	5.479E-12
rs76878669	C	G	C	G	0.01212	0.00464166	0.7581	0.765608	0.0168508	0.69				0.00122972	6.48E-23
rs7698820	T	C	T	C	-0.0093874	-0.0015341	0.2934	0.273237	0.016116	0.69				0.0011492	3.126E-16
rs7704018	C	G	C	G	-0.0075855	0.00774069	0.7162	0.725786	0.0159124	0.49				0.00116077	6.364E-11
rs7709041	T	G	T	G	-0.0087234	0.0378133	0.1568	0.157091	0.0194515	0.0619998				0.00143923	1.351E-09
rs77128898	T	C	T	C	-0.0242039	-0.009947	0.0334	0.035323	0.0392511	0.79				0.0029457	2.085E-16
rs7715167	T	C	T	C	-0.0082489	0.00155574	0.3883	0.389218	0.0145446	0.95				0.0010783	2.015E-14
rs7716876	T	C	T	C	-0.0106346	0.0113838	0.2795	0.275852	0.0158848	0.46				0.00116617	7.567E-20
rs77187837	T	C	T	C	0.0129263	-0.0203775	0.1223	0.125296	0.0215803	0.649999				0.00159729	5.827E-16
rs7721182	T	C	T	C	0.0067273	-0.0217418	0.4433	0.446098	0.0142786	0.14				0.00105344	1.702E-10
rs7729431	A	G	A	G	0.00662683	-0.0035198	0.4826	0.473413	0.0142452	0.69				0.00104737	2.493E-10
rs77366811	T	G	T	G	-0.0193092	0.0490122	0.0265	0.027074	0.0439846	0.17				0.00328794	4.292E-09
rs77423896	A	G	A	G	-0.0151565	0.0534809	0.9465	0.948994	0.0322385	0.14				0.00232593	7.194E-11
rs7749979	T	C	T	C	-0.0068482	-0.022389	0.7073	0.708727	0.0156242	0.12				0.00115	2.601E-09
rs7750668	T	G	T	G	0.00791108	-0.0132455	0.3203	0.319844	0.0153235	0.43				0.00112144	1.737E-12
rs7754547	T	G	T	G	-0.0068031	-0.0020021	0.6967	0.689956	0.0153333	0.95				0.00114065	2.454E-09
rs77584294	A	C	A	C	0.0259131	-0.0277437	0.0399	0.049353	0.0329001	0.77				0.0026783	3.857E-22
rs7762296	T	C	T	C	-0.0083505	-0.0130009	0.305	0.296661	0.0155934	0.450001				0.0011365	2.018E-13
rs77702819	T	G	T	G	0.0157641	0.0139781	0.0895	0.093642	0.0246758	0.52				0.00184055	1.084E-17
rs7774177	A	G	A	G	-0.0060077	0.0273206	0.4476	0.434565	0.014324	0.0649995				0.0010523	1.138E-08
rs7775100	T	C	T	C	-0.008142	0.00707433	0.3913	0.392159	0.0145509	0.7				0.00107215	3.093E-14
rs7780267	A	C	A	C	-0.0058768	0.00437771	0.4122	0.415095	0.0144235	0.64				0.00106674	3.612E-08
rs77882218	T	C	T	C	-0.0256345	-0.0001595	0.9716	0.972845	0.0441631	0.75				0.00315042	4.061E-16
rs7796089	C	G	C	G	-0.0082703	-0.0172898	0.3381	0.347634	0.0148733	0.26				0.0011061	7.594E-14
rs77980141	C	G	C	G	-0.0269629	-0.046017	0.9882	0.987235	0.0635771	0.4				0.00487233	3.137E-08
rs7803932	A	G	A	G	0.0116905	0.0381243	0.163	0.171108	0.0190022	0.015				0.00141663	1.549E-16
rs78102134	A	G	A	G	0.010901	-0.0114743	0.0872	0.085878	0.0254333	0.51				0.00186162	4.756E-09

rs7810903	A	C	A	C	0.0119349	-0.0242258	0.306	0.309549	0.0153638	0.18				0.00113546	7.663E-26
rs78116078	C	G	C	G	0.00930277	-0.0165979	0.7184	0.714181	0.0157183	0.34				0.00116336	1.282E-15
rs7814757	T	C	T	C	-0.0072908	0.0306587	0.4013	0.392394	0.0145581	0.0819993				0.00106765	8.583E-12
rs7815299	C	G	C	G	0.00777551	-0.0146531	0.6699	0.675829	0.0151726	0.5				0.00111286	2.814E-12
rs7825105	A	G	A	G	-0.006967	-0.0010238	0.2775	0.29067	0.0156445	0.89				0.00116874	2.509E-09
rs78278641	T	C	T	C	0.0122435	-0.0045959	0.1373	0.137497	0.0205971	0.66				0.00152933	1.185E-15
rs7839989	T	C	T	C	0.00987508	0.0140776	0.2241	0.230791	0.0168174	0.38				0.001255	3.58E-15
rs78410329	T	C	T	C	0.01783	0.0183974	0.2242	0.223026	0.0171019	0.24				0.00125925	1.632E-45
rs7842016	C	G	C	G	0.00783795	-0.0164919	0.7254	0.734517	0.0161093	0.35				0.00117255	2.312E-11
rs78462816	A	G	A	G	-0.0103679	0.0308836	0.8806	0.871713	0.0213757	0.12				0.00161647	1.419E-10
rs7847405	T	G	T	G	0.00928355	-0.0023262	0.6495	0.659816	0.0149716	0.93				0.00109682	2.576E-17
rs785113	T	C	T	C	0.00739059	-0.0143433	0.2265	0.2403	0.0166923	0.51				0.00125086	3.447E-09
rs785490	T	C	T	C	0.00809267	0.0119514	0.2899	0.295314	0.0155383	0.54				0.00115326	2.262E-12
rs7855503	C	G	C	G	0.00674521	0.00347613	0.3465	0.353795	0.0148398	0.75				0.00110019	8.747E-10
rs78631600	T	G	T	G	-0.0180874	-0.0056377	0.0783	0.079976	0.0263404	0.9				0.00194831	1.636E-20
rs78667595	A	G	A	G	-0.0124509	-0.0462705	0.0917	0.091469	0.0248643	0.0560003				0.00181855	7.555E-12
rs78689892	T	C	T	C	-0.0154114	-0.0416647	0.9608	0.964266	0.0384141	0.33				0.00269797	1.116E-08
rs7873029	T	C	T	C	-0.0129487	-0.0286317	0.0583	0.058135	0.0303405	0.33				0.00223469	6.86E-09
rs78873173	A	G	A	G	0.014319	-0.0275094	0.0811	0.086975	0.0251747	0.32				0.00192205	9.334E-14
rs7905026	A	C	A	C	-0.0074765	-0.0121262	0.2604	0.259657	0.0161733	0.51				0.00119248	3.619E-10
rs79109558	T	C	T	C	0.0200717	0.0288291	0.0237	0.021965	0.048685	0.42				0.00344095	5.449E-09
rs7911488	A	G	A	G	-0.011714	0.016692	0.6723	0.682666	0.0152357	0.22				0.00111493	8.076E-26
rs792213	A	C	A	C	0.00732975	-0.0060442	0.4133	0.415603	0.0144128	0.64				0.00106274	5.31E-12
rs7924036	T	G	T	G	0.0141206	0.0226493	0.5115	0.503299	0.0141941	0.17				0.00104692	1.856E-41
rs7924465	T	C	T	C	0.0144836	0.0310435	0.9068	0.906719	0.0244344	0.2				0.0017999	8.495E-16
rs7928622	A	T	A	T	-0.007947	0.0159939	0.6768	0.680535	0.0152558	0.29				0.00111878	1.221E-12
rs79303419	T	C	T	C	-0.0131048	0.0254298	0.9458	0.941493	0.0308591	0.46				0.00233472	1.988E-08
rs7933521	T	C	T	C	0.00825515	-0.0173597	0.8553	0.861444	0.0205544	0.21				0.00148737	2.851E-08
rs79409011	C	G	C	G	0.0146107	-0.0021389	0.0644	0.066955	0.028341	0.92				0.00213763	8.207E-12
rs79454302	T	C	T	C	-0.0146603	0.0226895	0.0446	0.046667	0.0336366	0.42				0.00254403	8.283E-09
rs7945465	A	C	A	C	-0.0074227	0.0140871	0.2855	0.281428	0.0157918	0.46				0.00115853	1.482E-10
rs795540	T	C	T	C	-0.0079762	-0.0016996	0.5854	0.581881	0.0144626	0.9				0.00106225	5.952E-14
rs79556505	T	C	T	C	-0.0130978	0.00850513	0.8995	0.899436	0.023724	0.87				0.00174128	5.391E-14
rs79587027	A	T	A	T	0.0122047	0.0297898	0.9377	0.935371	0.0293911	0.31				0.00217377	1.973E-08
rs7959047	A	G	A	G	-0.0135117	-0.0351327	0.053	0.051385	0.0323721	0.24				0.00233654	7.352E-09
rs79653698	A	G	A	G	0.022695	-0.0544383	0.9784	0.976796	0.047157	0.22				0.00363145	4.124E-10
rs7965875	T	C	T	C	-0.0089008	-0.0047417	0.7018	0.710355	0.0159081	0.760001				0.00114564	7.891E-15
rs7968682	T	G	T	G	-0.0061424	-0.0146578	0.5211	0.517858	0.0142468	0.17				0.00104744	4.51E-09
rs7975065	A	G	A	G	0.00796413	0.00024625	0.3058	0.3123	0.0153476	0.9				0.00113834	2.63E-12
rs79754409	A	T	A	T	0.00774372	-0.0127746	0.8039	0.814524	0.0184407	0.54				0.00131824	4.251E-09
rs79766835	C	G	C	G	0.0124051	0.0193483	0.0969	0.100391	0.0239333	0.4				0.00176957	2.377E-12
rs7977614	A	G	A	G	-0.0111541	0.011983	0.7172	0.708017	0.015579	0.61				0.00117652	2.528E-21
rs7979979	T	C	T	C	-0.0116479	-0.0026721	0.2438	0.253563	0.0163405	0.94				0.00121864	1.198E-21
rs7981839	A	C	A	C	0.00825394	-0.0109472	0.8194	0.823859	0.0186292	0.59				0.00136039	1.297E-09
rs7983020	T	C	T	C	-0.0089294	0.0147099	0.7151	0.710442	0.0156516	0.41				0.00115926	1.336E-14
rs79838927	A	G	A	G	0.0103155	-0.0847544	0.9057	0.901444	0.0240594	0.00017				0.00180807	1.163E-08
rs79844375	T	C	T	C	-0.0113033	0.025832	0.0994	0.097022	0.0242692	0.31				0.00175297	1.135E-10
rs7986948	T	G	T	G	0.00878503	-0.0022015	0.1261	0.128371	0.0213529	0.92				0.00157645	2.505E-08
rs7993752	A	C	A	C	0.00818828	-0.0052608	0.5793	0.585845	0.014537	0.760001				0.00106006	1.122E-14
rs799443	A	T	A	T	-0.0109125	0.0135827	0.6654	0.671944	0.015103	0.48	rs799447	T	G	0.00111131	9.302E-23
rs799809	A	T	A	T	-0.006512	0.00516495	0.7048	0.695312	0.0154524	0.89				0.00114953	1.473E-08
rs79994966	T	C	T	C	-0.0079152	-0.0033727	0.6087	0.606549	0.0145214	0.99				0.00107229	1.561E-13
rs7999579	T	C	T	C	-0.0070969	0.0193437	0.7706	0.775129	0.0169775	0.2				0.00125226	1.451E-08
rs8000862	A	G	A	G	-0.00679	-0.0138914	0.3587	0.372009	0.0147173	0.55				0.00109122	4.894E-10
rs8006699	A	G	A	G	0.00962181	-0.0341856	0.8263	0.819475	0.0184988	0.0350002				0.00138171	3.322E-12
rs80090296	T	C	T	C	-0.0083715	0.00212594	0.1693	0.165537	0.01916	0.91				0.00139528	1.977E-09

rs8012614	A	C	A	C	-0.0078889	0.0138568	0.4996	0.485234	0.0142082	0.42				0.00104867	5.351E-14
rs8015454	T	C	T	C	0.0170762	-0.0101752	0.9522	0.955772	0.0344778	0.99				0.00245436	3.461E-12
rs801563	T	C	T	C	-0.0096592	-0.0157028	0.2112	0.213322	0.0173704	0.3				0.00128215	4.948E-14
rs80159981	T	C	T	C	0.00919473	0.0350619	0.8576	0.852421	0.0200877	0.0759994				0.00149783	8.304E-10
rs80171383	A	G	A	G	0.0146151	0.0326595	0.1355	0.144531	0.0202342	0.0589997				0.00152883	1.182E-21
rs8017691	A	C	A	C	0.00734847	-0.0057894	0.4257	0.431559	0.0143729	0.59				0.00105856	3.857E-12
rs8018636	T	C	T	C	0.00955255	-0.0029979	0.8858	0.894164	0.0231656	0.98				0.00164858	6.85E-09
rs8020023	A	G	A	G	0.0144509	-0.0315737	0.2379	0.238645	0.0166876	0.0629999				0.00122888	6.336E-32
rs8028148	A	G	A	G	0.00728669	-0.0054254	0.7024	0.69681	0.015471	0.82				0.00114815	2.205E-10
rs80322007	T	C	T	C	-0.0075204	-0.0020641	0.7688	0.767637	0.0167938	0.93				0.00127383	3.547E-09
rs80352808	A	G	A	G	-0.0075073	-0.0150877	0.2808	0.279563	0.0158596	0.2				0.00116469	1.148E-10
rs8043948	A	G	A	G	-0.0062871	-0.0050593	0.482	0.492128	0.0142422	0.88				0.00104732	1.941E-09
rs8044082	T	C	T	C	0.0071805	0.0169617	0.3995	0.396373	0.0146025	0.31				0.00106874	1.833E-11
rs8044562	A	G	A	G	-0.0119271	0.0124014	0.2846	0.289886	0.0156254	0.23				0.00115978	8.335E-25
rs8048012	A	G	A	G	0.00821599	-0.0052041	0.1729	0.167997	0.0190442	0.69				0.0013871	3.154E-09
rs8055491	A	G	A	G	-0.0077639	-0.0024324	0.4648	0.466118	0.0142245	0.88				0.00104925	1.364E-13
rs8055522	T	C	T	C	0.00981556	-0.0191062	0.3373	0.312563	0.015807	0.14				0.00112745	3.149E-18
rs8058137	A	G	A	G	0.0146556	-0.0297658	0.7827	0.796787	0.0177189	0.16				0.00127033	8.608E-31
rs8060784	T	C	T	C	0.00753632	-0.0133797	0.2713	0.274473	0.0159486	0.49				0.00117698	1.525E-10
rs8063312	A	C	A	C	-0.0104702	-0.0108532	0.1136	0.110088	0.0226507	0.49	rs8063812	A	G	0.00165258	2.367E-10
rs8067165	C	G	C	G	-0.0062936	0.00658597	0.3993	0.398847	0.014622	0.83				0.00107252	4.41E-09
rs8072494	A	G	A	G	-0.0115965	0.00437923	0.7895	0.787312	0.0173653	0.85				0.00128354	1.647E-19
rs8073654	T	C	T	C	0.00700877	-0.0065064	0.567	0.569587	0.0145102	0.62				0.00105894	3.631E-11
rs8079766	T	C	T	C	0.00690669	0.00262004	0.3945	0.395567	0.0145209	0.91				0.00107061	1.108E-10
rs826374	A	C	A	C	-0.0089116	-0.0020004	0.7718	0.776091	0.0170358	0.98				0.00124681	8.819E-13
rs827951	A	G	A	G	-0.0099024	0.0228326	0.731	0.732502	0.0160253	0.17				0.00117999	4.778E-17
rs831229	A	G	A	G	0.00712466	-0.0050108	0.3729	0.363669	0.014781	0.66				0.00108254	4.65E-11
rs837080	T	C	T	C	-0.0097736	-0.0030061	0.5225	0.531884	0.0142228	0.84	rs401526	C	T	0.00104771	1.074E-20
rs838041	T	C	T	C	-0.0082224	-0.0120807	0.352	0.349386	0.0149135	0.51				0.0010956	6.157E-14
rs852000	T	C	T	C	0.00773556	0.00233089	0.4688	0.469435	0.0142955	0.94				0.00104894	1.648E-13
rs853199	T	C	T	C	0.00859493	0.00210151	0.355	0.357763	0.0148002	0.89				0.0010935	3.837E-15
rs853345	A	G	A	G	0.00695453	0.0022058	0.2452	0.234568	0.0167558	0.98				0.00121881	1.159E-08
rs8614	A	C	A	C	-0.0127362	0.0294621	0.1872	0.182474	0.0183944	0.0810009				0.00134439	2.697E-21
rs862957	A	T	A	T	0.00774157	0.00803071	0.2456	0.24792	0.0165356	0.69				0.00121611	1.942E-10
rs863006	A	G	A	G	-0.0098396	-0.0026602	0.5624	0.563976	0.0143858	0.96				0.00105724	1.313E-20
rs872602	A	G	A	G	-0.012586	-0.0010426	0.2405	0.24094	0.0166355	0.92				0.00122719	1.113E-24
rs881562	T	G	T	G	0.00605383	-0.0128059	0.5086	0.506636	0.0144263	0.21				0.00104983	8.093E-09
rs886942	T	C	T	C	-0.0128766	0.00148672	0.8747	0.871018	0.0211776	0.68				0.00158055	3.737E-16
rs895204	A	G	A	G	-0.0123373	-0.0285434	0.133	0.12906	0.0212721	0.16				0.00154105	1.185E-15
rs903981	T	G	T	G	-0.0071521	-0.0247868	0.4122	0.403909	0.0144642	0.0490004				0.00106316	1.733E-11
rs905993	C	G	C	G	-0.0096169	0.00018511	0.3892	0.393753	0.0145699	0.719999				0.00107319	3.21E-19
rs912609	T	G	T	G	0.0123495	-0.0474866	0.2251	0.229144	0.0169262	0.00879995				0.00125321	6.574E-23
rs9267673	T	C	T	C	-0.0209133	-0.0208867	0.0902	0.090424	0.0247153	0.35				0.00182859	2.732E-30
rs9267812	T	C	T	C	-0.0123153	0.0269958	0.1338	0.135951	0.020689	0.19				0.00154563	1.618E-15
rs9276822	A	C	A	C	0.01295	0.0408583	0.101	0.108776	0.0227953	0.0309999				0.00173739	9.082E-14
rs9291467	T	C	T	C	-0.0076594	0.0355766	0.4587	0.452142	0.0142831	0.0179999				0.0010501	3.01E-13
rs9294420	A	C	A	C	-0.0061593	0.0172105	0.5815	0.581145	0.0144133	0.23				0.00106069	6.355E-09
rs9297016	A	G	A	G	0.0112169	-0.0069048	0.3986	0.402583	0.0144735	0.69				0.00106881	9.131E-26
rs9317202	T	C	T	C	0.00978591	0.0247949	0.7027	0.7155	0.0157277	0.0680002				0.00114495	1.266E-17
rs9319011	A	G	A	G	0.00895672	0.00319361	0.8317	0.838556	0.0192787	0.69				0.00140562	1.867E-10
rs9320991	T	C	T	C	0.00935415	0.0168666	0.1974	0.19923	0.0177954	0.24				0.00131459	1.112E-12
rs9321394	A	G	A	G	0.00919212	0.0225007	0.2485	0.244369	0.0165422	0.16				0.00121118	3.215E-14
rs933738	A	G	A	G	-0.0124411	0.00582316	0.8199	0.8269	0.0187874	0.85				0.0013645	7.666E-20
rs9356034	T	C	T	C	0.0103382	0.0165434	0.6834	0.678703	0.0153367	0.3				0.00112724	4.673E-20
rs9356513	A	C	A	C	-0.0085021	0.00177092	0.7076	0.706279	0.0156107	0.81				0.00121713	2.844E-12
rs9359939	A	C	A	C	-0.0108478	0.0217984	0.2421	0.243336	0.0168405	0.2				0.00122154	6.645E-19

rs9367942	A	G	A	G	0.0095457	0.0241128	0.1401	0.134704	0.0210562	0.34				0.00151129	2.681E-10
rs9372526	A	C	A	C	-0.0079791	0.00269307	0.2582	0.259886	0.0161865	0.87				0.00119643	2.577E-11
rs9372734	T	C	T	C	0.0219147	-0.0130617	0.4819	0.482374	0.0142246	0.39				0.00104729	3.15E-97
rs9373363	A	G	A	G	-0.0106184	-0.0025869	0.7552	0.747427	0.0164596	0.98				0.00121696	2.662E-18
rs9375711	A	T	A	T	0.00615987	-0.0045398	0.3241	0.333614	0.0151743	0.93				0.00111829	3.629E-08
rs9381086	C	G	C	G	-0.0085033	0.00687497	0.4002	0.400528	0.0145705	0.41				0.00106846	1.746E-15
rs938454	T	G	T	G	-0.007141	0.0177708	0.3931	0.400239	0.0145103	0.12				0.00107364	2.91E-11
rs9384761	A	G	A	G	-0.0092735	-0.0153987	0.2411	0.247428	0.0164314	0.36				0.00122327	3.425E-14
rs9388304	A	G	A	G	0.00598462	-0.017827	0.6289	0.626324	0.0147711	0.2				0.00108312	3.286E-08
rs9388490	T	C	T	C	0.0115001	-0.0211378	0.4488	0.439998	0.0143199	0.0940005				0.00105233	8.432E-28
rs9393415	T	C	T	C	0.0138342	-0.008873	0.6909	0.702052	0.0156638	0.709999				0.0011326	2.603E-34
rs940052	A	G	A	G	0.00595962	-0.0108801	0.4437	0.447996	0.0142844	0.48				0.00105825	1.788E-08
rs9400864	T	C	T	C	0.00801995	-0.003803	0.6792	0.687343	0.0152899	0.95				0.00112098	8.418E-13
rs9409514	T	C	T	C	-0.0089142	-0.0112949	0.3499	0.365554	0.014751	0.48				0.00110004	5.336E-16
rs9411336	T	C	T	C	-0.0128496	0.0258578	0.3234	0.320098	0.0152106	0.12				0.00111875	1.559E-30
rs9411446	A	G	A	G	0.00667352	0.00213086	0.4055	0.408236	0.0144217	0.61				0.00106585	3.815E-10
rs9427388	T	C	T	C	-0.0127609	0.0503509	0.0804	0.07351	0.0275007	0.13				0.00193369	4.123E-11
rs9454264	T	C	T	C	0.00777009	0.0033529	0.4693	0.468026	0.0142453	0.74				0.00105075	1.418E-13
rs9461242	T	C	T	C	-0.013624	0.006547	0.3655	0.372706	0.0146536	0.58				0.00108656	4.606E-36
rs946516	T	C	T	C	0.00623133	0.00203556	0.6528	0.640354	0.0148363	0.85				0.00109923	1.438E-08
rs9465509	A	G	A	G	-0.0079007	0.00876872	0.4815	0.483412	0.0142034	0.6				0.00104722	4.552E-14
rs947091	A	G	A	G	-0.0078148	0.00852625	0.4799	0.48334	0.014256	0.74				0.00104779	8.753E-14
rs9492436	T	C	T	C	-0.0095382	-0.0062138	0.1027	0.101011	0.0235804	0.760001				0.00172384	3.149E-08
rs9496630	A	T	A	T	-0.0065728	0.0060056	0.2892	0.288692	0.0156573	0.75				0.00115474	1.257E-08
rs9516771	A	G	A	G	-0.0174302	0.0560674	0.9372	0.941221	0.0301497	0.0359998				0.00215963	6.992E-16
rs952062	A	T	A	T	0.0102289	-0.0186887	0.1157	0.117157	0.0236522	0.47				0.00168589	1.301E-09
rs9538699	A	G	A	G	0.00747466	0.0251614	0.2603	0.258695	0.0162198	0.16	rs9528137	C	A	0.00119263	3.678E-10
rs9540731	T	C	T	C	0.0107463	-0.0113743	0.5052	0.520048	0.0142006	0.630001				0.0010467	9.927E-25
rs9540920	A	G	A	G	0.0058384	0.019178	0.5713	0.568334	0.0143298	0.11				0.00105755	3.378E-08
rs9545395	T	C	T	C	0.0100749	0.0349932	0.8663	0.872244	0.0212543	0.0479999				0.00153769	5.69E-11
rs9551887	T	C	T	C	-0.0079994	-0.0004785	0.549	0.55082	0.0142836	0.97				0.00105208	2.879E-14
rs9555651	T	C	T	C	0.0110618	-0.0182523	0.1673	0.166895	0.0190411	0.52	rs9555658	C	G	0.00140209	3.029E-15
rs9560707	A	T	A	T	0.0101608	-0.0049553	0.7	0.700014	0.0155329	0.68				0.00114198	5.693E-19
rs9563130	A	T	A	T	-0.0059548	0.00535047	0.6222	0.62295	0.0147123	0.64				0.00107968	3.485E-08
rs9565961	T	C	T	C	-0.0118349	-0.0108606	0.1569	0.157255	0.0195083	0.47				0.00143886	1.948E-16
rs9569211	A	T	A	T	0.00885638	-0.0261558	0.3532	0.35322	0.0148365	0.0470002				0.0010949	6.035E-16
rs9576167	T	C	T	C	0.0060621	-0.0172035	0.5802	0.581466	0.0144003	0.19				0.00106067	1.096E-08
rs9597852	A	G	A	G	0.0115168	-0.015208	0.1052	0.101335	0.0235209	0.28				0.00170568	1.458E-11
rs9599352	A	G	A	G	-0.0101096	0.00363019	0.4683	0.464739	0.0142538	0.64				0.00104885	5.479E-22
rs9599628	T	C	T	C	0.00647864	0.0160158	0.3607	0.374899	0.0146815	0.19				0.00108979	2.761E-09
rs961450	A	G	A	G	0.0120471	-0.0014166	0.9391	0.939936	0.0298999	0.84				0.002188	3.663E-08
rs9635366	A	G	A	G	0.016096	0.0187728	0.183	0.18056	0.0184628	0.33				0.0013575	1.978E-32
rs964039	T	C	T	C	0.00654717	0.00593475	0.7203	0.717395	0.0157536	0.9				0.00118693	3.466E-08
rs9640815	C	G	C	G	-0.0092526	0.045186	0.8021	0.805575	0.0179723	0.0064				0.00131333	1.849E-12
rs9652254	A	G	A	G	0.00649352	-0.0091678	0.5881	0.585431	0.0145353	0.4				0.00106358	1.027E-09
rs9663072	A	G	A	G	-0.0066649	0.00929786	0.6985	0.703437	0.0155354	0.59				0.00114031	5.081E-09
rs971136	A	G	A	G	-0.0089353	0.00079146	0.622	0.614155	0.0147829	0.87				0.00108828	2.208E-16
rs9729347	T	C	T	C	-0.006823	0.028096	0.6914	0.691845	0.0153952	0.0379997				0.00113279	1.71E-09
rs975303	A	G	A	G	-0.0147672	-0.0056871	0.8265	0.818842	0.0184688	0.82				0.00138179	1.17E-26
rs9764	T	C	T	C	0.0111651	-0.0201951	0.7306	0.73369	0.0160368	0.19				0.00118661	4.993E-21
rs9787076	A	C	A	C	-0.0183804	-0.0010219	0.6711	0.66983	0.0151217	0.96				0.00111375	3.471E-61
rs9789595	T	C	T	C	0.013768	-0.0021758	0.6081	0.615355	0.014661	0.86				0.00107186	9.162E-38
rs9802100	A	G	A	G	0.00678238	-0.014202	0.5124	0.514587	0.0143806	0.32				0.0010521	1.146E-10
rs9804142	T	C	T	C	0.00726925	0.00982467	0.6984	0.696145	0.0154536	0.37				0.0011401	1.816E-10
rs9807112	T	C	T	C	-0.0084813	-0.0105721	0.6859	0.683348	0.0153391	0.31				0.00112742	5.365E-14
rs9808993	A	G	A	G	0.00861299	-0.0190697	0.2012	0.209932	0.0175295	0.27				0.00130914	4.734E-11

rs9809578	A	G	A	G	0.00766058	0.0227651	0.4423	0.448195	0.0143978	0.19				0.00105384	3.622E-13
rs981949	A	C	A	C	0.00932843	0.0176905	0.675	0.673311	0.0151804	0.22				0.00111717	6.82E-17
rs9827101	A	C	A	C	-0.0083622	-0.0315492	0.5282	0.517275	0.0142477	0.021				0.00104818	1.485E-15
rs9844381	A	G	A	G	0.00759303	-0.0142495	0.8138	0.803402	0.017906	0.5	rs9846740	A	T	0.00134477	1.639E-08
rs9849462	T	C	T	C	0.00705516	-0.0104691	0.2959	0.303534	0.0155144	0.55				0.00114669	7.623E-10
rs9857331	A	G	A	G	-0.0081214	-0.0287582	0.7294	0.733001	0.0160513	0.0769999	rs4857697	T	C	0.00117778	5.369E-12
rs9859719	A	G	A	G	-0.0112818	0.0180249	0.2223	0.23018	0.0168627	0.2				0.00126106	3.673E-19
rs9869597	A	T	A	T	-0.0087341	0.00744951	0.7858	0.801218	0.017816	0.59				0.0012754	7.462E-12
rs9871964	T	C	T	C	-0.009507	-0.0103327	0.1745	0.17628	0.0187739	0.630001				0.00137905	5.42E-12
rs9875	T	C	T	C	-0.0069375	0.00025936	0.6886	0.683752	0.0152665	0.85	rs13044950	C	T	0.00112998	8.287E-10
rs9875943	A	G	A	G	0.0100211	-0.0195012	0.1097	0.103629	0.0233541	0.44				0.00167433	2.157E-09
rs9880138	T	C	T	C	-0.0103951	-0.0137228	0.9082	0.910317	0.0250576	0.630001				0.00181218	9.655E-09
rs9886703	A	T	A	T	-0.0112291	0.00431352	0.1671	0.167908	0.0190723	0.86				0.00140316	1.22E-15
rs9888376	T	C	T	C	-0.005883	0.0039021	0.5713	0.572952	0.0143762	0.89				0.00105731	2.634E-08
rs988875	A	G	A	G	-0.0064467	0.00134501	0.6619	0.674596	0.0151989	0.91				0.0011062	5.63E-09
rs9888861	A	T	A	T	-0.0084795	0.00720745	0.7509	0.757064	0.0166999	0.62				0.00121335	2.779E-12
rs9891803	T	C	T	C	-0.0094776	-0.0014797	0.4965	0.489173	0.0142518	0.760001				0.00104848	1.57E-19
rs9899056	A	G	A	G	0.00698498	-0.0215606	0.3994	0.39243	0.0145236	0.15				0.00107014	6.71E-11
rs989996	T	C	T	C	-0.0094157	-0.0066458	0.4608	0.454886	0.0143523	0.58				0.00104974	2.978E-19
rs991001	C	G	C	G	0.00979859	0.0115456	0.1345	0.127018	0.0213259	0.719999				0.00153382	1.674E-10
rs9914251	A	G	A	G	0.00769663	0.00688685	0.7579	0.756049	0.0165586	0.66				0.00122154	2.967E-10
rs9915323	A	T	A	T	-0.0113425	0.0171646	0.2934	0.282099	0.0158199	0.51				0.0011493	5.671E-23
rs9920297	T	C	T	C	0.00789548	-0.0273176	0.6338	0.639553	0.0149617	0.0749998				0.00108732	3.84E-13
rs9922098	A	G	A	G	0.00800585	-0.0035144	0.5234	0.53127	0.0142651	0.9				0.00105068	2.536E-14
rs9922788	A	G	A	G	0.0102792	0.00531192	0.4337	0.447357	0.0142824	0.709999				0.00105583	2.121E-22
rs9926032	T	C	T	C	-0.0057825	-0.0012004	0.5587	0.557607	0.0143052	0.92				0.00105393	4.105E-08
rs9928897	T	C	T	C	-0.0126734	-0.0100118	0.0666	0.067036	0.02876	0.719999				0.00209925	1.566E-09
rs9930253	A	T	A	T	-0.0098564	0.016683	0.2786	0.282896	0.0157928	0.35				0.0011675	3.119E-17
rs9937449	T	C	T	C	0.00777199	0.00174921	0.5553	0.563675	0.0143329	0.82				0.00105311	1.583E-13
rs9946776	A	C	A	C	-0.0059085	0.00598136	0.3909	0.397543	0.0145263	0.74				0.00107235	3.592E-08
rs9965170	A	G	A	G	0.0096514	-0.0344908	0.4297	0.423886	0.0143654	0.0179999				0.00105701	6.771E-20
rs9969765	C	G	C	G	-0.0064665	0.00755574	0.34	0.339175	0.0150044	0.44				0.00110549	4.925E-09
rs9972455	A	G	A	G	0.00591269	0.00330962	0.3994	0.408657	0.0146548	0.69				0.0010701	3.282E-08
rs9976560	C	G	C	G	0.00845015	0.0342557	0.7497	0.756653	0.0165531	0.0140001				0.00120808	2.661E-12
rs9984313	T	C	T	C	-0.0078108	-0.0205767	0.7582	0.763932	0.0167683	0.34				0.00122222	1.649E-10
rs9985296	T	C	T	C	-0.0103135	0.0104471	0.3825	0.374612	0.0146401	0.39				0.00107676	9.856E-22
rs9992967	T	G	T	G	0.00820093	-0.0018553	0.786	0.788343	0.0174298	0.88				0.00127583	1.291E-10
rs9993049	T	C	T	C	-0.0071695	0.0121086	0.6201	0.611626	0.0147276	0.35				0.00107867	3.004E-11
rs9993133	T	C	T	C	0.00760777	0.0321041	0.8258	0.828932	0.0188722	0.0729995				0.00137959	3.496E-08
rs9998726	T	C	T	C	-0.006284	0.00286797	0.4311	0.44027	0.0142951	0.92				0.00106302	3.396E-09

SNP, single nucleotide polymorphism; EA, effect allele; OA, other allele; EAF, effect allele frequency; SE, standard error; Edu, educational attainment

Supplementary list S2: Single nucleotide polymorphisms (SNPs) included in the multivariable MR analysis

SNP	Beta Edu	SE Edu	Beta Alc	SE Alc	Beta Smk	SE Smk	Beta BMI	SE BMI	Beta T2DM	SE T2DM	Beta HDL	SE HDL	Beta SBP	SE SBP	Beta Sepsis	SE Sepsis
rs10033587	0.0219828	0.00348121	0.0127	0.006	-0.00522	0.005	-0.0179	0.0057	-0.0193	0.0211	-0.01	0.0163	0.0655	0.0982	-0.0544006	0.0466532
rs1007731	-0.0112959	0.00162501	0.000032	0.003	0.00588	0.003	-0.0038	0.0028	0.0089	0.0105	-0.0051	0.0088	0.0634	0.0489	-0.0106124	0.0221907
rs10180845	-0.0095958	0.00108643	0.000697	0.002	0.00191	0.002	0.0093	0.0018	0	0.0067	-0.005	0.0052	0.0521	0.0314	0.0111018	0.014841
rs10189857	0.0137772	0.00105695	0.00478	0.002	-0.000723	0.002	-0.0064	0.0017	0.0234	0.0064	-0.0031	0.0049	0.0241	0.0305	0.00090831	0.014343
rs10206079	0.00730639	0.00109189	0.00227	0.002	-0.000691	0.002	-0.0024	0.0018	0.0065	0.0066	-0.0015	0.0052	-0.0114	0.0315	-0.0090011	0.0149045
rs10241183	-0.0101267	0.00109291	-0.00381	0.002	-0.00653	0.002	-0.01	0.0018	0.0026	0.0067	-0.0031	0.0052	0.0198	0.0315	0.0289449	0.0148304
rs10256396	0.00654276	0.00110635	0.00233	0.002	-0.00271	0.002	-0.0024	0.0019	-0.0023	0.0068	-0.0022	0.0054	0.0234	0.0325	0.0152145	0.0151451
rs10283803	-0.0057542	0.0010473	-0.0048	0.002	-0.000917	0.002	0.0039	0.0017	0.0015	0.0064	-0.0002	0.0048	0.088	0.0299	-0.0095237	0.0142043
rs10415488	-0.0060192	0.00107864	-0.00135	0.002	0.00479	0.002	-0.0023	0.0018	-0.0021	0.0067	-0.0066	0.0056	-0.0158	0.0316	-0.0122507	0.0147128
rs1043595	0.0166252	0.00117629	-0.00233	0.002	-0.00341	0.002	-0.0031	0.002	0.0052	0.0072	-0.0054	0.0061	-0.0985	0.034	-0.0041897	0.0158201
rs10481106	-0.0123656	0.00143998	-0.00292	0.002	0.0037	0.002	-0.0004	0.0024	0.0184	0.0088	-0.0097	0.0065	0.0134	0.0417	-0.0151983	0.0198803
rs10496632	0.0111374	0.00116062	-0.00195	0.002	-0.0024	0.002	0.0044	0.0019	0.0033	0.007	-0.0097	0.0053	0.0027	0.0335	7.5796E-05	0.0159105
rs10507085	-0.0122372	0.00199826	-0.00254	0.003	0.00754	0.003	-0.001	0.0033	-0.005	0.0119	-0.0165	0.0096	-0.039	0.0577	-0.0192434	0.0270821
rs1056010	-0.0099782	0.00107133	-0.00997	0.002	0.00678	0.002	0.0115	0.0018	-0.0175	0.0069	-0.007	0.005	0.0067	0.0308	0.00933809	0.0144188
rs1058790	-0.0139612	0.00136396	0.00439	0.002	0.00949	0.002	0.0069	0.0023	0.0034	0.0086	-0.0148	0.0065	0.0801	0.0407	0.00072335	0.0189962
rs10740572	-0.008442	0.00125781	-0.00223	0.002	0.000304	0.002	0.0006	0.0021	-0.0142	0.0076	-0.0027	0.0058	-0.0225	0.0362	-0.0280189	0.0171839
rs10748559	-0.0065618	0.00108817	0.00122	0.002	0.00292	0.002	-0.0003	0.0018	0.0014	0.0068	-0.009	0.0058	-0.0288	0.032	0.00571614	0.0145745
rs10765789	0.0107781	0.00107655	0.000025	0.002	-0.0022	0.002	0.0004	0.0018	0.0006	0.0066	-0.0012	0.0049	0.0067	0.031	0.0233523	0.0146191
rs10786823	-0.0091788	0.00104815	0.00104	0.002	0.00417	0.002	0.0018	0.0017	-0.0052	0.0064	-0.0018	0.0051	0.0294	0.0302	0.00299463	0.0142259
rs10809464	0.00924129	0.00104962	-0.00185	0.002	-0.00607	0.002	-0.0113	0.0017	0.0158	0.0064	-0.0062	0.0048	-0.0284	0.03	-0.0051215	0.0142453
rs10818407	0.00791205	0.00124775	-0.00371	0.002	-0.00585	0.002	0.008	0.002	-0.0142	0.0077	-0.0039	0.0055	-0.0602	0.0358	0.0244756	0.0168459
rs10835389	-0.0095145	0.00109639	0.00578	0.002	0.0111	0.002	0.0063	0.0018	-0.0114	0.0067	-0.0071	0.0049	-0.0305	0.0317	0.0124936	0.0149641
rs10853981	-0.0086839	0.00111461	0.00199	0.002	0.00774	0.002	0.0022	0.0017	-0.0299	0.0068	-0.0056	0.0037	0.1855	0.0321	0.0081265	0.0151022
rs10867226	0.00673134	0.00119397	-0.000029	0.002	-0.00321	0.002	-0.0038	0.002	0.0038	0.0072	-0.0106	0.0055	-0.0151	0.0341	0.0180589	0.0161679
rs10886010	-0.0086991	0.0010603	-0.000203	0.002	0.00544	0.002	0.0086	0.0018	-0.0141	0.0065	-0.0119	0.005	-0.0975	0.0308	-0.0009226	0.0145455
rs10887465	0.0127558	0.00118463	0.00132	0.002	-0.000759	0.002	-0.0062	0.0019	0.0257	0.007	-0.0051	0.0054	-0.0358	0.0339	-0.0127191	0.0159504
rs10890995	-0.0092015	0.00114194	-0.000406	0.002	0.00281	0.002	0.0068	0.0019	-0.0088	0.0069	-0.0026	0.0051	0.0465	0.033	0.0116473	0.0155967
rs10896636	0.0093576	0.00110365	0.000909	0.002	0.00189	0.002	-0.0046	0.0018	-0.007	0.0067	-0.0048	0.005	0.2456	0.0319	-0.0164237	0.0149953
rs10916679	0.00714966	0.0011811	-0.000054	0.002	-0.00131	0.002	-0.0031	0.002	0.02	0.0072	-0.0101	0.0062	-0.0737	0.0344	-0.022429	0.016218
rs10954780	-0.0076932	0.00104694	-0.000424	0.002	0.00262	0.002	0.0052	0.0017	-0.0185	0.0064	-0.0018	0.0048	0.0316	0.0301	0.0203227	0.0141972
rs10977124	-0.0071986	0.00122869	0.000396	0.002	0.000526	0.002	-0.0017	0.002	0.0143	0.0075	-0.0018	0.0058	-0.0389	0.0354	-0.0321824	0.0166547
rs10984444	-0.0116381	0.00104677	-0.000847	0.002	0.000572	0.002	0.0062	0.0017	-0.0073	0.006	-0.0002	0.0034	0.066	0.03	0.0104029	0.0142156
rs11081529	0.0122793	0.00116737	0.00548	0.002	0.0066	0.002	-0.0016	0.0019	-0.0016	0.007	-0.0097	0.0054	-0.0497	0.0333	-0.005015	0.0156189
rs11100308	-0.0109405	0.00111717	0.000528	0.002	0.00566	0.002	0.0114	0.0018	-0.0145	0.0068	-0.0003	0.005	-0.0023	0.0322	0.00219498	0.0151138
rs11158200	0.00860686	0.00105036	-0.00314	0.002	-0.0108	0.002	-0.0014	0.0018	0.0225	0.0064	-0.0006	0.0052	0.0717	0.0308	-0.0071143	0.0142788
rs11159067	0.00947897	0.0011147	0.00024	0.002	-0.00286	0.002	-0.0046	0.0018	0.0127	0.0068	-0.0001	0.0051	0.0496	0.032	-0.0140805	0.0151682
rs11172371	-0.0106973	0.00111955	0.00174	0.002	-0.00118	0.002	0.0051	0.0019	0.013	0.0068	-0.0078	0.0053	-0.0835	0.0319	-0.03438	0.0150767
rs11191193	0.0162974	0.00110103	0.00326	0.002	-0.00621	0.002	-0.0022	0.0018	-0.0038	0.0067	-0.0029	0.005	-0.155	0.0318	-0.0012051	0.0149102
rs11196397	0.00897314	0.00117488	0.00304	0.002	-0.0082	0.002	0.0021	0.0019	-0.0025	0.0071	-0.0114	0.0054	0.0061	0.0339	-0.0275255	0.0159348
rs11213482	-0.0079338	0.00143488	-0.00373	0.002	-0.00159	0.002	0.0016	0.0024	-0.0178	0.0088	-0.001	0.0081	0.0425	0.042	0.0118138	0.0187735
rs11218422	0.00879043	0.00105992	-0.00226	0.002	-0.00428	0.002	0.0023	0.0018	-0.002	0.0064	-0.0001	0.0048	0.0208	0.0306	-0.002926	0.0144264
rs11243852	0.0122412	0.00122868	-0.00355	0.002	-0.00175	0.002	-0.0016	0.002	0.0046	0.0076	-0.0014	0.0054	0.0373	0.0352	0.0292196	0.0167076
rs11505217	0.00949123	0.00142334	0.00284	0.002	-0.0062	0.002	-0.0024	0.0024	0.0238	0.0089	-0.0045	0.0066	-0.1892	0.0416	-0.0107852	0.0196929
rs11576565	0.0127578	0.0010474	-0.00134	0.002	-0.0079	0.002	-0.003	0.0017	0.0052	0.0064	-0.0015	0.005	-0.0206	0.0304	0.0124393	0.0142123
rs11614957	0.0120583	0.00105455	-0.00171	0.002	-0.00564	0.002	-0.0033	0.0017	0.0205	0.0065	-0.0018	0.0049	-0.1503	0.0302	-0.0182905	0.0142993
rs11623285	-0.0129564	0.00153993	-0.00363	0.002	0.00286	0.002	-0.0001	0.0026	-0.0225	0.0095	-0.0064	0.0088	0.1	0.045	-0.0431776	0.020582
rs11629584	0.00700523	0.00104737	-0.000641	0.002	0.00131	0.002	-0.0023	0.0017	0.0115	0.0064	-0.0025	0.0049	-0.1199	0.0301	-0.0112433	0.014209
rs11633934	0.00803016	0.00105045	0.00255	0.002	-0.00272	0.002	-0.0002	0.0018	0.0129	0.0065	-0.0034	0.0049	-0.0088	0.0303	0.00096948	0.0143286
rs11643516	-0.0081182	0.00106293	-0.00272	0.002	0.00159	0.002	-0.0044	0.0017	0.0084	0.0065	-0.0016	0.0048	0.0043	0.0305	-0.0206265	0.0144376
rs11690035	-0.019325	0.00199642	-0.00561	0.003	0.00357	0.003	0.0206	0.0034	-0.0148	0.012	-0.0114	0.0095	0.1746	0.0582	3.1456E-05	0.0277941
rs11698330	0.0101121	0.00130545	0.00111	0.002	-0.00104	0.002	-0.0019	0.0022	0.0058	0.008	-0.0018	0.006	-0.0449	0.0376	0.0151619	0.0176525
rs1171150	0.00920477	0.00108394	0.000604	0.002	-0.00206	0.002	0.0005	0.0018	0.0059	0.0066	-0.0064	0.0049	0.0206	0.0312	-0.0135425	0.0147327

rs11714574	-0.0060853	0.00106041	0.000002	0.002	0.00186	0.002	0.0058	0.0017	0.0141	0.0064	-0.0098	0.0048	0.115	0.0305	0.0210499	0.0144049
rs11724690	0.00949049	0.00114895	-0.000987	0.002	-0.00308	0.002	0.0006	0.0019	0.0037	0.0072	-0.0031	0.0055	-0.0382	0.0336	-0.0185336	0.0158379
rs11737459	0.0107297	0.00113734	-0.00153	0.002	0.00271	0.002	-0.0071	0.0019	-0.0033	0.007	-0.0037	0.0053	-0.0217	0.0331	-0.0276813	0.015462
rs11746390	-0.0085993	0.00118084	-0.00436	0.002	0.00151	0.002	-0.0023	0.002	0.0031	0.0072	-0.004	0.0054	-0.1374	0.0342	0.0317045	0.0161134
rs11752914	0.00796861	0.00131926	0.00119	0.002	-0.00368	0.002	-0.0103	0.0022	0.0119	0.0082	-0.0067	0.0061	-0.0016	0.0381	-0.0219166	0.01791
rs11772108	-0.0082009	0.00117049	0.00295	0.002	0.000933	0.002	0.0035	0.0019	-0.0031	0.0071	-0.003	0.0057	0.0178	0.0344	0.00139837	0.0158531
rs11773992	-0.0138016	0.00136722	0.00149	0.002	0.00924	0.002	-0.0016	0.0023	0.0144	0.0086	-0.0008	0.0065	0.023	0.0404	0.00217559	0.0187911
rs11789013	0.0116659	0.00122032	0.000676	0.002	-0.0016	0.002	0.002	0.002	0.0056	0.0074	-0.0008	0.0055	-0.09	0.0353	0.0105393	0.0166615
rs11857810	0.00757805	0.00107176	-0.00137	0.002	-0.00213	0.002	0.0012	0.0017	-0.0093	0.0065	-0.0018	0.0035	0.1349	0.0306	-0.0093741	0.0145535
rs11861256	0.0100396	0.00133168	-0.000371	0.002	-0.00341	0.002	-0.0061	0.0022	-0.0011	0.008	-0.0034	0.0063	0.0625	0.0386	-0.0063159	0.0182636
rs11876620	0.0176524	0.00177071	0.00673	0.003	-0.00167	0.003	0.0147	0.0029	-0.0305	0.0107	-0.0055	0.0082	0.2257	0.0502	0.00928001	0.0236069
rs11897647	0.00919216	0.00135874	0.000473	0.002	-0.00847	0.002	-0.0002	0.0023	-0.0069	0.0083	-0.0009	0.0063	0.0397	0.0402	0.00993948	0.0190511
rs11925699	-0.007664	0.00104884	0.00199	0.002	0.00157	0.002	-0.0049	0.0017	0.0009	0.0064	-0.0023	0.0048	0.0174	0.0302	-0.0289564	0.0142465
rs11996703	0.00608306	0.00109525	0.00306	0.002	-0.000486	0.002	0.0002	0.0018	0.0151	0.0067	-0.0101	0.005	-0.0015	0.0317	0.00878359	0.0149164
rs12055782	-0.0117761	0.00116273	0.00313	0.002	0.00906	0.002	0.0007	0.0019	-0.0134	0.007	-0.0087	0.0054	0.0682	0.0336	-0.0301455	0.0158021
rs12065238	0.0100911	0.0013943	0.000043	0.002	-0.00517	0.002	-0.0078	0.0023	0.0184	0.0086	-0.0107	0.0063	-0.078	0.0403	0.0115077	0.0189718
rs12107377	0.0063775	0.0010471	0.0011	0.002	-0.000866	0.002	0.0004	0.0017	-0.0046	0.0064	-0.0072	0.0049	-0.007	0.0303	-0.0129986	0.0143034
rs12113634	0.00907811	0.00109185	0.00267	0.002	-0.0033	0.002	-0.0006	0.0018	0.0113	0.0067	-0.0024	0.0049	0.0004	0.0316	-0.0182045	0.0148508
rs12144850	0.00728387	0.0012002	0.000107	0.002	-0.00369	0.002	-0.0063	0.002	0.0058	0.0073	-0.0041	0.0055	-0.04	0.0345	-0.0080079	0.0162195
rs12152036	0.00574845	0.00104669	0.00227	0.002	0.000063	0.002	0.0011	0.0017	0.0039	0.0064	-0.0071	0.0052	0.019	0.0301	-0.0199871	0.0142569
rs12155345	0.00770216	0.00111186	-0.000261	0.002	-0.00646	0.002	0.0026	0.0018	-0.014	0.0068	-0.0052	0.005	0.0409	0.0319	-0.0098078	0.0150046
rs12155540	0.0117212	0.00111398	0.00129	0.002	-0.00853	0.002	-0.0106	0.0018	0.0079	0.0068	-0.0035	0.0052	0.0759	0.0322	0.0330859	0.0150774
rs12201073	-0.0070757	0.00107687	0.0055	0.002	0.0118	0.002	0.0039	0.0018	-0.0116	0.0066	-0.002	0.0049	-0.0723	0.031	0.0115174	0.0145765
rs12206040	-0.0071428	0.0012633	0.0024	0.002	0.00297	0.002	0.0013	0.0019	0.0082	0.0071	-0.0043	0.0062	0.0014	0.0342	0.0340924	0.0156793
rs12240387	-0.0059899	0.00108713	-0.00293	0.002	0.00141	0.002	0.007	0.0018	-0.018	0.0067	-0.0058	0.0049	0.0338	0.0312	0.00419293	0.0147047
rs12349467	0.00689202	0.00113173	0.00756	0.002	-0.00102	0.002	-0.004	0.0019	0.0081	0.007	-0.0073	0.0056	0.0237	0.033	0.00881706	0.0155139
rs12435895	-0.0079781	0.00124643	-0.0027	0.002	0.000647	0.002	-0.0044	0.002	0.0006	0.0075	-0.0054	0.0057	0.0319	0.0353	0.0059299	0.0167284
rs12442630	0.0232255	0.00300138	0.00517	0.005	-0.00439	0.004	-0.0157	0.005	0.0784	0.0177	-0.0097	0.015	0.0145	0.0878	-0.0215664	0.0411251
rs12506222	-0.0142283	0.00105369	-0.000149	0.002	0.00516	0.002	0.0072	0.0017	-0.0294	0.0064	-0.0035	0.0048	0.0608	0.0305	0.018086	0.0143914
rs12516485	0.0125058	0.00158808	-0.00135	0.003	-0.0107	0.002	0.003	0.0027	-0.0058	0.0104	-0.0078	0.0096	-0.0604	0.0475	0.0148548	0.0210272
rs12518468	0.00844692	0.00111106	0.000535	0.002	-0.00024	0.002	0.0046	0.0018	-0.008	0.0069	-0.0004	0.0054	-0.0579	0.0324	-0.0097209	0.0151156
rs12538332	0.00668981	0.00117417	-0.00242	0.002	0.00248	0.002	0.0033	0.002	0.0168	0.0072	-0.0066	0.0058	0.0468	0.0343	0.00707554	0.0159044
rs12613500	0.0101295	0.00105772	0.00192	0.002	0.00041	0.002	-0.0072	0.0017	0.0141	0.0065	-0.002	0.0049	-0.0832	0.0306	-0.0186612	0.0143793
rs12637456	-0.0115279	0.00116134	-0.00302	0.002	0.000138	0.002	0.0007	0.0019	-0.0144	0.0071	-0.0045	0.0052	0.0089	0.0338	-0.0177794	0.015829
rs1267488	-0.0149462	0.0013637	0.000663	0.002	0.00483	0.002	-0.0006	0.0023	-0.0063	0.0087	-0.01	0.0071	0.0987	0.0403	0.0118902	0.0184818
rs12692779	-0.0105238	0.00117246	0.000458	0.002	0.00129	0.002	0.0059	0.002	-0.0225	0.0073	-0.0026	0.0054	-0.0446	0.0343	0.00462962	0.0162305
rs12699131	0.0138205	0.00104805	0.00313	0.002	0.00197	0.002	-0.0048	0.0017	0.0141	0.0064	-0.0008	0.0048	-0.1146	0.0301	0.00255808	0.0141682
rs12768641	0.00940253	0.00122886	-0.00426	0.002	-0.00645	0.002	-0.0073	0.0021	0.0049	0.0079	-0.0011	0.006	-0.0003	0.0361	0.00394248	0.0167642
rs12820589	-0.0100402	0.00126998	0.000588	0.002	0.00657	0.002	-0.0022	0.0022	-0.0074	0.0079	-0.0019	0.0068	0.1605	0.037	-0.0021929	0.0172155
rs12893970	-0.0127241	0.00175943	-0.00381	0.003	0.00188	0.003	0.0044	0.0028	-0.0072	0.0108	-0.0067	0.008	0.1634	0.0495	0.0740518	0.0229941
rs1291865	-0.0103137	0.00104703	0.00492	0.002	0.0116	0.002	0.0057	0.0017	-0.0107	0.0064	-0.0015	0.0049	-0.0358	0.0304	0.00430183	0.0142763
rs12992717	-0.0063164	0.00110187	-0.00103	0.002	0.000919	0.002	-0.0007	0.0018	-0.0154	0.0067	-0.0098	0.005	0.0401	0.0318	-0.0040897	0.0150353
rs13010288	0.020104	0.00156844	0.000449	0.003	-0.00793	0.002	-0.0089	0.0025	0.0263	0.009	-0.0013	0.0069	0.0699	0.0444	-0.031009	0.0209166
rs13030077	0.00762923	0.00136638	-0.00137	0.002	-0.000359	0.002	0.0042	0.0022	-0.0082	0.0083	-0.0143	0.0063	-0.0254	0.0389	-0.0040829	0.0181224
rs13061596	-0.019479	0.00253117	0.00314	0.004	0.0061	0.004	0.0013	0.0044	0.0164	0.016	-0.0095	0.0145	-0.1231	0.076	0.11397	0.0351498
rs13065045	-0.0076317	0.00104852	-0.00373	0.002	-0.000044	0.002	0.0036	0.0017	-0.0167	0.0064	-0.0084	0.0048	0.0637	0.0303	-0.0188491	0.014232
rs13076876	-0.0072255	0.00104858	-0.00723	0.002	-0.000525	0.002	-0.0036	0.0017	-0.0073	0.0064	-0.002	0.0048	0.1237	0.0301	-0.0064862	0.0142168
rs13131350	0.0140289	0.00151482	-0.000232	0.003	-0.00256	0.002	-0.0088	0.0025	0.0287	0.0096	-0.0173	0.0071	-0.2272	0.0445	-0.0244774	0.0205966
rs13140041	-0.0063595	0.00105259	0.00647	0.002	-0.00158	0.002	-0.0003	0.0018	-0.0062	0.0064	-0.001	0.005	0.0346	0.0306	0.00905506	0.0142609
rs13212041	0.0112336	0.00130851	0.00547	0.002	-0.000271	0.002	-0.0077	0.0022	0.0153	0.008	-0.0037	0.0062	-0.004	0.0378	0.0283101	0.0176316
rs13213319	-0.0062222	0.00105422	0.0026	0.002	0.00748	0.002	-0.0037	0.0018	0.0051	0.0065	-0.0057	0.005	-0.0601	0.0307	0.012806	0.0144032
rs13238939	-0.0074278	0.0010466	-0.000039	0.002	0.00276	0.002	0.0032	0.0017	-0.0184	0.0064	-0.0032	0.0048	0.0615	0.0303	0.0229307	0.0142195
rs13252156	0.00898821	0.00107076	-0.00463	0.002	-0.0024	0.002	-0.0005	0.0018	0.0028	0.0065	-0.0137	0.0048	-0.1427	0.031	-0.0098213	0.0145899
rs1329125	-0.0124262	0.00111796	0.000719	0.002	0.00272	0.002	0.004	0.0018	-0.0064	0.0068	-0.0055	0.0042	0.0443	0.0323	0.0345357	0.015211
rs133082	-0.0072331	0.00105609	-0.00221	0.002	0.00314	0.002	0.004	0.0017	-0.0112	0.0065	-0.0021	0.0048	0.0901	0.0303	0.00788384	0.0142728
rs1334297	0.0239964	0.00119412	0.00178	0.002	-0.00405	0.002	-0.0106	0.002	0.0293	0.0072	-0.0026	0.0055	0.0082	0.0341	-0.0257744	0.0161113

rs1378893	0.00907707	0.00122904	-0.00456	0.002	-0.00429	0.002	0.0135	0.002	-0.008	0.0075	-0.0027	0.0056	0.0493	0.0352	-0.0130769	0.0166419
rs1396558	-0.0076254	0.00105181	0.00408	0.002	0.00147	0.002	-0.0019	0.0017	0.0042	0.0063	-0.0141	0.0049	0.0046	0.0301	-0.015311	0.0142387
rs1397870	-0.0084448	0.00131838	0.00137	0.002	0.00459	0.002	0.005	0.0022	-0.0051	0.0081	-0.0083	0.006	0.0277	0.0383	0.0205337	0.0180981
rs1402954	0.0146371	0.00177619	-0.0076	0.003	-0.0116	0.003	-0.0007	0.003	0.0175	0.011	-0.0005	0.0084	-0.174	0.052	-0.0376272	0.0246937
rs1452366	-0.0119117	0.00109575	0.00164	0.002	0.00421	0.002	0.0009	0.0018	-0.0116	0.0067	-0.0016	0.005	-0.0217	0.0318	-0.0150201	0.0149244
rs1455350	-0.0160963	0.00104749	-0.000312	0.002	0.00823	0.002	-0.0005	0.0017	-0.0026	0.0064	-0.0085	0.0048	0.0782	0.0303	0.0145024	0.0142868
rs1461224	0.00573636	0.00104774	-0.00201	0.002	-0.00291	0.002	-0.0078	0.0017	0.0031	0.0064	-0.0047	0.0048	0.0461	0.0302	-0.0259781	0.0141916
rs1464018	-0.0136104	0.00128332	-0.00105	0.002	0.000964	0.002	0.0078	0.0023	-0.0076	0.0077	-0.0022	0.0085	0.0348	0.0368	0.0107027	0.017603
rs1464297	-0.0106953	0.00109813	-0.00002	0.002	-0.00135	0.002	0.002	0.0018	-0.0082	0.0066	-0.0044	0.005	0.06	0.0316	0.0273536	0.0149948
rs1470223	0.00989203	0.00127999	0.000096	0.002	-0.00352	0.002	0.0009	0.0021	-0.0007	0.0077	-0.0007	0.0058	0.0221	0.0366	0.031448	0.017313
rs1483148	0.0120892	0.00118026	-0.00208	0.002	0.00352	0.002	0.0103	0.0019	-0.0134	0.0071	-0.0085	0.0053	-0.0996	0.0336	-0.0121394	0.01585
rs1483814	0.00734045	0.00130831	0.00156	0.002	-0.00425	0.002	0.0029	0.0021	-0.003	0.008	-0.0045	0.006	0.12	0.0374	-0.0092359	0.0174627
rs1539414	-0.0107426	0.00127999	-0.000925	0.002	-0.00258	0.002	0.0097	0.0021	-0.008	0.0077	-0.0053	0.0059	-0.0754	0.0371	0.0401314	0.0173314
rs1544	-0.0077654	0.00117698	0.0036	0.002	0.00167	0.002	0.0064	0.002	-0.0095	0.0073	-0.009	0.0056	0.0488	0.0342	-0.009626	0.016051
rs1564860	0.00820625	0.00113655	-0.00364	0.002	-0.00236	0.002	0.0032	0.0019	-0.0033	0.0069	-0.0016	0.0053	-0.034	0.0329	0.005965	0.015481
rs1566779	-0.0097122	0.00107701	0.000952	0.002	0.00247	0.002	0.0058	0.0018	-0.0088	0.0065	-0.008	0.0049	0.0322	0.031	0.00464472	0.0145529
rs1638526	0.00888041	0.00119501	0.00303	0.002	0.0035	0.002	0.0047	0.002	-0.0181	0.0073	-0.0071	0.0055	-0.134	0.034	0.00821222	0.0160658
rs1648404	0.00775717	0.00104801	0.00266	0.002	0.00328	0.002	-0.0039	0.0017	0.0108	0.0064	-0.0002	0.0048	-0.0077	0.0304	0.02119	0.0142239
rs164938	-0.0080638	0.00107104	0.00182	0.002	0.00197	0.002	0.0068	0.0018	-0.0096	0.0065	-0.0051	0.0048	0.0766	0.031	0.0144822	0.0145524
rs1650823	0.00688134	0.00114647	-0.00281	0.002	-0.00751	0.002	-0.0062	0.0019	-0.0203	0.0068	-0.0112	0.0052	-0.0141	0.0327	0.0353092	0.0154496
rs16878083	-0.0071933	0.00124509	-0.00173	0.002	0.000563	0.002	-0.0018	0.0021	-0.0047	0.0075	-0.0074	0.0056	0.0071	0.0363	0.023296	0.017162
rs16903327	-0.0062266	0.00107185	0.00128	0.002	0.000718	0.002	-0.0047	0.0018	0.0012	0.0065	-0.0001	0.0048	-0.0028	0.0308	0.0172607	0.0145279
rs16970633	-0.0111471	0.00143404	-0.00292	0.002	0.00843	0.002	-0.003	0.0024	0.0096	0.0088	-0.014	0.0069	-0.0612	0.0408	-0.0175361	0.0191585
rs16983844	0.0107414	0.00128902	0.000492	0.002	-0.0076	0.002	-0.0062	0.0021	-0.001	0.0082	-0.0004	0.006	-0.0862	0.0375	0.0190295	0.0172453
rs17088142	-0.0068268	0.00104865	-0.00658	0.002	0.00287	0.002	0.0026	0.0018	0.0037	0.0065	-0.0037	0.0049	0.0541	0.0302	-0.0091006	0.0143356
rs17096452	0.0113818	0.00126992	-0.000614	0.002	-0.0126	0.002	-0.0048	0.0021	0.0189	0.0077	-0.0099	0.0058	-0.2686	0.0366	-0.0332919	0.0171861
rs17224289	-0.0131703	0.00161215	-0.00567	0.003	0.00504	0.002	0.0161	0.0027	-0.0257	0.0099	-0.0172	0.0073	-0.1988	0.0469	0.0560986	0.0219115
rs17234990	-0.0083571	0.0011219	0.00225	0.002	0.00317	0.002	-0.0041	0.0017	-0.0094	0.0068	-0.0021	0.0036	-0.0274	0.0323	0.0371077	0.0152265
rs17248751	-0.0137524	0.00127334	-0.00115	0.002	0.00346	0.002	0.0015	0.0021	-0.0025	0.0077	-0.0021	0.0061	0.0721	0.0364	-0.0008623	0.0169707
rs17266097	0.00879034	0.00106911	0.00111	0.002	-0.00605	0.002	-0.0002	0.0018	0.0008	0.0066	-0.0008	0.005	0.0249	0.0312	-0.0018133	0.0145643
rs17378539	0.00892359	0.00139212	-0.00531	0.002	-0.00355	0.002	-0.0035	0.0023	0.009	0.0085	-0.0015	0.0071	0.0234	0.0405	-0.0017237	0.0184977
rs1738050	-0.0110034	0.00107581	0.00477	0.002	0.00773	0.002	-0.0004	0.0018	0.0059	0.0066	-0.001	0.005	-0.0489	0.0311	0.0268287	0.0146092
rs17426562	0.0169736	0.00212862	-0.00472	0.004	-0.00405	0.003	-0.0137	0.0036	0.0181	0.0131	-0.007	0.0107	-0.1053	0.0624	0.00768112	0.0293261
rs17468213	0.0100301	0.00117107	-0.00134	0.002	0.00391	0.002	0.0037	0.0019	-0.0105	0.0071	-0.0018	0.0053	-0.076	0.0336	0.0108209	0.0159444
rs174768	0.0103903	0.00149123	-0.000207	0.002	-0.00339	0.002	-0.0027	0.0024	0.0194	0.0091	-0.0016	0.0069	-0.0968	0.0428	-0.0277239	0.0199176
rs17522122	-0.0118007	0.00104866	0.00264	0.002	0.00549	0.002	0.0159	0.0017	-0.0356	0.0064	-0.0118	0.0037	-0.0987	0.0303	0.0211299	0.0142632
rs17523342	0.00923647	0.00133935	-0.00156	0.002	-0.00701	0.002	0.0098	0.0022	-0.0002	0.0084	-0.0013	0.0061	-0.0147	0.0389	0.010583	0.0183291
rs17563464	-0.0126827	0.00127379	-0.00122	0.002	0.00187	0.002	0.0027	0.0022	-0.0059	0.0081	-0.0041	0.0075	0.1142	0.0385	-0.0238961	0.0173116
rs17565975	-0.0121073	0.00105241	0.000167	0.002	0.00809	0.002	-0.0011	0.0018	0.009	0.0064	-0.0082	0.005	-0.1092	0.0306	-0.0176243	0.0143745
rs1759684	-0.0063362	0.00109791	-0.00359	0.002	-0.00112	0.002	0.0024	0.0018	-0.0025	0.0066	-0.014	0.0049	0.0259	0.0316	-0.0086527	0.0149483
rs17598373	0.00731426	0.00105374	-0.000788	0.002	-0.00366	0.002	0.0018	0.0018	0.0007	0.0063	-0.0026	0.0052	0.043	0.0305	-0.023484	0.014285
rs17669337	-0.0100845	0.00106336	0.000268	0.002	0.00343	0.002	-0.0027	0.0017	0.0089	0.0065	-0.0057	0.005	0.0094	0.0308	-0.0176881	0.0143892
rs17721326	0.00865194	0.00114774	-0.00546	0.002	-0.00409	0.002	-0.0006	0.0019	0.0161	0.007	-0.0019	0.0053	-0.0239	0.0335	-0.0114619	0.015646
rs17743339	0.00892176	0.00121247	-0.001	0.002	-0.00296	0.002	-0.0001	0.002	-0.0057	0.0073	-0.0034	0.0054	0.1741	0.0346	-0.0178549	0.0161511
rs17760841	0.0077451	0.00123869	0.00274	0.002	-0.000089	0.002	-0.0029	0.0021	-0.0095	0.0075	-0.0055	0.0059	0.1024	0.0358	0.00119512	0.0170629
rs17835368	0.00973189	0.00111585	-0.00213	0.002	-0.00339	0.002	-0.0014	0.0018	0	0.007	-0.0034	0.0052	-0.0809	0.0319	-0.0070931	0.0151102
rs1787073	-0.0083803	0.0013166	-0.00417	0.002	0.00162	0.002	-0.0005	0.0021	-0.0134	0.0081	-0.0055	0.0061	-0.0138	0.0374	0.0189361	0.0176087
rs184654	0.016505	0.00143352	0.00351	0.002	-0.00661	0.002	-0.0107	0.0023	-0.0525	0.0087	-0.0005	0.0065	-0.0311	0.0409	0.0231083	0.0193888
rs1865407	-0.0153677	0.00155814	0.00234	0.003	0.00411	0.002	0.0082	0.0026	-0.0128	0.0096	-0.0068	0.0073	0.0258	0.0449	-0.0151929	0.0209296
rs1866823	0.0105762	0.00105109	-0.00358	0.002	-0.00422	0.002	-0.0017	0.0018	0.0089	0.0065	-0.0061	0.0052	-0.0411	0.0307	-0.0285464	0.0144064
rs1871745	0.0101912	0.00128506	0.0012	0.002	-0.00812	0.002	-0.0114	0.0021	0.0234	0.0079	-0.0094	0.0058	0.0765	0.0371	0.0248221	0.0176371
rs1880692	0.00766629	0.00104948	0.00158	0.002	-0.000257	0.002	0.0023	0.0017	-0.0068	0.0065	-0.0061	0.0049	-0.0763	0.0303	0.0195676	0.014274
rs1888765	0.00900915	0.00125516	-0.00405	0.002	0.00147	0.002	-0.0003	0.0021	0.0121	0.0076	-0.0051	0.006	-0.0205	0.0371	0.0250761	0.0172726
rs1934619	-0.0086012	0.00116436	0.00158	0.002	0.00216	0.002	0.0013	0.0019	-0.0058	0.0071	-0.0068	0.0053	-0.0434	0.0332	0.00086266	0.0156364
rs1943107	-0.0141384	0.00156988	-0.00579	0.003	0.00335	0.002	0.001	0.0027	-0.0145	0.0098	-0.005	0.0075	-0.126	0.0459	-0.0352847	0.0215762
rs1949197	-0.0093237	0.00106981	0.00249	0.002	0.00757	0.002	0.0125	0.0018	-0.0075	0.0065	-0.0144	0.0049	-0.0506	0.031	-0.0086415	0.0145712

rs1998846	0.00715149	0.00113154	0.000683	0.002	-0.00402	0.002	-0.0005	0.0019	-0.0009	0.0069	-0.0056	0.0054	0.0074	0.0328	0.00098218	0.0153762
rs1999395	-0.0066302	0.00114423	-0.00514	0.002	-0.000374	0.002	0.0027	0.0019	-0.007	0.007	-0.0025	0.0053	0.0085	0.0331	-0.0089151	0.0156114
rs2017850	0.00949171	0.00109887	-0.00586	0.002	-0.00511	0.002	-0.0054	0.0018	0.0015	0.0067	-0.0029	0.005	0.0275	0.0318	0.00426636	0.0149899
rs2026037	0.015448	0.00143384	-0.00571	0.002	-0.00848	0.002	-0.0052	0.0022	0.0066	0.0086	-0.0097	0.0046	-0.0372	0.0406	-0.0052862	0.0191189
rs2050256	0.0155544	0.0013897	-0.00315	0.002	-0.0119	0.002	-0.0103	0.0022	0.0234	0.0085	-0.0068	0.0046	-0.0322	0.0405	-0.0350006	0.0189354
rs2055940	0.00703177	0.00111919	0.00163	0.002	0.00304	0.002	0.0003	0.0019	0.003	0.0069	-0.0072	0.0054	-0.0936	0.0325	-0.0135859	0.0151568
rs2061485	0.00749119	0.00104696	0.000197	0.002	-0.00317	0.002	-0.0031	0.0017	-0.0017	0.0064	-0.0012	0.0048	-0.0505	0.0301	-0.0007214	0.014226
rs2071387	0.00901585	0.00135584	-0.00108	0.002	-0.00334	0.002	-0.0048	0.0021	0.0281	0.0083	-0.0036	0.0044	0.0031	0.0392	-0.0097753	0.0183554
rs208626	0.00596402	0.00105294	0.0011	0.002	0.00344	0.002	-0.0023	0.0018	0.0033	0.0065	-0.0045	0.0049	-0.0003	0.0306	-2.525E-05	0.0143392
rs2097532	0.00827667	0.00109335	0.00199	0.002	-0.00198	0.002	-0.0059	0.0018	0.0042	0.0067	-0.0164	0.0052	0.0336	0.0316	-0.003799	0.0147798
rs211283	-0.0092236	0.00122534	-0.000503	0.002	0.00174	0.002	0.0004	0.002	0.0032	0.0075	-0.0101	0.0058	0.2003	0.0351	-0.0019533	0.0165503
rs2167763	0.0119122	0.0011298	0.00241	0.002	-0.0013	0.002	-0.0004	0.0019	-0.0012	0.0069	-0.006	0.0058	-0.0322	0.0334	-0.0178211	0.0154399
rs2174752	-0.0086221	0.00105122	0.000074	0.002	0.00615	0.002	-0.0004	0.0017	-0.0045	0.0064	-0.0007	0.0049	0.0503	0.0301	-0.0163459	0.0142611
rs2176263	0.00695891	0.00107226	0.00509	0.002	0.000938	0.002	-0.006	0.0017	-0.0071	0.0065	-0.0021	0.0048	-0.1183	0.0307	0.0212952	0.0144982
rs2177083	-0.0074057	0.0010467	0.00325	0.002	0.0056	0.002	0.0038	0.0017	-0.0193	0.0064	-0.0005	0.0051	-0.0202	0.0303	0.00622673	0.014299
rs2214631	0.00706197	0.00116691	0.000649	0.002	0.00527	0.002	-0.0031	0.0019	0.0032	0.0071	-0.0043	0.0053	-0.0111	0.0341	-0.0235943	0.0160308
rs2237432	-0.007257	0.00121296	0.000439	0.002	0.00381	0.002	0.0033	0.002	0.0029	0.0075	-0.0001	0.0055	-0.104	0.0351	-0.0088472	0.0164262
rs2239736	0.00855693	0.00104714	0.00254	0.002	-0.00131	0.002	-0.0062	0.0017	0.0046	0.0065	-0.0044	0.0048	0.0229	0.0303	-0.0162981	0.0142168
rs2241722	0.00757964	0.00112516	0.00106	0.002	-0.0023	0.002	0.0102	0.0019	-0.038	0.0069	-0.0157	0.0068	0.0404	0.0331	0.00408914	0.0151372
rs225285	-0.0087602	0.00117724	0.00206	0.002	0.00493	0.002	0.0022	0.002	0.0099	0.0072	-0.0016	0.0054	0.0744	0.0339	0.00270249	0.0161823
rs2275154	0.015584	0.00111349	-0.00298	0.002	0.000766	0.002	-0.0053	0.0018	-0.0007	0.0069	-0.0038	0.005	-0.1468	0.0321	-0.0004689	0.015171
rs2283250	-0.0077742	0.00104702	-0.00855	0.002	0.000536	0.002	0.0051	0.0017	-0.0032	0.0065	-0.0064	0.0048	0.0355	0.0302	0.00790761	0.0142778
rs2289769	-0.008382	0.0015098	-0.00725	0.003	-0.00337	0.002	0.0063	0.0025	-0.0113	0.0092	-0.0112	0.0071	-0.027	0.0436	-0.0003883	0.0205139
rs2297293	0.00791431	0.00112834	0.00643	0.002	-0.000511	0.002	-0.0009	0.0019	-0.0127	0.0069	-0.0055	0.0053	-0.064	0.0328	0.0171213	0.0154351
rs2303083	0.0133282	0.00133695	0.00249	0.002	-0.00906	0.002	-0.0145	0.0022	0.0231	0.0082	-0.0039	0.0061	-0.3278	0.0383	-0.0117695	0.0179606
rs2303907	0.00852176	0.0010477	-0.00115	0.002	-0.00353	0.002	0.0006	0.0017	0.0059	0.0064	0	0.0048	-0.1546	0.03	0.00099711	0.0141789
rs2303929	0.0093103	0.0012389	0.00173	0.002	0.00223	0.002	0.0069	0.002	-0.0151	0.0074	-0.0098	0.0056	-0.0664	0.0359	-0.0264303	0.0169162
rs2304282	0.011001	0.00106252	-0.00579	0.002	-0.0023	0.002	0.0016	0.0018	0.0031	0.0066	-0.0048	0.0048	-0.1241	0.0308	0.0238378	0.0144604
rs232464	0.00918058	0.00125785	0.000947	0.002	0.00247	0.002	-0.0011	0.002	0.0229	0.0076	-0.0013	0.0056	0.0001	0.0356	-0.0104885	0.0169198
rs234644	-0.0064112	0.00104691	0.0016	0.002	0.000002	0.002	0.0035	0.0017	-0.0037	0.0065	-0.0079	0.0048	0.0999	0.0301	0.0254104	0.014256
rs2371001	-0.0092875	0.00104675	-0.00612	0.002	-0.00235	0.002	0.0042	0.0017	0.0022	0.0063	-0.0118	0.0048	-0.0082	0.0303	0.0189921	0.0142085
rs23766	-0.0109088	0.00124759	0.00385	0.002	0.00604	0.002	0.0043	0.0019	-0.0114	0.0076	-0.0145	0.004	0.0426	0.0357	0.00273799	0.0168593
rs2467974	-0.0069162	0.00110818	0.00382	0.002	0.00737	0.002	-0.0023	0.0018	0.0029	0.0067	-0.0029	0.0052	-0.0057	0.0317	0.0167504	0.0149833
rs2498018	-0.0089306	0.0013306	-0.00465	0.002	0.0025	0.002	0.0038	0.0022	-0.0168	0.0082	-0.0092	0.006	0.0464	0.0381	0.0141097	0.0179807
rs2561477	0.00990309	0.00112459	0.00329	0.002	-0.00216	0.002	0.0038	0.0018	-0.0303	0.0068	-0.0026	0.005	-0.0068	0.0324	-0.0301767	0.0151885
rs2584240	-0.0113465	0.00168162	-0.00602	0.003	0.00374	0.003	-0.002	0.0028	0.0009	0.0104	-0.0004	0.0079	0.0841	0.049	0.00176974	0.0232002
rs2607505	-0.0084581	0.00112805	-0.00459	0.002	-0.00247	0.002	0.0005	0.0019	-0.0147	0.0069	-0.0042	0.0053	0.01	0.0324	-0.0274713	0.0152918
rs2614463	-0.0099034	0.00106418	0.00701	0.002	0.008	0.002	0.0026	0.0018	-0.0104	0.0064	-0.0028	0.0051	0.0829	0.031	-0.0277877	0.0144161
rs2631535	0.0122965	0.00111375	0.000872	0.002	-0.00325	0.002	0.0035	0.0019	0.0113	0.0068	-0.0041	0.0053	-0.0975	0.032	-0.0107214	0.0152508
rs2632667	-0.0061806	0.00104909	0.00126	0.002	0.0027	0.002	0.0011	0.0017	-0.0039	0.0062	-0.0018	0.005	0.0226	0.0305	-0.020025	0.0142779
rs2653344	-0.0085787	0.00130424	-0.00411	0.002	0.00212	0.002	0.0091	0.0021	0.0102	0.0079	-0.0038	0.006	0.1395	0.0372	-0.0254027	0.0173506
rs2668196	-0.011857	0.00132351	-0.00173	0.002	-0.00198	0.002	0.0055	0.0022	-0.0176	0.0082	-0.0062	0.0061	0.0672	0.0383	0.00304455	0.0180824
rs2706762	0.00821664	0.00148043	0.00646	0.002	0.00216	0.002	-0.0058	0.0024	0.0225	0.0092	-0.0129	0.0072	-0.1449	0.0429	-0.0205482	0.0198675
rs27222	-0.0141081	0.00109234	0.00363	0.002	0.00384	0.002	-0.0006	0.0018	-0.0103	0.0067	-0.008	0.005	0.0335	0.0314	-0.0003497	0.0147149
rs2764684	0.0150109	0.00138678	0.00561	0.002	-0.0021	0.002	0.0045	0.0023	0.0112	0.0085	-0.0038	0.0064	-0.0055	0.04	-0.0047419	0.0186185
rs2848780	-0.00808	0.00117628	0.00278	0.002	0.00212	0.002	-0.0034	0.002	-0.0035	0.0072	-0.0034	0.0055	-0.0383	0.0338	-0.0097109	0.0159469
rs2852334	0.00654837	0.0010558	0.00357	0.002	-0.00174	0.002	-0.0034	0.0017	-0.0021	0.0065	-0.0022	0.005	0.0518	0.0303	-0.0093294	0.0143442
rs2867932	0.00714813	0.00107259	0.00536	0.002	-0.000301	0.002	-0.0055	0.0018	-0.0016	0.0065	-0.0064	0.0048	-0.0003	0.0306	0.0133511	0.0144953
rs2884364	-0.0159498	0.00107703	0.00522	0.002	0.00772	0.002	0.0014	0.0017	-0.0112	0.0066	-0.0024	0.0035	0.0725	0.0309	0.0196686	0.0146339
rs2886405	0.00834816	0.00109672	-0.000085	0.002	-0.00369	0.002	0.0008	0.0018	0.0032	0.0073	0	0.0049	0.0213	0.0316	0.00083342	0.0148732
rs2901785	0.0141211	0.0010521	-0.00816	0.002	-0.00864	0.002	0.0022	0.0017	0.0015	0.0064	-0.0045	0.0048	-0.0312	0.0302	0.0125069	0.0142639
rs2935969	0.00798133	0.00122061	0.00281	0.002	0.00084	0.002	-0.0065	0.002	0.0214	0.0074	-0.0036	0.0059	0.0564	0.0351	0.00954034	0.0166261
rs2939261	0.0115023	0.00128038	-0.0035	0.002	-0.00226	0.002	0.0055	0.0021	0.003	0.0077	-0.0035	0.0058	-0.0477	0.0368	0.00040413	0.0171999
rs2941318	0.00821406	0.00105724	-0.000514	0.002	0.00222	0.002	-0.0088	0.0017	0.0065	0.0064	-0.0137	0.0049	-0.0095	0.0304	-0.0388482	0.0143832
rs294599	-0.0087916	0.00156956	0.00114	0.003	0.00494	0.002	0.0019	0.0026	0.0106	0.0095	-0.0077	0.0076	0.0171	0.0457	-0.0043075	0.0215536
rs2964252	0.010242	0.00113042	0.00647	0.002	-0.00111	0.002	-0.0015	0.0019	0.0088	0.0068	-0.005	0.0052	0.0476	0.0325	0.0160567	0.0152864

rs2980813	0.00771047	0.00104885	-0.0012	0.002	-0.00131	0.002	-0.0006	0.0017	-0.0062	0.0064	-0.0019	0.0049	-0.1017	0.0306	-0.0341093	0.0143073
rs3020410	-0.0106402	0.00152846	-0.00383	0.002	0.000716	0.002	-0.0016	0.0024	-0.0004	0.0093	-0.0047	0.0053	0.0065	0.0439	0.00442788	0.0201916
rs3121984	0.00956813	0.00113138	-0.00003	0.002	-0.00705	0.002	-0.0081	0.0019	0.0117	0.0069	-0.003	0.0052	0.1316	0.0328	0.0242917	0.015508
rs322627	-0.0100006	0.00107939	0.000662	0.002	0.00604	0.002	-0.0008	0.0018	0.0074	0.0066	-0.0058	0.0049	0.0117	0.0309	-0.0126195	0.0146089
rs33002	-0.0066288	0.00105311	-0.00201	0.002	-0.00112	0.002	-0.0003	0.0017	-0.0012	0.0064	-0.0003	0.0048	-0.0191	0.0304	-2.625E-05	0.014301
rs34316	0.0162889	0.00106395	0.00412	0.002	-0.00565	0.002	0.0078	0.0017	0.0046	0.0067	-0.003	0.0049	0.0868	0.0306	0.0161348	0.0144326
rs3731507	0.0275792	0.00258907	0.0015	0.004	-0.00734	0.003	-0.008	0.0041	-0.0245	0.0156	-0.0134	0.0108	-0.0988	0.0751	0.00739656	0.0335093
rs3751667	-0.0088902	0.00124898	0.00294	0.002	0.00234	0.002	0.0026	0.0021	-0.0231	0.0077	-0.0031	0.0061	-0.0644	0.0364	0.00705153	0.0169125
rs3768992	0.00659983	0.00104695	0.000441	0.002	-0.00159	0.002	-0.0066	0.0017	0.0208	0.0064	-0.0074	0.005	-0.0424	0.0303	-0.0153872	0.0142788
rs3783006	0.0130873	0.00105247	0.00499	0.002	-0.0024	0.002	-0.0096	0.0017	0.0213	0.0065	-0.0048	0.005	-0.0689	0.0304	0.0143187	0.0142437
rs3811038	0.00950087	0.00116764	-0.0104	0.002	-0.00951	0.002	-0.004	0.0019	0.0122	0.0071	-0.0016	0.0054	-0.1087	0.0338	-0.0133807	0.0159644
rs386207	-0.011523	0.00162434	-0.00782	0.003	0.00777	0.002	0.0003	0.0027	0.0046	0.0102	-0.0051	0.0077	0.0073	0.0472	-0.0150595	0.0221909
rs3863241	-0.0085901	0.0010502	-0.00218	0.002	0.00514	0.002	0.0087	0.0017	-0.0104	0.0064	-0.0041	0.0048	0.0034	0.0304	0.00957637	0.0142105
rs3936100	0.00822656	0.00108762	-0.00188	0.002	-0.00255	0.002	-0.0003	0.0018	0.0034	0.0067	-0.0058	0.005	0.0518	0.0312	0.0148481	0.0147256
rs405509	0.00654834	0.00104859	-0.000152	0.002	-0.00228	0.002	-0.01	0.0017	0.0089	0.0065	-0.0174	0.0038	0.0792	0.0306	0.0043506	0.0141874
rs406413	0.01271	0.00127752	0.00218	0.002	0.00683	0.002	-0.0114	0.0021	0.0225	0.0079	-0.0079	0.0058	-0.1484	0.0367	-0.0219407	0.0173803
rs4127499	0.0109595	0.00110304	0.000407	0.002	-0.00299	0.002	-0.0033	0.0018	0.0145	0.0067	-0.0103	0.005	0.0654	0.0323	0.0312606	0.0148047
rs4142529	-0.0126233	0.00162883	-0.003	0.003	-0.00499	0.003	0.0183	0.0026	-0.0156	0.0102	-0.0133	0.0073	0.1701	0.0461	0.0257254	0.0218555
rs41741	0.00737479	0.00111229	-0.00102	0.002	-0.0092	0.002	-0.0063	0.0018	0.0032	0.0068	-0.0167	0.005	-0.1584	0.032	0.00235463	0.0151497
rs4254203	-0.0150556	0.0023665	0.00281	0.004	0.00296	0.004	0.0064	0.004	-0.0495	0.0146	-0.0134	0.0117	-0.0927	0.0683	0.00160177	0.0322142
rs4358081	-0.009704	0.00104852	-0.00136	0.002	0.00429	0.002	-0.0097	0.0017	-0.0049	0.0064	-0.0066	0.0047	-0.0262	0.0302	0.00099769	0.0142002
rs4378243	0.0162224	0.00142441	-0.00472	0.002	-0.00313	0.002	-0.0141	0.0023	0.0156	0.0079	-0.007	0.0063	0.0891	0.0406	-0.0209331	0.0191228
rs4391653	-0.0081619	0.00118982	-0.000851	0.002	0.00419	0.002	0.0077	0.0019	-0.021	0.0072	-0.01	0.0055	0.0569	0.0342	0.0211566	0.0160573
rs4409028	0.0059634	0.00109308	-0.000775	0.002	-0.00571	0.002	-0.0008	0.0018	0.0079	0.0066	-0.0045	0.0054	-0.0272	0.0317	0.0349169	0.0148671
rs4460271	0.00776893	0.00111514	0.000792	0.002	-0.00462	0.002	-0.009	0.0018	0.0082	0.0068	-0.0006	0.005	0.0593	0.0322	-0.0189735	0.0151
rs4483480	-0.0076832	0.00105843	-0.000821	0.002	0.00106	0.002	0.0022	0.0017	-0.0046	0.0065	-0.0019	0.0048	-0.0148	0.0304	-0.0032071	0.0143511
rs4545434	0.00971248	0.00134689	-0.0004	0.002	-0.00489	0.002	-0.0052	0.0022	-0.0028	0.0083	-0.0164	0.0061	0.0132	0.0392	0.0436995	0.0184582
rs4553692	0.0112065	0.00108294	0.0078	0.002	0.00114	0.002	0.0023	0.0018	0.0078	0.0066	-0.0034	0.0049	-0.0754	0.031	-0.0019007	0.0147281
rs4583487	0.0229768	0.00106986	-0.00279	0.002	-0.00772	0.002	-0.0143	0.0017	0.0233	0.0065	-0.0086	0.0049	0.0672	0.0307	-0.0250268	0.014475
rs4654441	0.00724157	0.00105865	0.00632	0.002	0.00215	0.002	-0.0065	0.0017	0.02	0.0065	-0.0034	0.0048	0.0203	0.0306	-0.0126552	0.0143686
rs4662573	0.00692923	0.00104926	0.00176	0.002	-0.000194	0.002	-0.001	0.0017	0.006	0.0064	-0.0079	0.0048	-0.0547	0.0303	-0.0016187	0.014263
rs4668897	0.00787334	0.00121549	-0.000522	0.002	-0.00427	0.002	-0.0066	0.0019	0.0028	0.0073	-0.0044	0.0041	-0.0385	0.0354	0.00150782	0.0164217
rs4677087	-0.0060622	0.0010886	-0.00382	0.002	0.00216	0.002	-0.0005	0.0018	-0.0005	0.0066	-0.0005	0.0053	0.0006	0.0314	-0.0050165	0.0147265
rs4678463	-0.0095026	0.00120552	-0.00072	0.002	0.0021	0.002	0.0005	0.002	0.005	0.0073	-0.005	0.0055	0.1453	0.0346	-0.001591	0.0162213
rs4680176	0.00688731	0.00107823	-0.00147	0.002	-0.0022	0.002	-0.0086	0.0018	0.0133	0.0066	-0.0006	0.0049	-0.0492	0.0311	-0.031891	0.0146919
rs4685448	-0.0067876	0.00105405	0.000232	0.002	0.00173	0.002	-0.0029	0.0018	-0.0016	0.0065	-0.0062	0.0048	0.0759	0.0305	0.00552503	0.0143868
rs4697062	0.00775689	0.00105505	0.0028	0.002	-0.00598	0.002	0.0006	0.0017	0.0101	0.0065	-0.007	0.0049	-0.0655	0.0306	-0.0039306	0.0142983
rs4706938	0.00608041	0.00105928	-0.000106	0.002	-0.000331	0.002	-0.0023	0.0017	0.009	0.0066	-0.0083	0.0049	-0.0705	0.0305	0.00109235	0.0143111
rs4709995	-0.0073586	0.00107005	-0.00156	0.002	0.00202	0.002	-0.0052	0.0018	-0.0035	0.0066	-0.003	0.0049	0.0528	0.0309	0.0281281	0.0146082
rs4724085	-0.0087458	0.00112782	-0.00307	0.002	0.00467	0.002	-0.007	0.0019	-0.007	0.0069	-0.0002	0.0053	0.0474	0.0327	-0.0576974	0.0154417
rs4725065	-0.0121832	0.00104673	0.0039	0.002	0.00259	0.002	0.0018	0.0017	-0.0101	0.0064	-0.0038	0.0048	0.0462	0.0301	0.00105728	0.0142214
rs4728354	0.00986285	0.00105237	-0.00663	0.002	-0.00605	0.002	0.0042	0.0017	0.0037	0.0064	-0.0085	0.0049	-0.0744	0.0305	0.0250683	0.0142895
rs473900	-0.0077783	0.00121713	-0.00268	0.002	-0.00333	0.002	-0.0023	0.002	-0.0011	0.0074	-0.0014	0.0054	0.0295	0.035	0.0150311	0.0165855
rs4739408	0.00617606	0.00104971	-0.000752	0.002	-0.00116	0.002	0.0012	0.0017	-0.0145	0.0064	-0.0103	0.005	-0.002	0.0306	0.0239613	0.0142338
rs4741600	0.0158013	0.00110472	-0.00118	0.002	-0.000417	0.002	0.001	0.0018	-0.003	0.0067	-0.0029	0.005	-0.0005	0.0316	-0.0081376	0.0150056
rs4766975	-0.0087139	0.00107225	0.00457	0.002	0.00832	0.002	0.0036	0.0018	-0.0273	0.0066	-0.0003	0.0049	-0.014	0.0306	-0.0009457	0.0144975
rs4772268	0.0099177	0.00110983	-0.00142	0.002	-0.0037	0.002	-0.0015	0.0019	0.0123	0.0069	-0.0032	0.0053	-0.1	0.0322	-0.0246456	0.0152894
rs4785187	-0.0116212	0.00125747	-0.00268	0.002	0.0119	0.002	0.004	0.002	0.0064	0.0077	-0.0014	0.0042	-0.1334	0.0362	0.00643993	0.0170665
rs4793109	0.00673436	0.00105467	-0.00117	0.002	-0.00448	0.002	-0.0002	0.0017	-0.0176	0.0064	-0.0072	0.0048	0.1031	0.0303	0.0212183	0.0143377
rs4805761	-0.0139463	0.00144818	-0.00629	0.002	0.00536	0.002	0.002	0.0024	-0.0181	0.009	-0.0062	0.0065	0.0651	0.0411	-0.0076577	0.0195268
rs4818226	0.0112177	0.00112168	-0.00123	0.002	-0.00537	0.002	-0.012	0.0019	0.011	0.0068	-0.0112	0.0051	0.0173	0.0322	-0.0202566	0.0152831
rs483673	-0.0071194	0.00121613	0.00321	0.002	0.00316	0.002	-0.0001	0.002	-0.0095	0.0074	-0.001	0.0058	0.0672	0.035	-0.0086175	0.0163677
rs4846724	0.00766902	0.00104874	0.000003	0.002	-0.00224	0.002	-0.007	0.0017	0.0043	0.0064	-0.0143	0.0048	-0.0232	0.0302	-0.0054495	0.014279
rs4848732	-0.0071558	0.00105266	-0.000172	0.002	-0.00234	0.002	-0.003	0.0017	-0.0056	0.0064	-0.0082	0.0048	0.0869	0.0302	0.0133844	0.0142557
rs4855037	-0.0061039	0.00105994	0.00213	0.002	0.00479	0.002	0.0046	0.0018	-0.0062	0.0065	-0.0012	0.0058	0.0275	0.031	0.00883813	0.0143147
rs4864881	-0.0069829	0.0010734	0.000106	0.002	0.00287	0.002	-0.0027	0.0018	-0.0063	0.0065	-0.0027	0.0051	0.0141	0.0309	0.0173359	0.0144631

rs4872449	0.00953027	0.00105693	0.00421	0.002	-0.00347	0.002	-0.0024	0.0017	-0.0042	0.0064	-0.0044	0.0049	-0.002	0.0304	0.00590692	0.0143413
rs4876775	-0.0112554	0.0011264	-0.000392	0.002	0.0038	0.002	-0.0073	0.0018	-0.0169	0.0068	-0.0026	0.005	0.0185	0.0322	-0.0100834	0.0152266
rs4879832	-0.0063744	0.00107971	-0.00241	0.002	-0.000586	0.002	0.0002	0.0018	-0.0023	0.0066	-0.0108	0.005	-0.04	0.0312	0.00283375	0.0145774
rs4899200	-0.0071982	0.00117453	-0.00414	0.002	0.000187	0.002	0.0067	0.002	-0.0248	0.0072	-0.0024	0.0053	-0.0565	0.0339	0.0186521	0.0160481
rs4909854	-0.0063633	0.001077	-0.00161	0.002	0.00416	0.002	0.0072	0.0018	0.0054	0.0066	-0.0062	0.005	-0.0206	0.0311	0.0006923	0.0146193
rs4911257	0.00979146	0.00107435	-0.00579	0.002	-0.00907	0.002	0.0015	0.0018	0.0116	0.0066	-0.0096	0.0049	-0.1308	0.0308	0.00787815	0.0145495
rs4925093	-0.0073556	0.00104719	-0.00592	0.002	0.00128	0.002	-0.0019	0.0017	-0.0261	0.0064	-0.0079	0.0048	0.0811	0.0301	0.0192546	0.014216
rs4943074	0.00810865	0.00105155	0.00225	0.002	-0.0028	0.002	-0.0015	0.0017	0.001	0.0065	-0.0076	0.0048	-0.114	0.0303	0.0130274	0.014366
rs4969315	-0.0067736	0.00106522	0.000916	0.002	0.00157	0.002	0.0039	0.0018	-0.0014	0.0065	-0.0041	0.0049	0.0238	0.0306	0.0220285	0.0144762
rs533123	-0.0079126	0.00132548	-0.00479	0.002	-0.000466	0.002	-0.0025	0.0022	0.0189	0.0082	-0.0003	0.0065	-0.0485	0.0384	0.0216055	0.0178826
rs538249	-0.0067376	0.00106059	-0.00137	0.002	-0.000949	0.002	0.001	0.0017	-0.0238	0.0065	-0.0003	0.0049	-0.042	0.0308	0.00612623	0.0143728
rs561655	-0.0089131	0.00110096	-0.00262	0.002	0.000651	0.002	-0.0006	0.0018	-0.0023	0.0067	-0.0012	0.005	-0.0594	0.0316	0.00704146	0.0149
rs565960	0.0087496	0.00115594	-0.00155	0.002	-0.00442	0.002	-0.0026	0.002	-0.0036	0.007	-0.0073	0.0058	0.0279	0.0339	0.00525011	0.0160191
rs5765717	-0.0072398	0.00104688	0.00106	0.002	0.0027	0.002	-0.0015	0.0017	0.0009	0.0064	-0.0015	0.0048	-0.0313	0.03	0.00201209	0.0142266
rs6013574	0.0148619	0.00214951	-0.00592	0.004	-0.00997	0.003	-0.0022	0.0036	-0.0267	0.013	-0.0181	0.0102	-0.1297	0.0622	-0.0024792	0.0294158
rs6020560	-0.0095654	0.00104786	0.00237	0.002	0.00625	0.002	0.0033	0.0017	0.0024	0.0064	-0.0006	0.0048	-0.0137	0.03	-0.0091093	0.0142381
rs6028103	0.010416	0.00138147	0.00603	0.002	-0.000055	0.002	0.0007	0.0023	-0.0098	0.0087	-0.0094	0.0063	0.0075	0.0399	-0.0268273	0.0191882
rs6035413	-0.0088227	0.0010982	0.0023	0.002	0.00784	0.002	0.001	0.0018	-0.0118	0.0067	-0.004	0.005	-0.002	0.0314	0.0241967	0.0149255
rs6093705	0.00953909	0.00112218	0.000577	0.002	0.000407	0.002	-0.0007	0.0018	-0.0002	0.0068	-0.0034	0.0051	-0.1175	0.032	-0.0041931	0.0150901
rs6425839	0.00790107	0.00104953	-0.000818	0.002	-0.00419	0.002	-0.0088	0.0017	0.0179	0.0065	-0.006	0.0049	0.0707	0.0308	-0.0079669	0.0143278
rs6428152	-0.0080543	0.00116859	0.000726	0.002	0.000548	0.002	0.0084	0.0019	-0.0087	0.0071	-0.0034	0.0054	0.1495	0.0337	0.0083742	0.0158772
rs6457796	0.0104006	0.0011664	-0.00183	0.002	-0.00693	0.002	-0.0254	0.0018	-0.0021	0.0072	-0.0264	0.0038	-0.0345	0.034	-0.0489213	0.0159843
rs6465603	0.0110417	0.00135933	-0.00496	0.002	-0.00925	0.002	-0.0034	0.0023	0.0054	0.0084	-0.004	0.0063	-0.0157	0.0392	-0.0288278	0.0183829
rs6466819	-0.0103106	0.00108737	-0.00357	0.002	0.000824	0.002	0.0098	0.0018	-0.008	0.0068	-0.0062	0.0049	0.0143	0.0314	0.00486155	0.0148069
rs6534293	0.00699756	0.00105465	0.00505	0.002	0.00416	0.002	-0.0025	0.0017	0.0087	0.0064	-0.0003	0.0049	-0.0647	0.0304	0.00375455	0.0143498
rs6534338	0.0104741	0.00114042	-0.00381	0.002	-0.00588	0.002	-0.0042	0.0018	-0.0037	0.0069	-0.0054	0.0037	0.022	0.033	-0.0076925	0.0156275
rs6534704	-0.0149996	0.00188823	0.00451	0.003	0.00314	0.003	0.0009	0.003	0.0219	0.0113	-0.0098	0.0085	-0.0147	0.0534	0.00297878	0.0245862
rs6539284	-0.0072088	0.00106643	0.00109	0.002	0.00312	0.002	-0.0032	0.0017	-0.0049	0.0065	-0.0082	0.0051	0.2271	0.0305	0.00726711	0.0143457
rs6547396	0.00772378	0.00106686	0.00109	0.002	-0.000553	0.002	-0.0006	0.0017	0.0151	0.0065	-0.002	0.0048	-0.0711	0.0305	0.00852723	0.0144522
rs654880	-0.0084191	0.00126007	0.000707	0.002	0.000094	0.002	-0.0052	0.0021	-0.0143	0.0077	-0.0017	0.0062	0.037	0.0374	-0.0011565	0.0170788
rs6563363	-0.0092103	0.00108202	0.00109	0.002	0.00245	0.002	0.001	0.0018	0.0061	0.0066	-0.002	0.0049	-0.0077	0.0312	0.00864208	0.0147632
rs6580068	-0.0093054	0.0012658	0.00132	0.002	0.00265	0.002	-0.0011	0.0021	-0.0127	0.0077	-0.0042	0.0057	0.0334	0.0367	-0.025361	0.0172691
rs6587843	0.00741178	0.00104709	0.00236	0.002	-0.000278	0.002	-0.0027	0.0017	0.0046	0.0064	-0.0039	0.0048	-0.0507	0.0301	0.0115564	0.0142014
rs6669004	-0.007226	0.00117239	0.00128	0.002	0.00055	0.002	-0.002	0.0019	0.0106	0.0072	-0.0002	0.0053	-0.0063	0.0335	-0.0093947	0.0158864
rs6689362	-0.0101693	0.00113719	0.00218	0.002	0.00769	0.002	-0.0038	0.0019	-0.0072	0.0069	-0.0048	0.0053	-0.0176	0.0327	0.00270632	0.015356
rs6693597	0.00896291	0.00153776	0.00328	0.003	0.000852	0.002	-0.0003	0.0026	0.0135	0.0095	-0.0004	0.0087	0.1165	0.0455	0.00651941	0.0208449
rs6704241	0.00859319	0.00110715	0.000305	0.002	-0.00437	0.002	-0.0035	0.0018	0.0075	0.0068	-0.0022	0.0053	0.0716	0.0318	0.0125095	0.0148607
rs6704768	-0.0153293	0.00105609	-0.00206	0.002	-0.00371	0.002	-0.003	0.0017	-0.0066	0.0064	-0.0036	0.0048	-0.0207	0.0304	0.00846141	0.0143358
rs6710557	-0.0067384	0.00104906	-0.00296	0.002	0.000396	0.002	0.0026	0.0016	-0.0096	0.0064	-0.0005	0.0034	0.0061	0.0305	0.00149412	0.0142752
rs6715321	-0.0097874	0.0010578	-0.00609	0.002	0.00613	0.002	0.0041	0.0017	0.0048	0.0065	-0.0075	0.0048	0.0038	0.0305	0.0118701	0.0143514
rs6717169	0.0079815	0.00132821	-0.000309	0.002	-0.00359	0.002	-0.0014	0.0022	-0.0032	0.0081	-0.0124	0.0063	-0.0008	0.0382	0.0100793	0.018026
rs6721505	0.00736174	0.00111528	0.00125	0.002	0.00292	0.002	-0.0067	0.0019	0.012	0.0068	-0.0067	0.0052	0.0607	0.0324	-0.0025561	0.0152539
rs6731373	-0.0087233	0.00110319	0.00128	0.002	-0.00117	0.002	0.0028	0.0018	0.0099	0.0068	-0.0002	0.0059	0.1913	0.0326	-0.0195306	0.0147959
rs6747129	-0.0156817	0.001728	0.005	0.003	0.0118	0.003	-0.0073	0.0028	-0.0111	0.0106	-0.0056	0.0078	-0.1826	0.0494	-0.0388414	0.0235064
rs6755567	0.00689942	0.00121642	-0.00646	0.002	-0.00609	0.002	0.0032	0.002	0.0039	0.0074	-0.0038	0.0058	-0.0973	0.0352	-0.0200958	0.0164635
rs6757087	-0.007796	0.00105813	-0.00207	0.002	0.00186	0.002	0.0037	0.0018	-0.0031	0.0065	-0.0019	0.0051	-0.0429	0.031	0.0206038	0.0144733
rs6782698	0.0138302	0.00121445	-0.00387	0.002	-0.00786	0.002	0.001	0.002	0.0015	0.0073	-0.0124	0.0057	-0.0448	0.0347	0.00414666	0.0163738
rs6852084	-0.0104754	0.0010765	0.00283	0.002	0.0145	0.002	0.0062	0.0018	0.0046	0.0065	-0.0126	0.0051	-0.0817	0.031	-0.0096633	0.01463
rs6864049	0.0083344	0.00104846	0.000626	0.002	-0.0041	0.002	-0.0125	0.0017	0.004	0.0064	-0.0074	0.0034	-0.028	0.0304	-0.0231207	0.0143862
rs6898748	-0.0066089	0.00105383	-0.00411	0.002	-0.00215	0.002	0.0009	0.0017	0.0056	0.0064	-0.001	0.0048	0.035	0.0304	-0.0061314	0.014303
rs6926377	0.0080678	0.00113443	0.000326	0.002	-0.00327	0.002	0.001	0.0019	0.0061	0.007	-0.0048	0.0052	-0.0615	0.0332	-0.0270842	0.0155721
rs7009856	0.00671103	0.00107384	-0.000414	0.002	0.00037	0.002	0.0007	0.0018	0.0047	0.0066	-0.0021	0.0051	-0.0075	0.0313	-0.0110675	0.0146309
rs7045411	0.0192529	0.00146319	0.0045	0.002	-0.00495	0.002	-0.0048	0.0024	0.0279	0.009	-0.0003	0.0065	-0.0645	0.0424	-0.0089389	0.0200702
rs705240	-0.0130133	0.0013475	-0.000359	0.002	0.00311	0.002	0.0059	0.0022	-0.0072	0.0083	-0.0018	0.0065	-0.0037	0.0388	-0.0112727	0.0182881
rs707084	0.00658755	0.00105004	0.000001	0.002	-0.00241	0.002	0	0.0017	-0.0111	0.0065	-0.0002	0.0048	-0.07	0.0303	0.00984108	0.0143036
rs707247	0.00632639	0.00104846	-0.00115	0.002	0.00108	0.002	0.005	0.0017	-0.0046	0.0064	-0.0012	0.0052	-0.0776	0.0303	-0.0246381	0.0142756

rs708912	0.0103668	0.00128942	0.000773	0.002	-0.00116	0.002	0.0104	0.0021	-0.0131	0.0079	-0.0117	0.0059	-0.0343	0.0371	0.00096128	0.0175957
rs7095475	-0.0079016	0.00142208	-0.00187	0.002	0.00761	0.002	0.0061	0.0025	-0.0101	0.0089	-0.0105	0.0087	-0.032	0.0428	-0.0162162	0.0194691
rs7098962	0.00606866	0.00106428	-0.000892	0.002	-0.0034	0.002	-0.002	0.0018	-0.0082	0.0067	0	0.0063	-0.0035	0.0317	0.0129035	0.0143781
rs7109373	0.0104065	0.00131812	-0.00491	0.002	-0.00554	0.002	-0.0058	0.0022	0.0171	0.0082	-0.0067	0.0062	-0.0342	0.0385	-0.0042502	0.0181216
rs7131691	-0.0074794	0.00105066	0.00196	0.002	0.00268	0.002	0.0031	0.0018	-0.0145	0.0065	-0.001	0.0051	-0.0084	0.0306	0.0284566	0.0143521
rs7150195	0.0136491	0.00108067	0.000675	0.002	-0.00611	0.002	-0.008	0.0018	0.0087	0.0066	-0.0014	0.0049	0.017	0.0311	0.010533	0.0146763
rs7151326	-0.0086987	0.00107448	-0.00416	0.002	0.00213	0.002	-0.0042	0.0018	-0.0035	0.0066	-0.0011	0.0052	0.0353	0.0314	-0.0107681	0.0147273
rs715694	0.00930045	0.00107837	-0.00259	0.002	-0.00816	0.002	-0.0002	0.0018	0.0174	0.0065	-0.0056	0.0051	-0.0495	0.0311	0.00752763	0.0147153
rs7172133	0.00741954	0.00112881	-0.00162	0.002	-0.00304	0.002	0.0018	0.0019	-0.0008	0.0069	-0.0036	0.0052	-0.0964	0.0322	-0.0042044	0.0151771
rs717997	0.0129679	0.00106141	0.000889	0.002	-0.0051	0.002	-0.0049	0.0018	0.0124	0.0065	-0.0051	0.0051	-0.0737	0.0305	-0.0340292	0.0144221
rs7184582	0.00602571	0.00104833	-0.00591	0.002	-0.00523	0.002	-0.003	0.0017	0.0082	0.0064	-0.001	0.0048	0.0248	0.0301	-0.0026925	0.0141932
rs7187692	0.0110108	0.00104754	0.00228	0.002	-0.00407	0.002	-0.0045	0.0016	0.0013	0.0063	-0.0023	0.0034	0.0222	0.03	0.0114053	0.0142012
rs7196426	0.00912097	0.00141368	0.00118	0.002	-0.00314	0.002	-0.0068	0.0023	-0.004	0.0086	-0.0115	0.0066	0.0424	0.0408	-0.0014164	0.019324
rs719839	-0.0076791	0.00104834	0.000799	0.002	0.00417	0.002	0.0009	0.0017	-0.0008	0.0064	-0.0047	0.0035	-0.1515	0.0306	0.0103435	0.0143182
rs7236339	-0.0153102	0.00126874	-0.00208	0.002	0.0104	0.002	0.0063	0.0021	-0.0187	0.0077	-0.0058	0.0058	0.0011	0.0361	0.016694	0.0170201
rs7254263	-0.0099277	0.00115421	-0.00186	0.002	0.00126	0.002	0.0006	0.0019	-0.0138	0.0071	-0.0037	0.0056	0.0098	0.0337	-0.0063934	0.0157136
rs7297828	0.00877341	0.00119055	0.000703	0.002	0.00584	0.002	-0.0026	0.002	-0.012	0.0072	-0.0075	0.0054	0.0983	0.034	0.0090765	0.016107
rs738988	-0.0160469	0.00115902	0.00159	0.002	0.00114	0.002	0.0034	0.0019	-0.023	0.0071	-0.0064	0.0054	0.0144	0.0332	-0.0105171	0.0155968
rs7522356	0.0119283	0.0010534	-0.000644	0.002	-0.00357	0.002	-0.013	0.0017	0.0085	0.0064	-0.0087	0.0048	0.0818	0.0303	-0.003283	0.0143433
rs752806	-0.007415	0.00105054	-0.00168	0.002	-0.000975	0.002	0.0028	0.0018	-0.0017	0.0065	-0.0002	0.0049	0.0508	0.0305	0.00234646	0.0143241
rs7543481	-0.0060996	0.00110564	-0.00057	0.002	-0.00144	0.002	0.0019	0.0018	-0.0083	0.0068	-0.0017	0.0052	0.0515	0.032	-0.0079594	0.0150482
rs7549469	0.00890771	0.00112135	0.000705	0.002	-0.000991	0.002	0.0001	0.0019	0.0177	0.0069	-0.0009	0.0053	-0.0643	0.0325	0.0104314	0.0152585
rs7571708	0.00894528	0.0010857	-0.00007	0.002	0.00564	0.002	0.0019	0.0018	-0.0034	0.0066	-0.0067	0.0049	-0.0444	0.0314	0.0115228	0.0148263
rs7593817	0.00666549	0.0010809	0.00178	0.002	-0.00415	0.002	-0.0036	0.0018	0.0093	0.0066	-0.0095	0.0052	0.0349	0.0314	-0.0067308	0.0147565
rs7600039	-0.0154077	0.00193205	0.000707	0.003	0.0141	0.003	0.0156	0.0032	-0.0334	0.0112	-0.0107	0.0086	0.1016	0.0559	0.00083918	0.0269593
rs7602601	-0.0104253	0.00161486	-0.00178	0.003	0.00219	0.002	0.0026	0.0027	-0.0112	0.0099	-0.0106	0.0074	0.066	0.0469	0.0421735	0.0221508
rs7626224	0.00784129	0.00122136	0.00343	0.002	-0.00401	0.002	-0.0032	0.002	0.0107	0.0074	-0.0051	0.0061	0.0058	0.0355	-0.0104801	0.0163091
rs7628120	0.0101265	0.00114673	-0.000175	0.002	0.000017	0.002	-0.005	0.0019	0.0125	0.0069	-0.0045	0.0054	-0.0273	0.0334	-0.0215326	0.015766
rs7632819	-0.0130068	0.00121662	0.000506	0.002	0.00188	0.002	0.0087	0.002	-0.0211	0.0073	-0.0097	0.0055	-0.0579	0.0344	-0.000429	0.0161281
rs7641534	-0.0079235	0.00105045	-0.00134	0.002	0.00209	0.002	0.0035	0.0017	-0.0113	0.0064	-0.0059	0.0048	0.0108	0.0305	0.0286489	0.0142685
rs7650118	-0.0063398	0.00111653	-0.00348	0.002	0.00341	0.002	0.0075	0.0018	-0.0151	0.0067	-0.0059	0.005	-0.0365	0.0326	0.0282665	0.0151588
rs7670522	0.0126785	0.00117966	0.0073	0.002	-0.000734	0.002	-0.0008	0.0017	-0.0005	0.0064	-0.0118	0.0049	-0.1132	0.0302	0.00853287	0.0142472
rs7677621	-0.0102896	0.00109483	-0.00221	0.002	0.00679	0.002	0.001	0.0018	-0.0047	0.0067	-0.007	0.0049	0.0747	0.0316	0.0334658	0.0150151
rs7704018	-0.0075855	0.00116077	-0.00221	0.002	-0.00183	0.002	0	0.0019	-0.0072	0.007	-0.002	0.0053	-0.0412	0.0337	0.00774069	0.0159124
rs7715167	-0.0082489	0.0010783	0.000018	0.002	0.00917	0.002	0.0032	0.0018	-0.0024	0.0068	-0.0045	0.0053	0.1222	0.0313	0.00155574	0.0145446
rs7749979	-0.0068482	0.00115	0.0015	0.002	0.006	0.002	0.0017	0.0019	-0.0152	0.007	0	0.0056	-0.0433	0.0333	-0.022389	0.0156242
rs7750668	0.00791108	0.00112144	0.00179	0.002	-0.00139	0.002	0.0031	0.0019	0.0047	0.0069	-0.0032	0.0056	-0.0648	0.0329	-0.0132455	0.0153235
rs7762296	-0.0083505	0.0011365	0.00372	0.002	0.00631	0.002	-0.0024	0.0019	0.0133	0.007	-0.0036	0.0052	-0.0234	0.0333	-0.0130009	0.0155934
rs7778411	0.00609732	0.0010972	-0.000209	0.002	-0.00338	0.002	-0.0065	0.0018	0.0142	0.0067	-0.0021	0.0052	0.0108	0.0318	-0.0007854	0.0149096
rs7803932	0.0116905	0.00141663	0.00427	0.002	0.00329	0.002	-0.0016	0.0023	0.0075	0.0083	-0.0126	0.0062	-0.0317	0.0405	0.0381243	0.0190022
rs7810903	0.0119349	0.00113546	0.00236	0.002	0.00298	0.002	-0.0056	0.0019	0.0045	0.0068	-0.0022	0.0052	-0.0181	0.0326	-0.0242258	0.0153638
rs7815299	0.00777551	0.00111286	-0.0012	0.002	-0.00766	0.002	-0.002	0.0018	0.001	0.0068	-0.0035	0.005	0.0934	0.0322	-0.0146531	0.0151726
rs785278	0.00850969	0.00136963	-0.00234	0.002	-0.00757	0.002	-0.0159	0.0023	0.0314	0.0087	-0.0048	0.0063	-0.0167	0.0398	0.0222092	0.0189714
rs792213	0.00732975	0.00106274	-0.00285	0.002	-0.00457	0.002	0.0026	0.0018	-0.0037	0.0065	-0.0057	0.0049	-0.0072	0.0306	-0.0060442	0.0144128
rs7924465	0.0144836	0.0017999	-0.000336	0.003	0.00221	0.003	-0.0052	0.003	0.0147	0.011	-0.0087	0.0083	0.0289	0.0521	0.0310435	0.0244344
rs7928622	-0.007947	0.00111878	0.000107	0.002	0.00522	0.002	0.0013	0.0019	-0.0063	0.0068	-0.0053	0.0056	-0.0063	0.0329	0.0159939	0.0152558
rs7977614	-0.0111541	0.00117652	0.00363	0.002	0.00656	0.002	0.0009	0.002	0.0095	0.0072	-0.0039	0.008	0.0957	0.0343	0.011983	0.015579
rs7979979	-0.0116479	0.00121864	-0.00382	0.002	0.00165	0.002	0.0036	0.002	-0.0265	0.0075	-0.0085	0.0057	-0.0509	0.035	-0.0026721	0.0163405
rs7986948	0.00878503	0.00157645	0.00219	0.003	0.000099	0.002	-0.0008	0.0026	0.0022	0.0096	-0.0084	0.0075	-0.0059	0.0451	-0.0022015	0.0213529
rs8000862	-0.00679	0.00109122	-0.00211	0.002	-0.000081	0.002	0.0022	0.0018	-0.0035	0.0066	-0.0017	0.005	-0.0007	0.0312	-0.0138914	0.0147173
rs8025575	-0.0080159	0.0010521	0.000737	0.002	0.00216	0.002	-0.0004	0.0017	-0.0082	0.0064	-0.0057	0.0049	0.0213	0.0302	-0.0059929	0.0142686
rs8043948	-0.0062871	0.00104732	-0.000621	0.002	0.00613	0.002	0.0027	0.0017	-0.0028	0.0064	-0.0033	0.0049	-0.0113	0.0301	-0.0050593	0.0142422
rs8044082	0.0071805	0.00106874	0.00323	0.002	0.000429	0.002	-0.0037	0.0017	0.0104	0.0066	-0.0003	0.0035	-0.101	0.0312	0.0169617	0.0146025
rs8055491	-0.0077639	0.00104925	-0.00599	0.002	-0.00268	0.002	-0.0021	0.0017	0.0167	0.0064	-0.0032	0.005	0.0585	0.0302	-0.0024324	0.0142245
rs8072494	-0.0115965	0.00128354	0.00222	0.002	0.00403	0.002	0.0035	0.002	-0.0157	0.0079	-0.0046	0.0042	-0.0083	0.0368	0.00437923	0.0173653
rs8110747	-0.0065921	0.00106499	0.000928	0.002	0.000718	0.002	0.0012	0.0018	0.0038	0.0064	-0.0017	0.0049	-0.0413	0.0306	0.0183032	0.0144755

rs8614	-0.0127362	0.00134439	-0.00701	0.002	0.00987	0.002	0.0017	0.0022	0.027	0.0085	-0.0062	0.0047	-0.0555	0.0393	0.0294621	0.0183944
rs870681	-0.0077872	0.00120089	0.00313	0.002	0.00769	0.002	0.0061	0.002	-0.0075	0.0075	-0.0058	0.0056	0.0738	0.0346	-0.0076086	0.0162193
rs876475	0.00931195	0.00107113	-0.00163	0.002	-0.00809	0.002	-0.0032	0.0018	-0.0022	0.0065	-0.0057	0.0048	0.0173	0.0306	0.00313395	0.0144801
rs886765	-0.0107709	0.00191074	0.00448	0.003	0.0059	0.003	0.007	0.0032	0.0059	0.0121	-0.0077	0.0086	0.0942	0.0564	0.0192239	0.0263271
rs899223	0.0133241	0.00193013	0.00337	0.003	-0.00273	0.003	0.001	0.0034	-0.0103	0.0121	-0.0042	0.0131	-0.0321	0.0587	-0.0250627	0.0264012
rs902712	0.010805	0.00118054	0.00142	0.002	-0.005	0.002	-0.0102	0.0019	-0.015	0.0072	-0.0097	0.0054	-0.0729	0.0341	-0.0263747	0.0159921
rs912609	0.0123495	0.00125321	0.0015	0.002	-0.00336	0.002	-0.0018	0.0021	0.0133	0.0075	-0.0085	0.0057	-0.0247	0.0358	-0.0474866	0.0169262
rs9267576	0.018157	0.00152404	0.00486	0.003	0.000988	0.002	-0.0046	0.0023	0.0701	0.0091	-0.001	0.0049	-0.3466	0.0438	0.0432804	0.0200768
rs9291436	0.00854282	0.00108719	0.000963	0.002	-0.00321	0.002	0.0002	0.0018	0.007	0.0067	-0.0062	0.0053	-0.0676	0.0317	0.0142943	0.0148875
rs9297016	0.0112169	0.00106881	0.000623	0.002	0.000243	0.002	-0.0002	0.0018	0.0106	0.0065	-0.0017	0.0049	-0.0395	0.0306	-0.0069048	0.0144735
rs9317202	0.00978591	0.00114495	-0.000541	0.002	-0.00262	0.002	-0.0036	0.0019	0.0109	0.0069	-0.0102	0.0054	-0.0202	0.033	0.0247949	0.0157277
rs9321394	0.00919212	0.00121118	-0.000559	0.002	-0.00515	0.002	-0.0023	0.002	0.0088	0.0073	-0.0037	0.0058	-0.1192	0.0351	0.0225007	0.0165422
rs9373363	-0.0106184	0.00121696	0.00391	0.002	0.00624	0.002	0.0064	0.002	-0.0254	0.0073	-0.0076	0.006	0.191	0.0349	-0.0025869	0.0164596
rs9374194	-0.0088106	0.00133461	-0.0032	0.002	-0.00622	0.002	0.0014	0.0022	-0.0123	0.0081	-0.0105	0.0059	-0.0418	0.038	0.0380885	0.0178857
rs9388490	0.0115001	0.00105233	0.00265	0.002	-0.00724	0.002	-0.0024	0.0017	-0.0503	0.0064	-0.0029	0.0048	0.0764	0.0305	-0.0211378	0.0143199
rs9393415	0.0138342	0.0011326	0.00474	0.002	-0.00702	0.002	-0.0075	0.0019	0.0286	0.0071	-0.0118	0.0053	-0.0759	0.0332	-0.008873	0.0156638
rs9410471	-0.0147498	0.00171329	0.00907	0.003	0.00686	0.003	-0.0007	0.0029	-0.0098	0.0107	-0.0103	0.0079	0.0274	0.0503	0.00558912	0.0233045
rs9411336	-0.0128496	0.00111875	0.00153	0.002	0.00998	0.002	0.0095	0.0018	-0.0248	0.0069	-0.0028	0.005	-0.0272	0.0321	0.0258578	0.0152106
rs9426674	-0.007612	0.00106951	0.00314	0.002	0.00423	0.002	-0.0002	0.0018	-0.0003	0.0066	-0.0002	0.005	-0.0324	0.0308	-0.0063318	0.0145175
rs9446446	-0.0058273	0.00104657	0.000472	0.002	0.00518	0.002	0.001	0.0017	-0.0008	0.0064	-0.0002	0.004	-0.0194	0.0302	-0.0291297	0.0142581
rs9467804	-0.0106716	0.00104797	0.000594	0.002	0.00555	0.002	0.0026	0.0017	-0.0036	0.0065	-0.0022	0.0048	-0.1641	0.0305	0.0115275	0.0142561
rs9540731	0.0107463	0.0010467	0.00262	0.002	-0.0117	0.002	-0.0058	0.0017	0.0108	0.0064	-0.002	0.0048	-0.0137	0.0301	-0.0113743	0.0142006
rs9540920	0.0058384	0.00105755	-0.000524	0.002	-0.00504	0.002	0.0105	0.0017	-0.0028	0.0064	-0.006	0.0048	-0.0887	0.0305	0.019178	0.0143298
rs958131	0.0100289	0.0010708	-0.00198	0.002	-0.00417	0.002	0.0015	0.0018	0.0017	0.0065	-0.0106	0.0049	-0.0287	0.0307	0.0268468	0.0145479
rs9591001	0.00767379	0.00108732	-0.000872	0.002	-0.00328	0.002	-0.0157	0.0018	0.0173	0.0067	-0.0014	0.0049	-0.0264	0.0311	-0.0019414	0.0147818
rs9595590	0.00788553	0.00104693	0.0018	0.002	0.000223	0.002	-0.0046	0.0017	0.0002	0.0064	-0.0112	0.0048	-0.0493	0.0299	-0.0388112	0.0142246
rs9635366	0.016096	0.0013575	-0.000259	0.002	-0.00276	0.002	-0.0104	0.0022	0.0032	0.0082	-0.0133	0.006	-0.0981	0.0388	0.0187728	0.0184628
rs9764	0.0111651	0.00118661	-0.00429	0.002	-0.00364	0.002	-0.0072	0.0019	0.0041	0.0074	-0.0042	0.0054	-0.1126	0.0341	-0.0201951	0.0160368
rs9787076	-0.0183804	0.00111375	0.00228	0.002	0.0151	0.002	0.001	0.0018	-0.0028	0.0067	-0.0047	0.0051	-0.1036	0.032	-0.0010219	0.0151217
rs9827101	-0.0083622	0.00104818	-0.00335	0.002	0.00375	0.002	0.0056	0.0017	0	0.0064	-0.0044	0.0048	0.0359	0.0303	-0.0315492	0.0142477
rs9869597	-0.0087341	0.0012754	0.00335	0.002	0.00664	0.002	0.0038	0.0022	0.0012	0.0079	-0.0069	0.0058	0.0328	0.0374	0.00744951	0.017816
rs9871964	-0.009507	0.00137905	0.00267	0.002	-0.0031	0.002	0.0008	0.0023	0.0047	0.0086	-0.0025	0.0065	0.0431	0.0399	-0.0103327	0.0187739
rs9880138	-0.0103951	0.00181218	0.00264	0.003	0.00672	0.003	-0.0046	0.003	0.0194	0.0109	-0.001	0.0084	0.108	0.0529	-0.0137228	0.0250576
rs9886703	-0.0112291	0.00140316	-0.00816	0.002	0.00356	0.002	0.0054	0.0023	-0.0187	0.0086	-0.0042	0.0068	-0.1328	0.0404	0.00431352	0.0190723
rs989996	-0.0094157	0.00104974	-0.0015	0.002	0.0103	0.002	0.0017	0.0016	-0.0154	0.0064	-0.0029	0.0034	0.1013	0.0305	-0.0066458	0.0143523
rs991001	0.00979859	0.00153382	0.00235	0.003	0.000952	0.002	-0.0006	0.0026	-0.0133	0.0095	-0.0023	0.0071	0.0164	0.0452	0.0115456	0.0213259
rs9915323	-0.0113425	0.0011493	-0.0064	0.002	0.01	0.002	0.0022	0.0019	-0.0293	0.0071	-0.0352	0.0054	-0.0754	0.0334	0.0171646	0.0158199
rs9922788	0.0102792	0.00105583	0.00161	0.002	-0.00304	0.002	-0.0014	0.0017	0.0012	0.0064	-0.0023	0.005	-0.0951	0.0303	0.00531192	0.0142824
rs9937449	0.00777199	0.00105311	-0.000139	0.002	-0.00176	0.002	-0.003	0.0018	0.0015	0.0063	-0.0034	0.0049	0.0058	0.0303	0.00174921	0.0143329
rs9949224	0.00698484	0.00104711	0.00192	0.002	-0.00545	0.002	-0.0016	0.0017	0.0163	0.0064	-0.0049	0.0049	-0.0157	0.0302	-0.0130539	0.0142977
rs9956721	-0.0132024	0.00106226	-0.00433	0.002	0.00539	0.002	0.0073	0.0018	-0.0079	0.0064	-0.0113	0.0049	-0.1182	0.0306	-0.0079283	0.0144215
rs9985296	-0.0103135	0.00107676	-0.00192	0.002	0.00109	0.002	0.0059	0.0018	-0.0203	0.0065	-0.0074	0.0056	0.0121	0.031	0.0104471	0.0146401
rs9992967	0.00820093	0.00127583	-0.00644	0.002	0.0022	0.002	-0.0039	0.0021	-0.0013	0.0077	-0.0084	0.0058	0.0921	0.0367	-0.0018553	0.0174298

SNP, single nucleotide polymorphism; Edu, educational attainment; SE, standard error; Alc, alcohol consumption; Smk, smoking initiation; BMI, body mass index; T2DM, type 2 diabetes; HDL, high-density lipoprotein cholesterol; SBP, systolic blood pressure

References

1. Rudd KE, Johnson SC, Agesa KM, Shackelford KA, Tsoi D, Kievlan DR, Colombara DV, Ikuta KS, Kissoon N, Finfer S et al. Global, regional, and national sepsis incidence and mortality, 1990-2017: analysis for the Global Burden of Disease Study. Lancet. 2020;395(10219):200-211.
